# Supplementary material for: Antigenotoxic, Anti-photogenotoxic, and Antioxidant Properties of Polyscias filicifolia Shoots Cultivated In Vitro
Source: Molecules. 2020 Feb 28;25(5):1090. doi: 10.3390/molecules25051090 (PMC7179227; doi:10.3390/molecules25051090)
Supplement: Supplementary file 1 [file molecules-25-01090-s001.pdf]

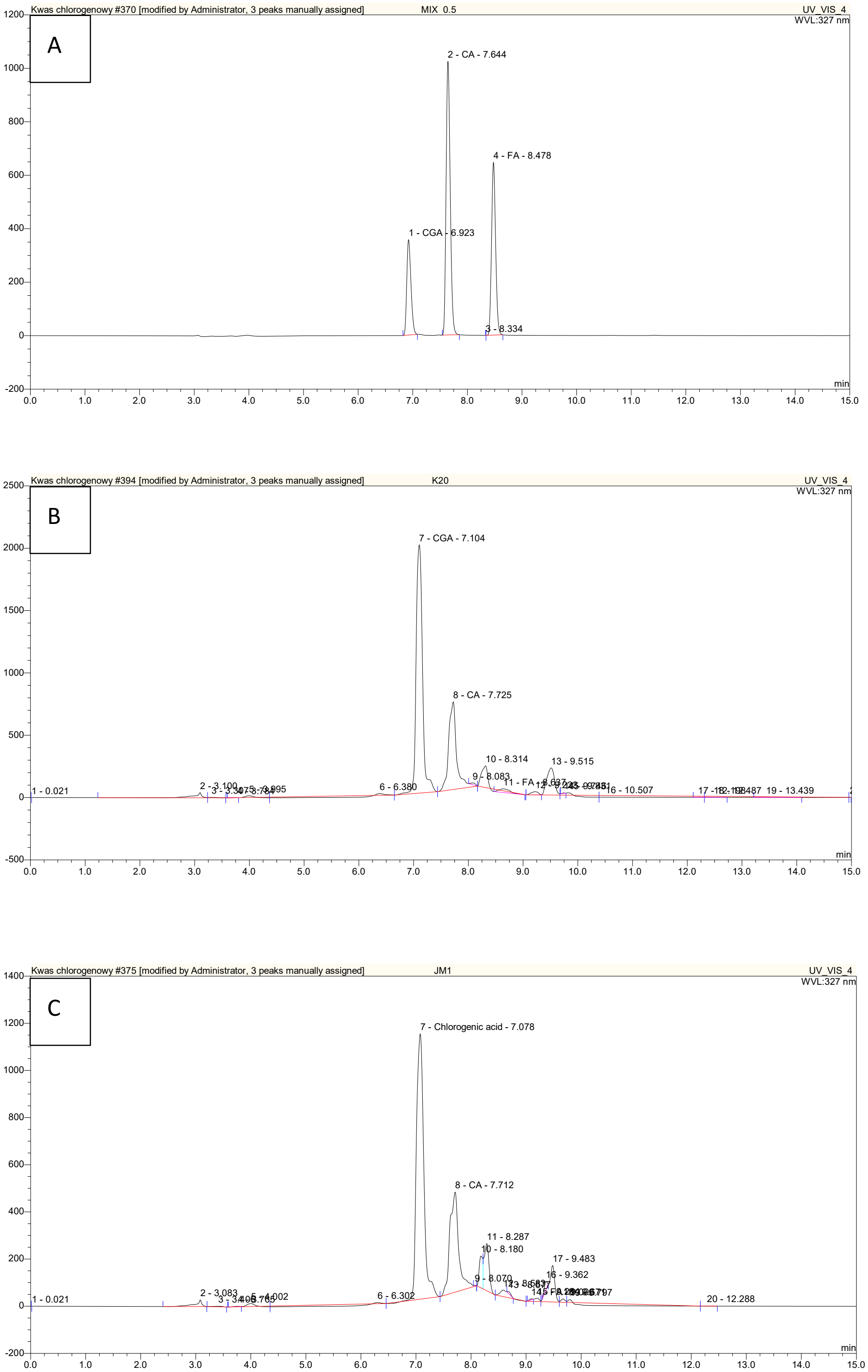
Kwas chlorogenowy #370 [modified by Administrator, 3 peaks manually assigned]
MIX_0.5
UV_VIS_4
WVL:327 nm
A
2 - CA - 7.644
4 - FA - 8.478
1 - CGA - 6.923
3 - 8.334
min
Kwas chlorogenowy #394 [modified by Administrator, 3 peaks manually assigned]
K20
UV_VIS_4
WVL:327 nm
B
7 - CGA - 7.104
8 - CA - 7.725
10 - 8.314
13 - 9.515
9 - 8.083
1 - 0.021
2 - 3.100
6 - 6.380
16 - 10.507
19 - 13.439
min
Kwas chlorogenowy #375 [modified by Administrator, 3 peaks manually assigned]
JM1
UV_VIS_4
WVL:327 nm
C
7 - Chlorogenic acid - 7.078
8 - CA - 7.712
11 - 8.287
10 - 8.180
17 - 9.483
16 - 9.362
9 - 8.070
1 - 0.021
2 - 3.083
6 - 6.302
20 - 12.288
min

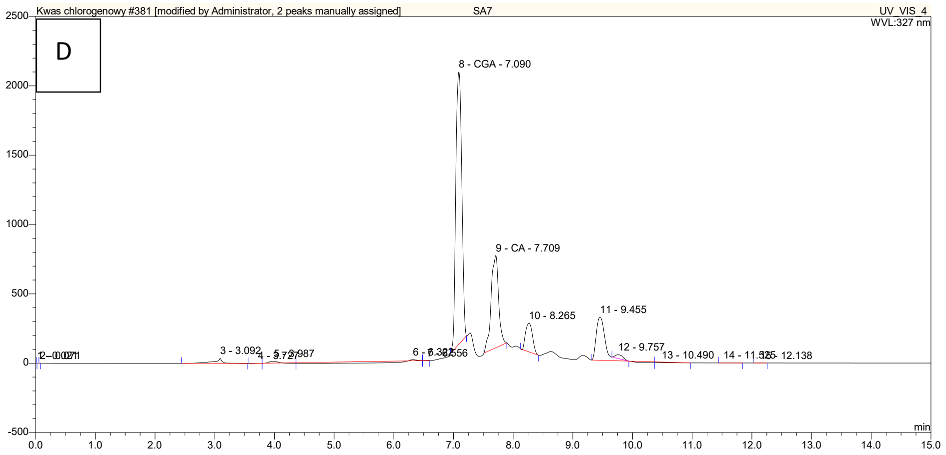

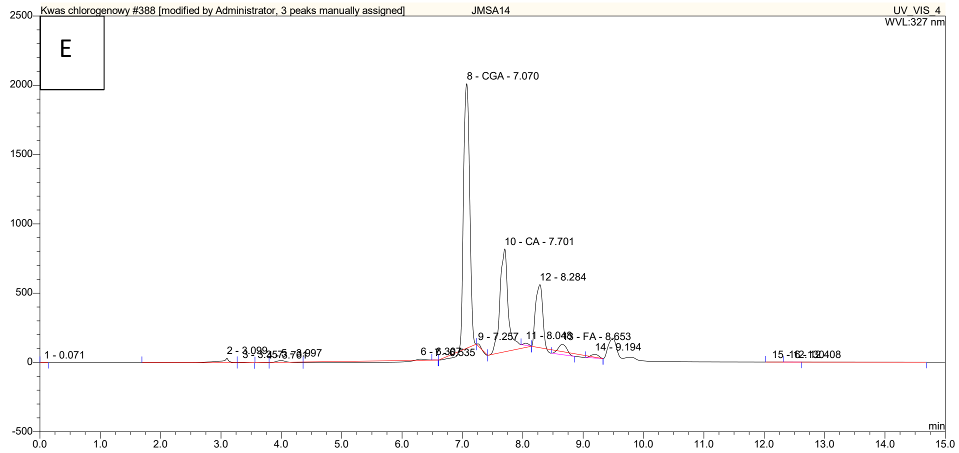

Figure 1S. HPLC-UV-Vis chromatograms of plant extracts subjected to the investigation of antigenotoxic, anti-photogenotoxic and antioxidant properties: A) chromatogram of standard compounds: chlorogenic acid (CGA), caffeic acid (CA) and ferulic acid (FA) λ=327; B) methanolic extract of shoots cultivated under control conditions (EXT0); C) methanolic extract of shoots elicited with methyl jasmonate 200 µM (EXT(MeJA); D) methanolic extract of shoot elicited with salicylic acid 50 µM (EXT(SA); E) methanolic extract of shoot elicited with salicylic acid 50 µM and methyl jasmonate 200 µM (EXT(MeJA+SA)).

Table S1. UHPLC-DAD-MS/MS analysis of investigated *Polysicias filicifolia* methanolic extracts

	Rt (min)	Meas. m/z	Ion Formula	[M-H] m/z	mSigma	MS-MS	UV (nm)	Identification	Area Frac. %
**Extract from control untreated cultures**									
1	0.7	133.014317	C4H5O5	133.014247	7.6	133, 115	195, 219	L-malic acid	22.49
2	0.9	128.035314	C5H6NO3	128.035317	1.2	128, 117	195, 267	4-oxoproline	2.71
3	1.0	297.119231	C11H21O9	297.119106	1.8	297, 265, 251	not detected	unidentified	2.98
4	1.3	429.161153	C16H29O13	429.161365	3.6	429, 383, 256, 164	not detected	unidentified	2.44
5	1.5	399.150809	C15H27O12	399.150800	1.6	399 (353+46 HCOOH), 353	not detected	1/3/5-O-caffeoylguinic acid (chlorogenic acid)	0.9
6	1.8	353.087912	C16H17O9	353.087806	5.1	353, 311, 256	215, 323	1/3/5-O-caffeoylguinic acid (chlorogenic acid)	1.8
7	1.9	203.082459	C11H11N2O2	203.082601	7.0	203	219.279	L-tryptophan	3.03
8	2.7	353.088329	C16H17O9	353.087806	3.2	353, 191	219, 323	1/3/5-O-caffeoylguinic acid (chlorogenic acid)	0.83
9	2.9	353.088154	C16H17O9	353.087806	11.6	353, 173, 135	215, 323	1/3/5-O-caffeoylguinic acid (chlorogenic acid)	1.88

10	3.6	447.151738	C19H27O12	447.150800	0.8	447, 337, 191	211, 311	8-O-Acetyl shanzhiside methyl ester (barlerin)	3.16
11	4.4	367.104074	C17H19O9	367.103456	8.9	367, 191	219, 323	5-O-Feruloylquinic acid	1.15
12	4.8	361.151381	C17H21N4O5	361.151743	8.8	361, 199, 155	not detected	unidentified	0.72
13	4.9	473.167012	C21H29O12	473.166450	4.5	473, 341, 191	not detected	unidentified	0.71
14	5.2	367.104315	C17H19O9	367.104793	14.4	367, 191	215, 315	quinic acid derivative	0.81
15	5.7	523.219835	C26H35O11	523.218486	7.3	523, 461, 361, 146	219, 323	unidentified	0.89
16	6.3	515.177631	C23H31O13	515.177015	8.8	515, 179	219, 327	caffeic acid derivative	1.45
17	6.7	517.229103	C22H25N14O2	517.229040	7.1	517, 441, 395, 263, 161	not detected	unidentified	0.92
18	6.8	515.120124	C25H23O12	515.119500	2.6	515, 353, 191	219, 327	3,5-O- dicaffeoylguinic acid	0.86
19	6.9	187.097877	C9H15O4	187.097583	8.1	187, 169, 125	not detected	unidentified	1.13
20	7.3	565.192477	C25H21N14O3 ?	565.192654	18.3	565, 519, 461, 300, 179	not detected	unidentified	0.78
21	8.0	529.1927	C24H33O13	529.192665	10.8	529, 417, 299, 171	not detected	unidentified	0.88

22	8.5	669.276910	C32H45O15	669.276394	13.1	669, 529, 461, 243	not detected	unidentified	1.1
23	8.8	583.2338	C28H39O13	583.239615	5.7	583, 537, 461, 257	not detected	unidentified	0.96
24	9.5	193.050403	C10H9O4	193.050632	7.7	385, 313, 193	223, 323	ferulic acid derivative	1.24
25	10.0	729.295905	C34H49O17	729.297524	3.7	729, 575, 457, 284	not detected	unidentified	0.88
26	10.4	597.254438	C29H41O13	597.255265	6.4	597 (551+46HCOOH), 551(389+162hexose), 389, 161	not detected	unidentified	2.37
27	11.2	593.2595	C30H41O12	593.260350	19.0	593(547+46HCOOH), 547, 457, 213, 146	not detected	unidentified	1.03
28	11.7	577.134815	C30H25O12	577.135150	7.2	577,385,282,193	219, 323	catechin derivative	1.19
29	12.1	845.323530	C42H53O18	845.323738	15.1	845, 577, 439, 327	not detected	unidentified	0.71
30	12.5	173.118319	C9H17O3	173.118318	8.4	439, 327, 173	not detected	unidentified	0.72

31	13.0	577.136210	C30H25O12	577.135150	8.6	577, 441, 327, 146	not detected	(+) - procyanidin B2 (Epi catechin dimer)	1.19
32	13.3	577.135092	C30H25O12	577.135150	2.6	577, 533, 341, 282, 134	223, 323	(+) - procyanidin B2 (Epi catechin dimer)	1.41
33	13.7	569.297306	C29H45O11	569.296736	3.5	569, 457, 329, 187	not detected	dammarenolic acid-type triterpenoid	1.6
34	14.1	581.270608	C55H87O26	581.270915	11.5	1117, 731, 581, 523, 455, 329	not detected	oleanane-type triterpenoid	1
35	14.3	566.266013	C54H84O25	566.265633	17.0	1087, 715, 566, 455, 327, 191	not detected	oleanane-type triterpenoid	1.69
36	14.5	199.134322	C11H19O3	199.133968	3.4	581, 399, 199	not detected	unidentified	0.84
37	14.7	566.265960	C53H83O23	566.266301	29.2	1087, 566, 455, 325, 146	not detected	oleanane-type triterpenoid (Polyscioside E)	1.43
38	15.3	955.4894	C48H75O19	955.489522	128.9	955, 775, 613, 569, 500 (-2), 309	not detected	medicagenic acid- type triterpenoid ?	0.95
39	15.9	179.144392	C12H19O	179.144139	6.8	223, 179	not detected	unidentified	1.01
40	17.1	203.144383	C14H19O	203.144139	1.5	203	not detected	unidentified	0.79

41	17.9	955.490054	C48H75O19	955.490804	12.9	955, 731, 523, 455, 309, 146	not detected	oleanane-type triterpenoid (Polyscioside C/ Polyscioside A)	3.44
42	18.5	925.478748	C47H73O18	925.480239	12.3	925, 577, 447, 309, 146	not detected	oleanane-type triterpenoid (Polyscioside B)	0.89
43	18.9	311.222611	C18H31O4	311.222783	71.4	311, 146	not detected	octadecadienoic acid ?	0.78
44	19.8	793.4363	C42H65O14	793.437980	17.3	793, 311, 146	not detected	spinasaponin A/ zingibroside R1 (oleanane-type triterpenoid)	1.11
45	20.8	293.211754	C18H29O3	293.212218	8.0	293, 275, 235, 171	not detected	13-Hydroxy-6,9,11-octadecatrienoic acid	3
46	21.0	293.2116	C18H29O3	293.209533	13.5	293, 275, 247, 223, 195	not detected	Hydroxy-octadecatrienoic acid (isomer)	0.81

47	21.6	353.199865	C19H29O6	353.196962	11.1	353, 295, 220, 112	215, 271, 283	unidentified	4.3
48	22.2	295.2272	C18H31O3	295.227868	15.2	295, 277, 195, 171	not detected	cis-12,13-Epoxy-cis-9-octadecenoic acid	2.88
49	22.9	187.148787	C14H19	187.149224	0.8	187	not detected	unidentified	4.41
50	23.0	293.211748	C18H29O3	293.212218	1.1	293, 185	not detected	Hydroxy-octadecatrienoic acid (isomer)	1.66
51	26.7	375.275927	C20H39O6	375.275213	8.6	375, 343, 223, 182	not detected	octadecanoic acid derivative	1.14
52	26.9	279.233309	C18H31O2	279.232954	2.9	279	not detected	linoleic acid	0.95
**Extract from shoots treated with SA 100 μM**									
1	0.7	133.014306	C4H5O5	133.014247	6.7	133, 115	195, 215	L-malic acid	40.52
2	0.9	128.035379	C5H6NO3	128.035317	4.6	128, 117	195, 263	4-oxoproline	7.31
3	1.1	385.134679	C14H25O12	385.135150	1.9	385 (339+46HCOOH), 339, 251	not detected	unidentified	10.30
4	1.9	203.082512	C11H11N2O2	203.082601	10.2	203	219.279	L-tryptophan	0.76

5	2.2	351.129848	C14H23O10	351.129671	3.8	351, 252, 167	not detected	unidentified	1.42
6	2.7	353.087987	C16H17O9	353.087806	8.7	353, 191	219, 323	1/3/5-O-caffeoylguinic acid (chlorogenic acid)	1.13
7	2.8	353.087856	C16H17O9	353.087806	3.2	353, 179	215, 323	1/3/5-O-caffeoylguinic acid (chlorogenic acid)	1.16
8	2.9	413.166537	C16H29O12	413.166450	0.7	413 (367+46HCOOH), 367	not detected	unidentified	1.01
9	3.6	337.093169	C16H17O8	337.092891	2.6	447, 393, 337, 191	not detected	1-Caffeoyl-4-deoxyquinic acid	6.98
10	4.4	367.103648	C17H19O9	367.103456	1.4	367, 191	219, 323	5-O-Feruloylquinic acid	0.57
11	4.5	337.093271	C16H17O8	337.092891	6.2	337, 191	215, 303	Caffeoyl-deoxyquinic acid (izomer)	0.66
12	5.1	367.103596	C17H19O9	367.103456	8.4	367, 191	215	quinic acid derivative	0.48
13	5.3	741.188128	C32H37O20	741.188357	14.5	741, 539, 379, 300, 178	215	unidentified	1.16
14	5.7	559.145705	C27H27O13	559.145715	13.0	559, 523, 479	219, 323	unidentified	3.32

15	5.8	609.1463	C27H29O16	609.146108	10.0	609, 427, 301, 223, 153	211, 261, 351	quercetin 3-O-rhamnoglucoside (rutin)	0.65
16	6.2	565.1925	C27H33O13	565.192665	6.4	565, 479, 387, 146	219	unidentified	0.45
17	6.3	515.177153	C23H31O13	515.177015	1.4	515, 179	219, 327	caffeic acid derivative	0.33
18	6.8	515.119180	C25H23O12	515.119500	2.6	515, 353, 191	219, 327	3,5-O- dicaffeoylguinic acid	0.57
19	7.2	623.161473	C28H31O16	623.161758	4.3	623, 461, 315	219	methylquercetin derivative (narcisin)	1.29
20	7.3	561.160641	C27H29O13	561.161365	13.0	561, 357, 325, 191	219, 323	feruloylquinic acid derivative	1.71
21	8.4	669.2751	C32H45O15	669.275046	7.5	669, 529, 461, 367, 146	219	unidentified	0.84
22	8.8	583.23903	C28H39O13	583.239615	12.8	583(537+46HCOOH), 537(375+162hexose), 375, 146	199, 219	unidentified	0.61
23	9.5	193.050334	C10H9O4	193.050632	15.6	385, 313, 193	219, 327	ferulic acid derivative	0.98
24	9.6	579.243261	C29H39O12	579.243352	22.8	579, 519, 435, 375, 161	219	unidentified	0.57

25	9.9	729.295905	C34H49O17	729.297524	3.7	729, 683, 575, 491, 146	219	unidentified	0.52
26	10.2	365.195842	C17H21N10	365.195614	6.5	365, 321, 303	219	unidentified	0.92
27	10.4	597.253650	C29H41O13	597.253917	6.6	597(551+46HCOOH), 551(389+162hexose), 389, 161	223	unidentified	0.47
28	11.2	593.259204	C30H41O12	593.260350	9.1	593, 547, 389, 326, 161	223	unidentified	2.58
29	11.7	577.134463	C30H25O12	577.135150	7.1	577, 385, 282, 193	219, 323	catechin derivative?	0.43
30	12.1	845.321456	C42H53O18	845.320540	17.1	845, 577, 499, 439, 146	not detected	unidentified	0.56
31	13.0	577.134405	C30H25O12	577.135150	7.9	577, 385, 282, 146	219, 267, 323	(+) - procyanidin B2 (Epi catechin dimer)	0.71
32	13.2	577.134287	C30H25O12	577.135150	10.4	577, 445, 341, 282, 146	223, 267, 323	(+) - procyanidin B2 (Epi catechin dimer)-isomer	1.30
33	13.9	581.270031	C55H87O26	581.270915	14.8	1117, 731, 581, 523, 457, 329	not detected	dammarenolic acid-type triterpenoid	1.41

34	15.3	955.489541	C48H75O19	500.244503	5.3	955, 775, 613, 569, 500 (-2), 309	not detected	medicagenic acid- type triterpenoid ?	1.42
35	17.9	955.4895	traces			955, 535, 455, 309, 146	not detected	oleanane-type triterpenoid (Polyscioside C/ Polyscioside A)	0.70
36	18.1	955.4908	traces			955, 529, 449, 309, 146	not detected	oleanane-type triterpenoid	0.60
37	19.0	265.148076	C15H21O4	265.144533	22.6	265	not detected	unidentified	0.54
38	19.8	793.437351	C42H65O14	793.437980	12.9	793, 613, 531, 311, 146	not detected	spinasaponin A/ zingibroside R1 (oleanane-type triterpenoid)	0.52
39	20.7	721.364553	C34H57O16	721.365209	14.6	721, 397, 277, 179	not detected	unidentified	0.84
40	20.9	595.288397	C29H43N2O11	595.287234	6.9	595, 311, 146	not detected	unidentified	0.37
41	21.6	571.288344	C27H43N2O11 ?	571.287234	16.0	571, 391, 353, 146	not detected	unidentified	0.11
42	21.9	723.379286	C34H59O16	723.379511	4.5	723, 507, 397, 305	not detected	unidentified	0.75

43	22.1	564.330657	C30H42N7O4?	564.330376	35.4	564, 481, 397, 293, 146	not detected	unidentified	0.47
44	22.2	295.227039	C18H31O3	295.227868	11.5	559?, 295, 277, 195, 171	not detected	cis-12,13-Epoxy-cis-9-octadecenoic acid	-
45	22.9	187.148408	C14H19	187.149224	9.2	433?, 187	not detected	unidentified	-
46	24.9	513.305083	C27H45O9	513.306907	1.9	513, 403, 277, 227	not detected	unidentified	-
47	26.0	515.321136	C27H47O9	515.321209	10.2	515, 437, 379, 279	not detected	octadecanoic acid derivative	-
48	26.7	491.321403	C25H47O9	491.321209	3.9	491, 375, 297, 192	not detected	palmitate acid derivative	-
**Extract from shoots treated with MeJA 100 μM**									
1	0.7	133.014261	C4H5O5	133.014247	2.0	133, 115	195, 215	L-malic acid	37.77
2	0.9	128.035185	C5H6NO3	128.035317	1.6	128, 117	195, 267	4-oxoproline	5.04
3	1.0	385.134559	C14H25O12	385.135150	3.2	385 (339+46HCOOH), 339, 297	not detected	unidentified	8.04
4	1.9	203.081719	C11H11N2O2	203.082601	0.6	203	219.279	L-tryptophan	0.93
5	2.2	351.128512	C14H23O10	351.128323	6.0	351, 256, 207, 161	not detected	unidentified	1.29

6	2.7	353.087161	C16H17O9	353.087806	2.7	353, 191	219, 323	1/3/5-O-caffeoylguinic acid (chlorogenic acid)	0.37
7	2.9	413.165590	C16H29O12	413.166450	10.0	413 (367+46HCOOH), 367	not detected	unidentified	0.23
8	3.6	387.165536	C18H27O9	387.166056	18.4	387, 337(191+146 deoxyhexose), 191	211, 311	derivative quinic acid?	5.58
9	4.3	367.103648	C17H19O9	367.103456	1.4	367, 191	219, 323	5-O-Feruloylquinic acid	0.5
10	4.5	337.091671	C16H17O8	337.092891	1.1	337, 191	215, 303	derivative caffeoylquinic acid	0.58
11	4.7	429.175691	C20H29O10	429.176621	2.2	429, 391, 227	not detected	unidentified	1.38
12	5.1	367.101687	C17H19O9	367.103456	3.4	367, 191	215, 315	quinic acid derivative	1.73
13	5.3	741. 186483	C32H37O20	741.188367	15.3	741, 539, 367, 300, 165	219	unidentified	0.24
14	5.7	559.143507	C27H27O13	559.145715	17.2	559, 523, 479	219, 323	unidentified	3.07
15	5.8	609.143787	C27H29O16	609.146108	3.4	609, 427, 301, 223, 153	215, 261, 347	quercetin 3-O-rhamnoglucoside (rutin)	0.24

16	6.3	515.175031	C23H31O13	515.177015	10.1	515, 179	219, 327	caffeic acid derivative	0.31
17	6.8	515.117859	C25H23O12	515.119500	5.1	515, 353, 191	219, 327	3,5-O- dicaffeoylguinic acid	0.28
18	7.2	623.150966	C28H31O16	623.161758	12.4	623, 461, 315	219	methylquercetin derivative (narcisin)	1.11
19	7.4	561.159802	C27H29O13	561.161365	14.5	561, 357, 325, 191	219, 323	feruloylquinic acid derivative	0.33
20	7.8	373.185393	C18H29O8	373.186791	9.1	373, 211	not detected	unidentified	0.37
21	8.0	417.175108	C19H29O10	417.176621	16.5	417, 373, 209, 193, 175, 134	not detected	unidentified	0.31
22	8.4	669.275167	C32H45O15	669.276394	11.8	669 (537+132pentose), 537, 529, 413, 146	219, 327	unidentified	0.78
23	8.4	917.233912	C42H45O23	917.235711	16.9	917, 741, 609, 300	219, 327	unidentified	1.6
24	8.8	583.238421	C28H39O13	583.239615	4.5	583 (537+46HCOOH), 537, 461, 355, 193	203, 219?	unidentified	1.32

25	9.1	415.196120	C20H31O9	415.197356	5.1	415, 373, 211, 193	not detected	unidentified	0.68
26	9.5	193.049890	C10H9O4	193.050632	11.3	407, 385, 313, 193	219, 323	ferulic acid derivative	0.36
27	9.6	579.243169	C29H39O12	579.243352	12.7	579, 519, 415, 375	223	unidentified	0.68
28	9.9	729.295905	C34H49O17	729.297524	3.7	729(683+46HCOOH), 683(551+132pentose), 551(389+162hexose), 389, 233, 191, 149	223	unidentified	0.6
29	10.4	597.254149	C29H41O13	597.255265	13.4	597 (551+46HCOOH), 551(389+162hexose), 389, 341, 161, 113	223	unidentified	0.83
30	10.9	595.259864	C26H43O15	595.260744	6.9	595(549+46HCOOH), 549(387+162hexose), 387, 355, 265, 161	223	unidentified	0.96

31	11.2	593.259941	C30H41O12	593.260350	14.7	593(547+46HCOOH), 547, 389, 265, 161	223	unidentified	0.37
32	11.7	577.134172	C30H25O12	577.135150	7.1	577, 385, 282, 193	219, 323	catechin derivative	0.33
33	12.1	845.323796	C42H53O18	845.323738	5.5	845, 693, 583, 499, 433, 221	not detected	unidentified	0.46
34	12.4	397.186655	C20H29O8	397.186791	6.7	397, 355, 265, 211	not detected	unidentified	0.84
35	12.6	609.276875	C27H45O15	609.276394	8.8	609, 563, 429, 355, 265, 161	not detected	unidentified	0.93
36	12.7	429.212943	C21H33O9	429.213006	9.7	429, 387, 295, 193,161	not detected	unidentified	0.49
37	13.0	577.135036	C30H25O12	577.135150	7.1	577, 447, 385, 282, 193	223, 267, 323	(+) - procyanidin B2 (Epi catechin dimer)	0.41
38	13.2	577.135036	C30H25O12	577.135150	24.2	577, 533, 341, 282, 179	223, 268, 323	(+) - procyanidin B2 (Epi catechin dimer)-isomer	0.29
39	13.7	569.297223	C29H45O11	569.296736	12.0	569, 457, 329, 187	not detected	dammarenolic acid-type triterpenoid	3.1

40	14.0	581.270837	C55H87O26	581.270915	14.4	1117, 731, 581, 523, 455, 329	not detected	oleanane-type triterpenoid	0.46
41	14.4	443.228888	C22H35O9	443.228656	17.2	443, 404, 337, 309	not detected	unidentified	1.79
42	15.3	955.489564	C48H75O19	955.490804	7.1	955, 775, 613, 569, 500 (-2), 309	not detected	medicagenic acid- type triterpenoid ?	0.59
43	17.8	377.200383	C21H29O6	377.196962	27.0	377, 146	not detected	unidentified	1.93
44	17.9	955.4895	traces			955, 535, 449, 309, 146	not detected	oleanane-type triterpenoid (Polyscioside C/ Polyscioside A)	0.36
45	19.8	793.434940	C42H65O14	793.437980	4.8	793, 613, 531, 311, 146	not detected	spinasaponin A/ zingibroside R1 (oleanane-type triterpenoid)	0.64
46	26.7	375.274144	C20H39O6	375.275213	33.7	375, 297, 152	not detected	octadecanoic acid derivative	0.27
**Extracts from shoots treated with SA (100 μM) and MeJA (100 μM)**									
1	0.7	133.014067	C4H5O5	133.014247	6.4	133, 115	195, 215	L-malic acid	38.28

2	0.9	128.035437	C5H6NO3	128.035317	2.3	128, 117	195, 267	4-oxoproline	6.37
3	1.1	385.134987	C14H25O12	385.135150	7.7	385 (339+46HCOOH), 339, 207	not detected	unidentified	10.35
4	2.2	351.129823	C14H23O10	351.129671	6.5	351, 252, 178	not detected	unidentified	1.20
5	2.7	353.088465	C16H17O9	353.087806	0.7	353, 191	219, 323	1/3/5-O-caffeoylguinic acid (chlorogenic acid)	1.11
6	3.6	387.166835	C18H27O9	387.166056	8.7	387, 337(191+146 deoxyhexose), 191	215, 311	derivative quinic acid?	1.03
7	4.3	367.103815	C17H19O9	367.103456	4.6	367, 191	219, 323	5-O-feruloylquinic acid	5.78
8	4.8	669.205189	C30H37O17	669.203623	6.6	669, 551, 455, 433, 293, 179	219.327	unidentified	0.43
9	5.1	367.104586	C17H19O9	367.103456	11.7	367, 191	215, 315	3-O-feruloylquinic acid	0.68
10	5.8	609.148090	C27H29O16	609.146108	4.9	609, 449, 301, 300, 223, 153	215, 261, 351	quercetin 3-O-rhamnoglucoside (rutin)	0.94
11	6.5	417.178248	C17H17N14	417.176610	15.9	417, 339, 179	not detected	unidentified	1.53

12	6.8	515.120987	C25H23O12	515.119500	7.1	515, 353, 191	219, 327	3,5-O- dicaffeoylguinic acid	0.26
13	7.3	561.162721	C27H29O13	561.161365	3.3	561, 357, 325, 191	219, 323	feruloylquinic acid derivative	3.18
14	7.8	373.188314	C18H29O8	373.186791	5.0	373, 211, 193	not detected	unidentified	0.26
15	8.0	417.178175	C19H29O10	417.176621	12.0	417, 373, 209, 193, 175, 134	not detected	unidentified	0.29
16	8.4	669.278406	C32H45O15	669.276394	15.5	669 (537+132pentose), 537 (375+162hexose) , 375, 300, 149	219, 327	unidentified	0.75
17	8.8	583.240673	C28H39O13	583.239615	3.1	583 (537+46HCOOH), 537 (375+162hexose) , 375, 165	199, 219	unidentified	0.33
18	9.1	415.198103	C20H31O9	415.197356	2.2	415, 373, 211, 193	not detected	unidentified	0.33
19	9.5	193.050825	C10H9O4	193.050632	4.2	385, 313, 193	219, 327	ferulic acid derivative	0.46

20	9.6	579.245520	C29H39O12	579.244700	8.3	579, 435, 375, 193	219	unidentified	0.41
21	9.9	729.296637	C34H49O17	729.297524	14.4	729(683+46HCOOH), 683(551+132pentose), 551(389+162hexose), 389, 233, 191, 149	223	unidentified	1.45
22	10.4	597.255129	C29H41O13	597.255265	5.8	597 (551+46HCOOH), 551(389+162hexose), 389, 341, 161, 113	223	unidentified	1.19
23	11.1	593.259308	C30H41O12	593.260350	8.7	593(547+46HCOOH), 547, 457, 389, 265, 161	223	unidentified	0.40
24	11.7	577.133821	C30H25O12	577.135150	3.1	577, 385, 282, 193	219, 323	catechin derivative	1.19
25	12.1	845.321517	C42H53O18	845.323738	14.1	845, 693, 583, 499, 433, 221	not detected	unidentified	-
26	12.4	397.186333	C20H29O8	397.186791	6.5	397, 355, 265, 211	not detected	unidentified	-

27	12.6	609.276356	C27H45O15	609.276394	6.3	609, 563, 439, 355, 265, 161	not detected	unidentified	-
28	12.8	429.212614	C21H33O9	429.213006	12.0	429, 387, 295, 193,161	not detected	unidentified	-
29	13.0	577.135116	C30H25O12	577.135150	8.4	577, 447, 385, 265, 193	223	(+) - procyanidin B2 (Epi catechin dimer)	0.83
30	13.2	577.134609	C30H25O12	577.135150	6.7	577, 533, 341, 282, 163	223	(+) - procyanidin B2 (Epi catechin dimer)-isomer	0.72
31	14.0	581.270797	C55H87O26	581.270915	45.3	1117, 731, 581, 523, 455, 373	not detected	oleanane-type triterpenoid	0.27
32	14.4	443.229625	C22H35O9	443.228656	14.4	443, 404, 337, 309	not detected	unidentified	-
33	15.3	955.489997	C48H75O19	955.490804	35.2	955, 776, 613, 569, 500 (-2), 309	not detected	medicagenic acid- type triterpenoid ?	0.37
34	17.9	955.4901	traces			955, 525, 449, 309, 146	not detected	oleanane-type triterpenoid (Polyscioside C/ Polyscioside A)	0.71
35	19.0	265.148897	C15H21O4	265.144533	22.6	265	not detected	unidentified	-

36	19.8	793.438339	C42H65O14	793.437980	17.3	793, 613, 531, 509, 146	not detected	spinasaponin A/ zingibroside R1 (oleanane-type triterpenoid)	0.38
37	21.9	723.381495	C34H59O16	723.380859	13.8	723, 507, 397, 305, 146	not detected	unidentified	-
38	22.9	433.236792	C28H33O4	433.238433	44.6	433, 187	not detected	unidentified	-
39	26.0	515.323180	C27H47O9	515.322557	11.3	515, 437, 279, 161	not detected	octadecanoic acid derivative	0.46
40	26.7	491.323248	C25H47O9	491.322557	3.7	491, 375, 297, 139	not detected	palmitate acid derivative	1.20

a)

L-malic acid RT=0.7

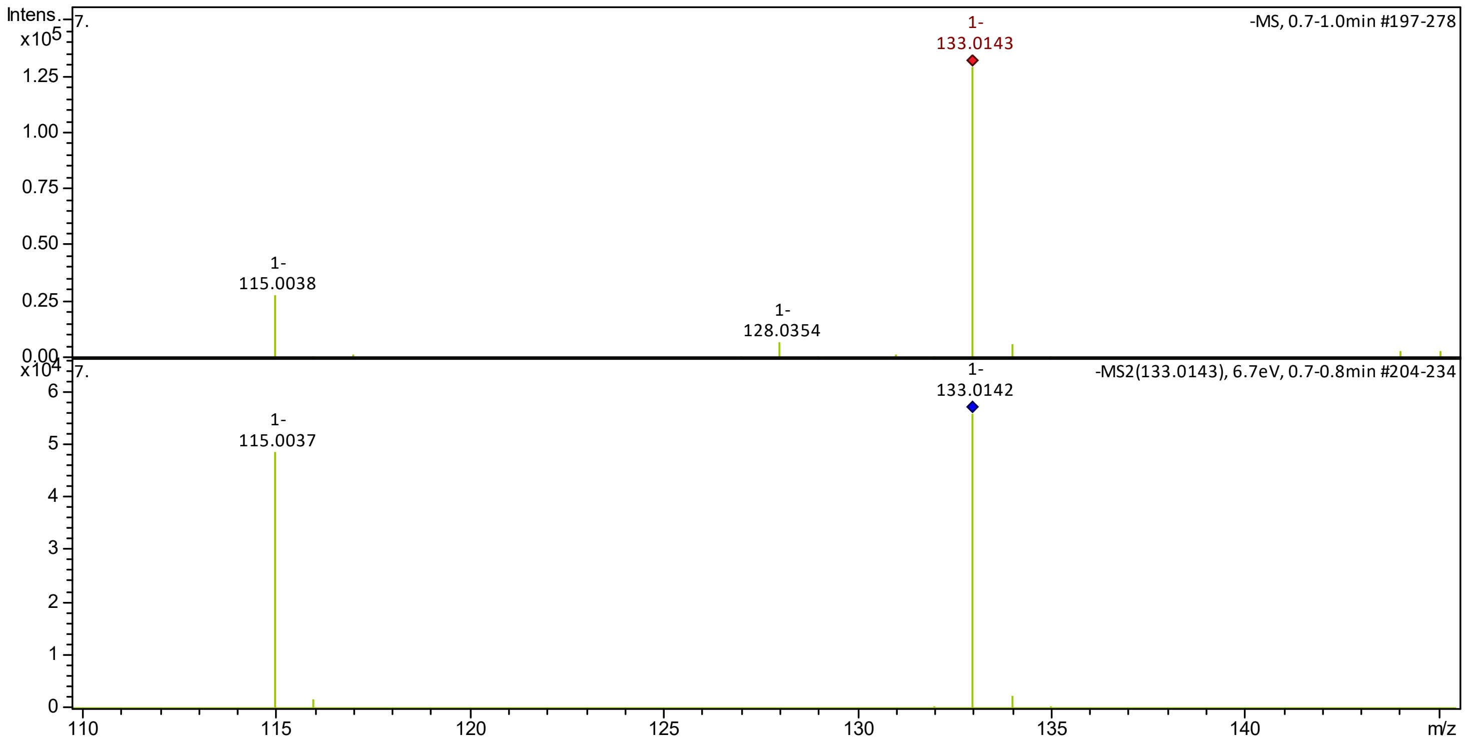

Intens.
x10^5
1.25
1.00
0.75
0.50
0.25
0.00
-MS, 0.7-1.0min #197-278
1-
133.0143
1-
115.0038
1-
128.0354
x10^4
-MS2(133.0143), 6.7eV, 0.7-0.8min #204-234
1-
133.0142
1-
115.0037
110
115
120
125
130
135
140
m/z

4-oxoproline RT=0.9

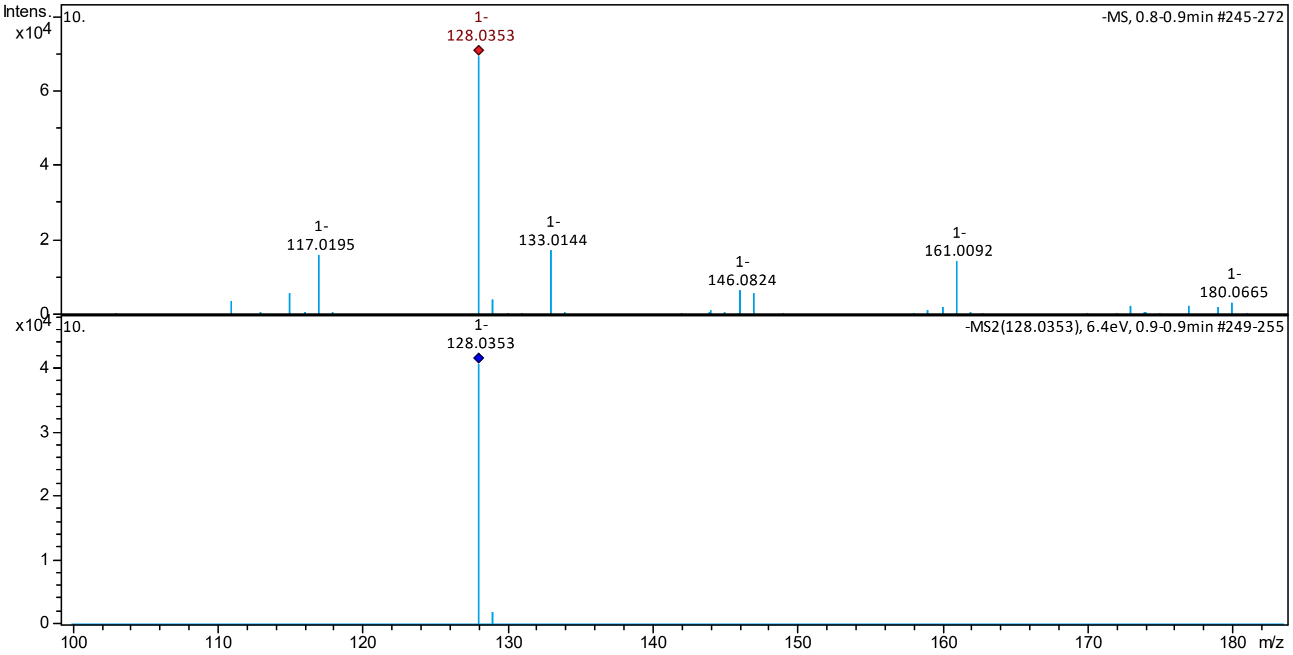

1/3/5-O-caffeoylguinic acid RT=1.5

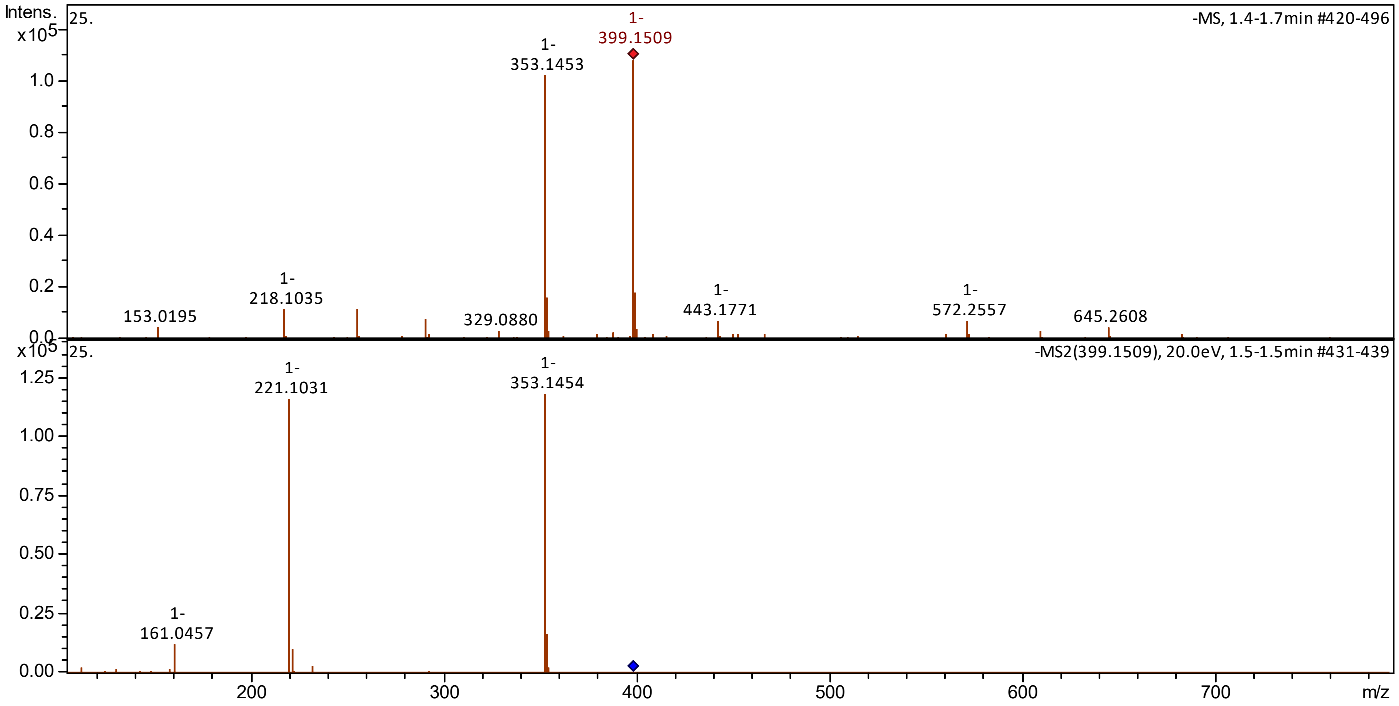

L-tryptophan RT=1.9

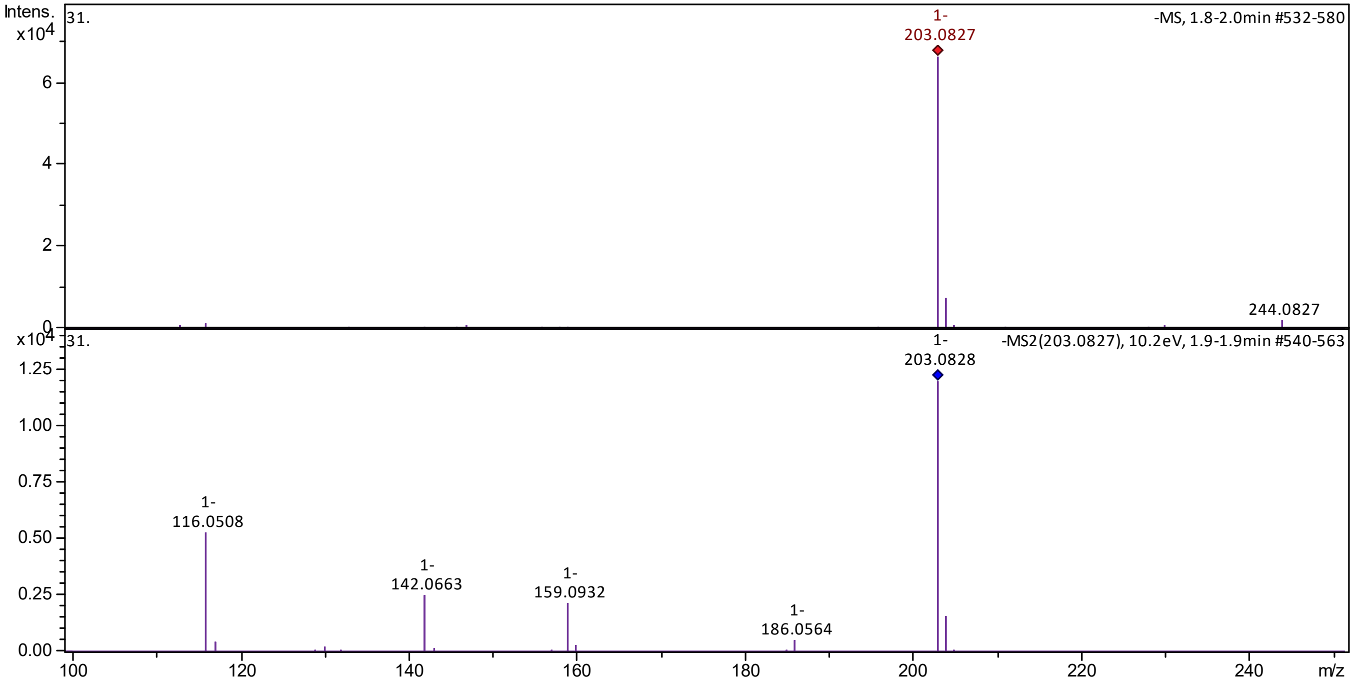

1/3/5-O-caffeoylguinic acid RT=2.7

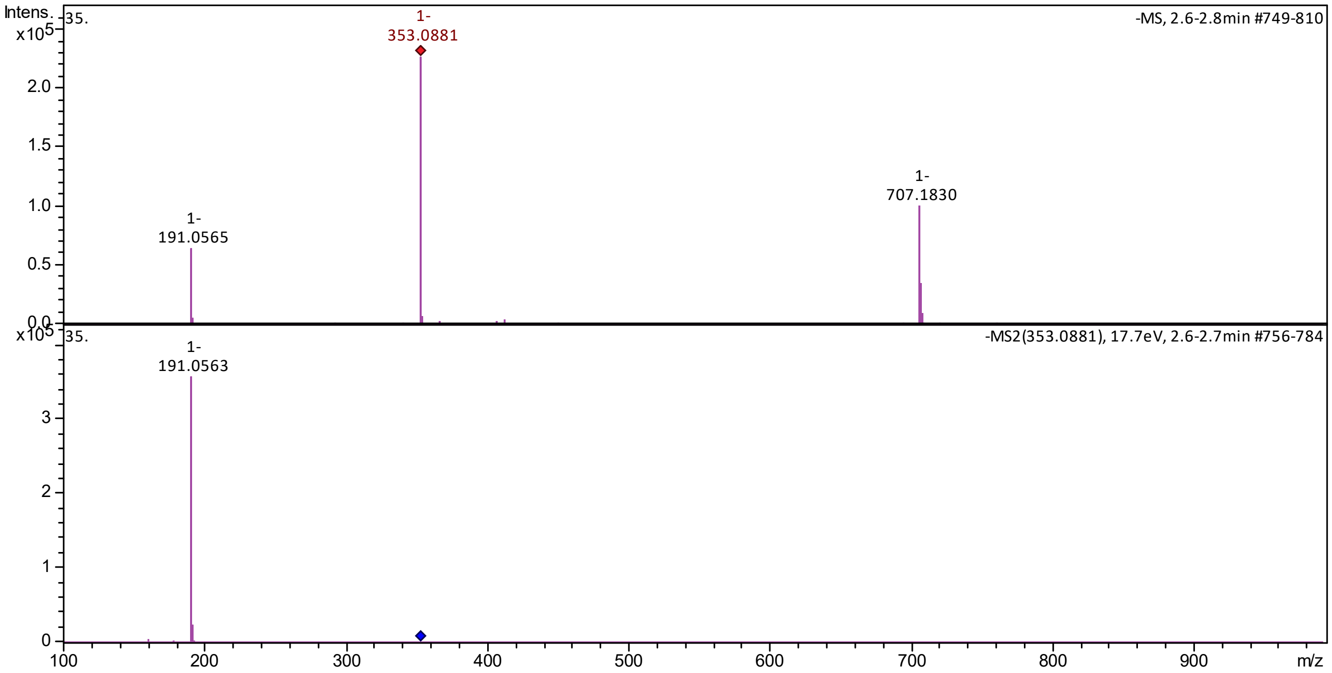

1/3/5-O-caffeoylguinic acid RT=2.9

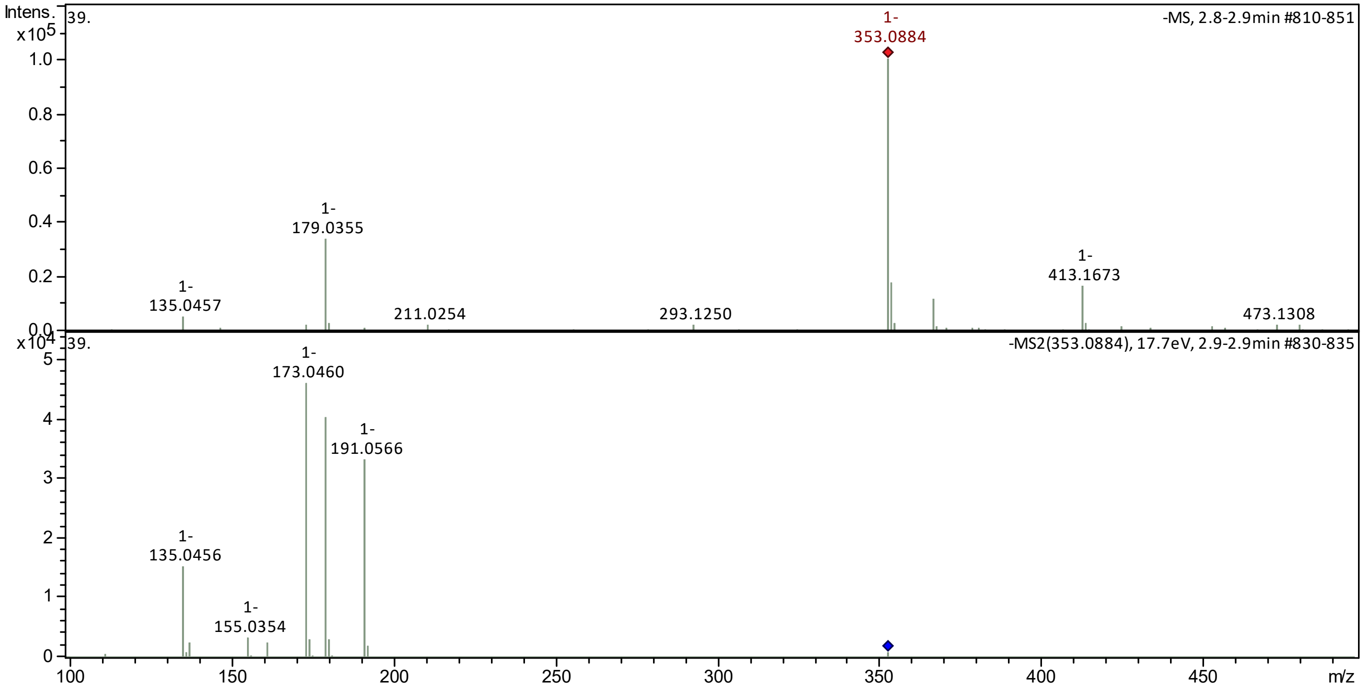

8-O-acetyl shanzhiside methyl ester   RT=3.6

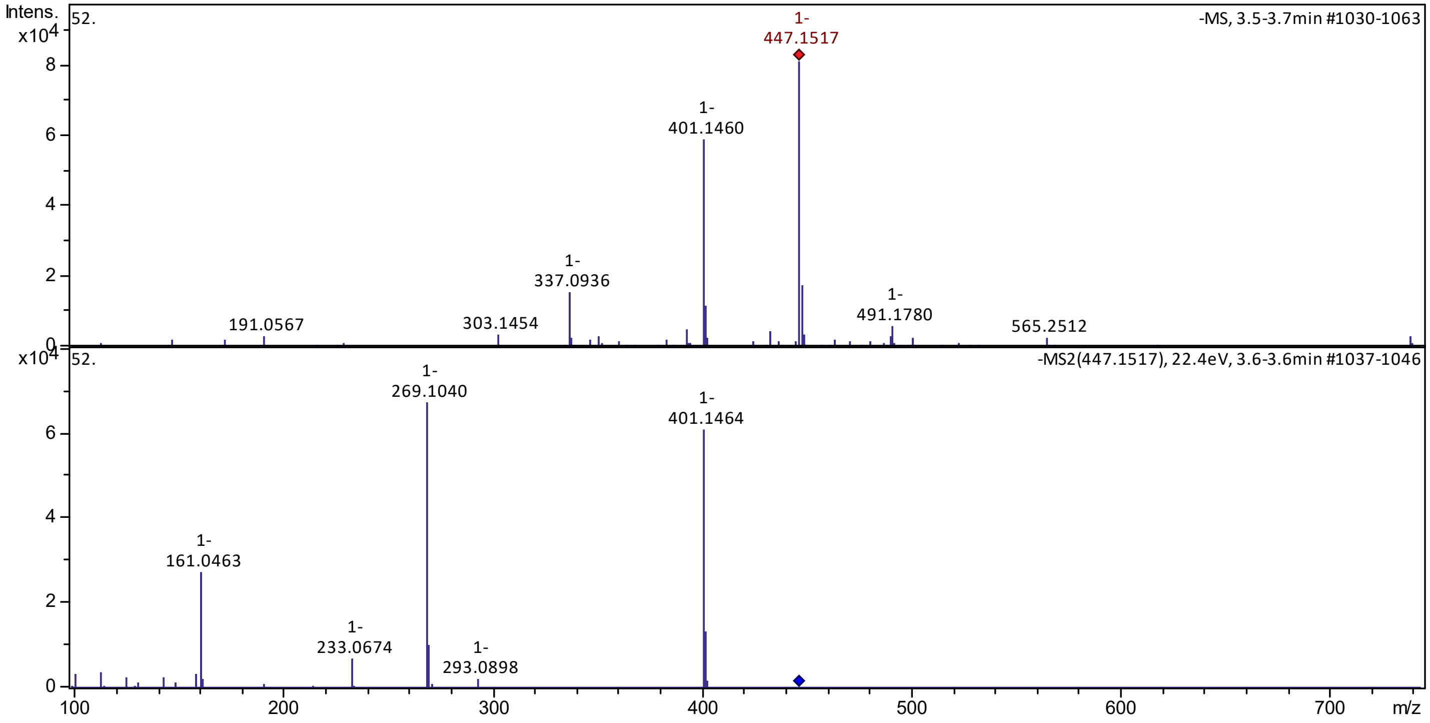

5-O-Feruloylquinic acid RT=4.4

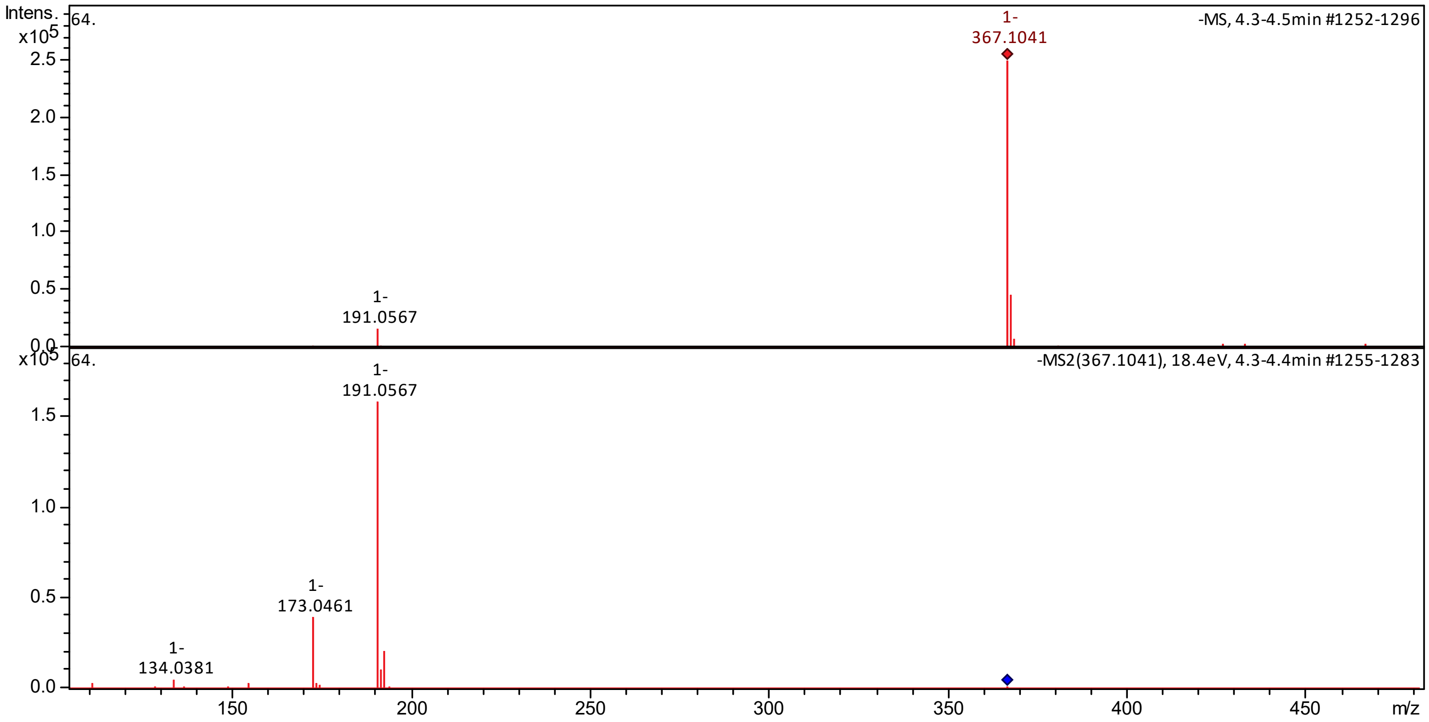

quinic acid derivative RT=5.2

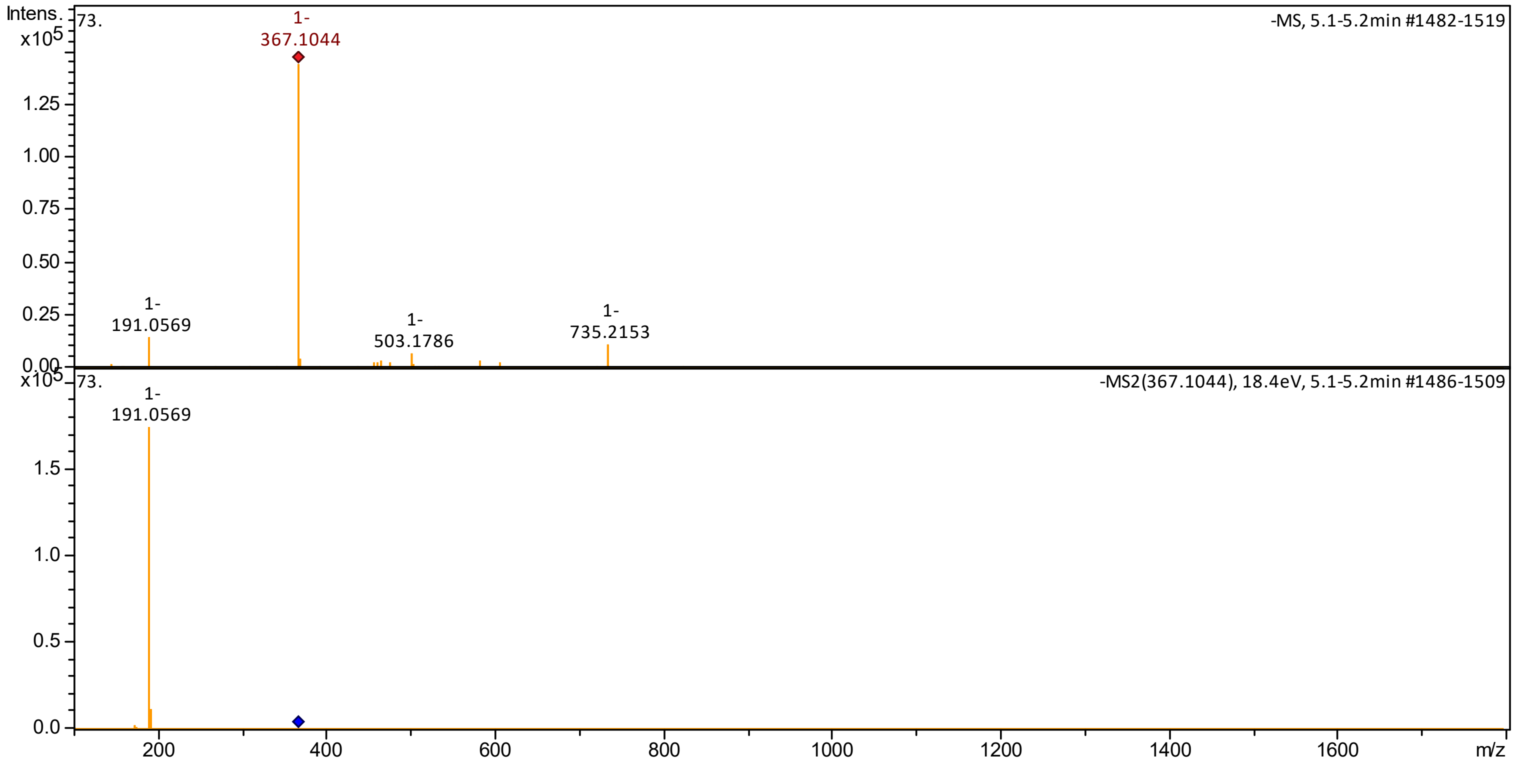

caffeic acid derivative RT=6.3

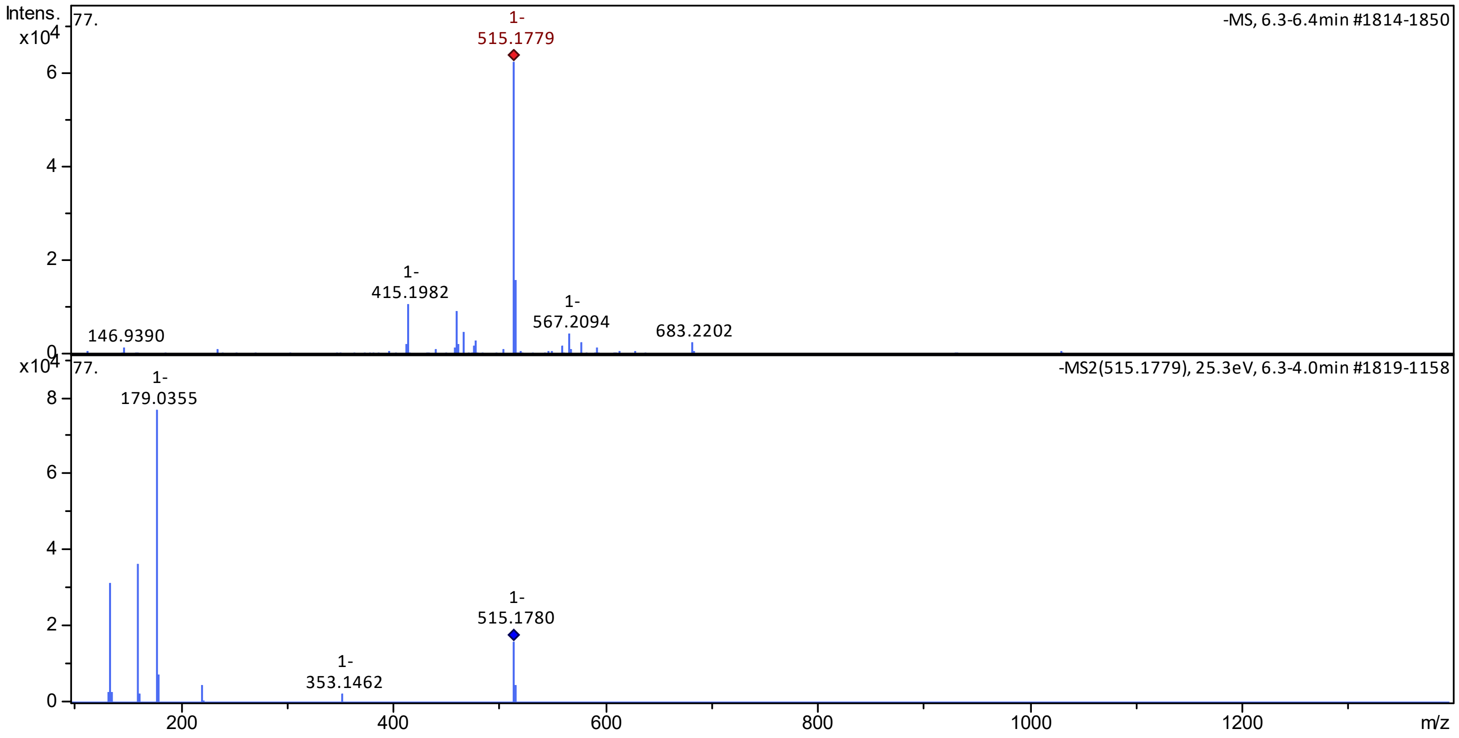

unidentified RT=6.9

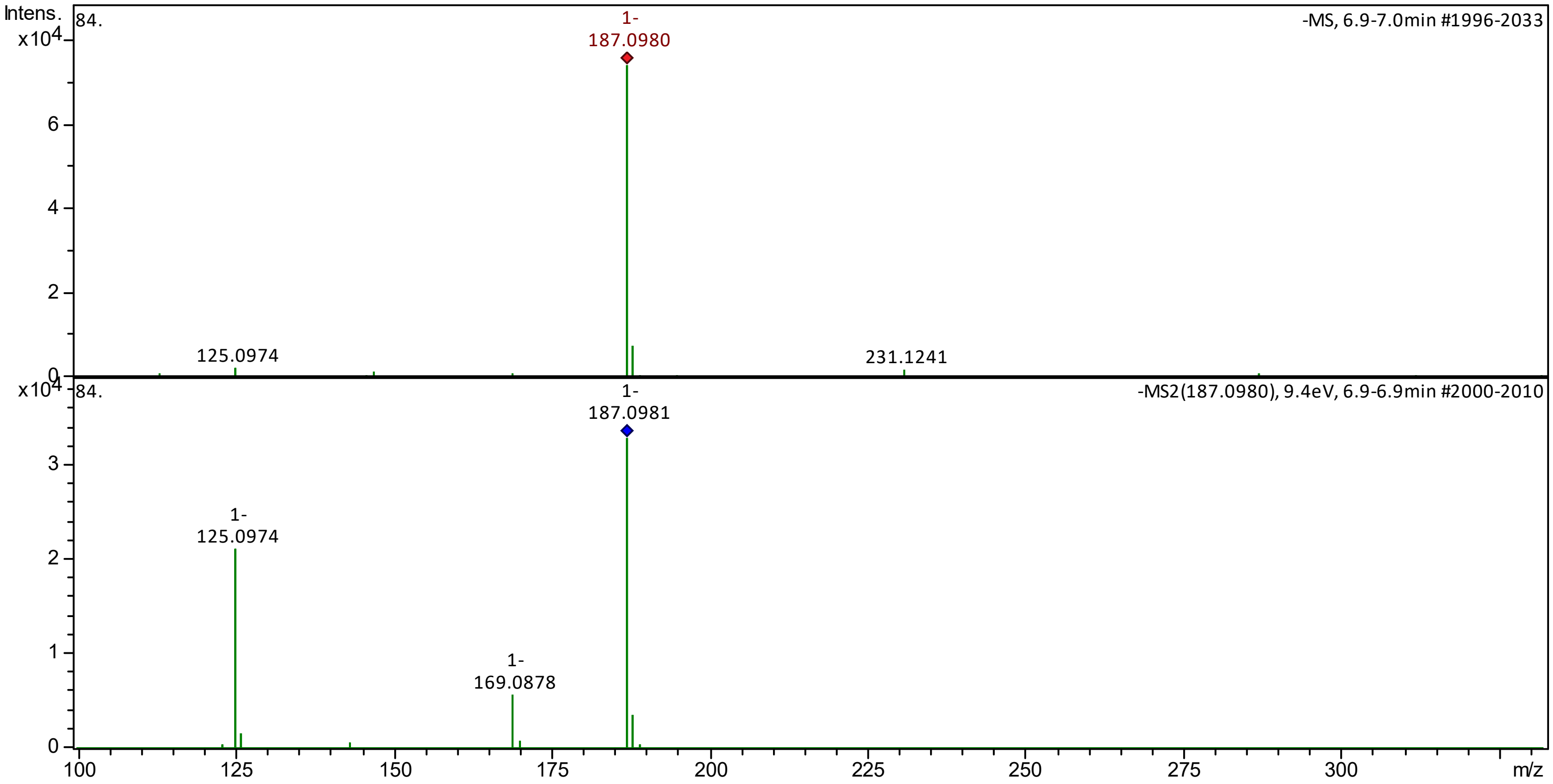

unidentified RT=10.4

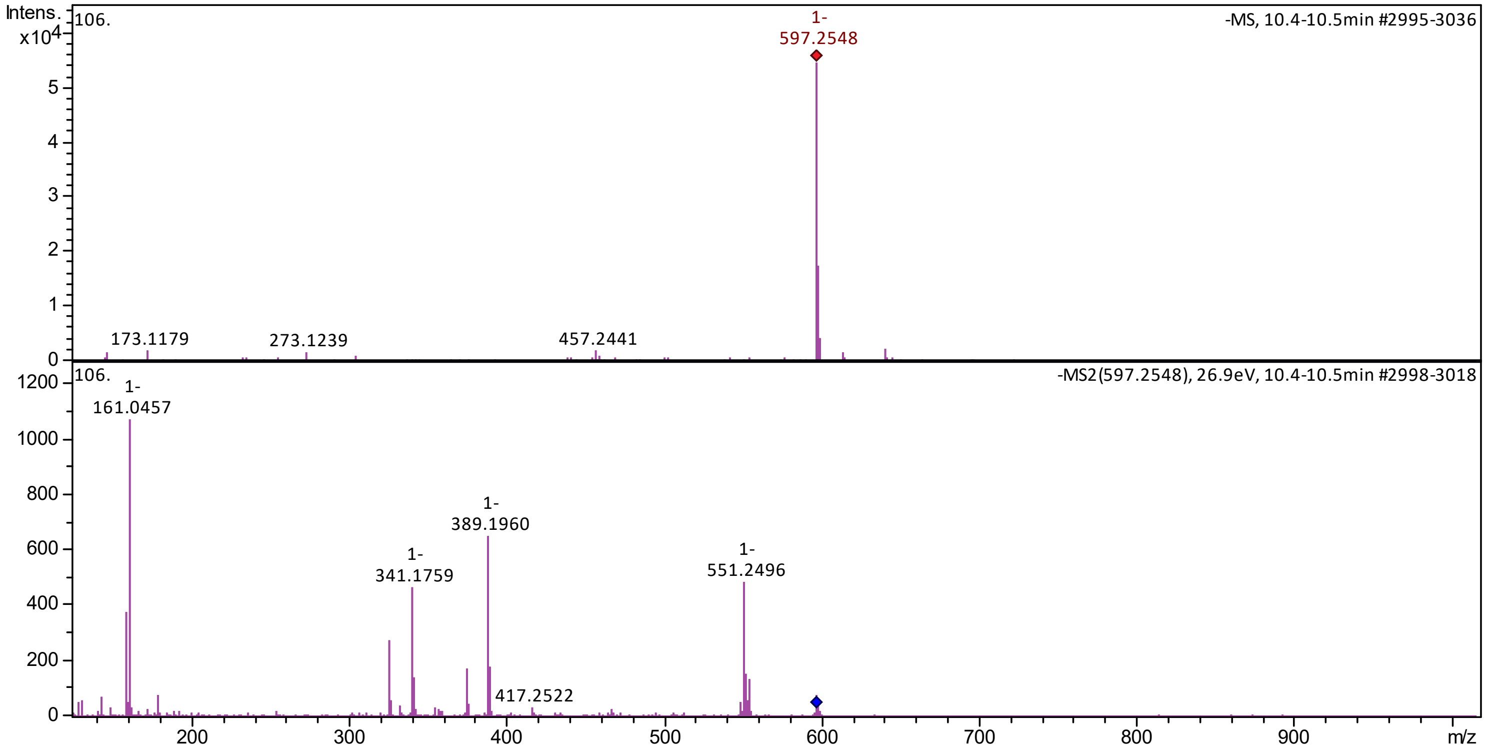

catechin derivative RT=11.7

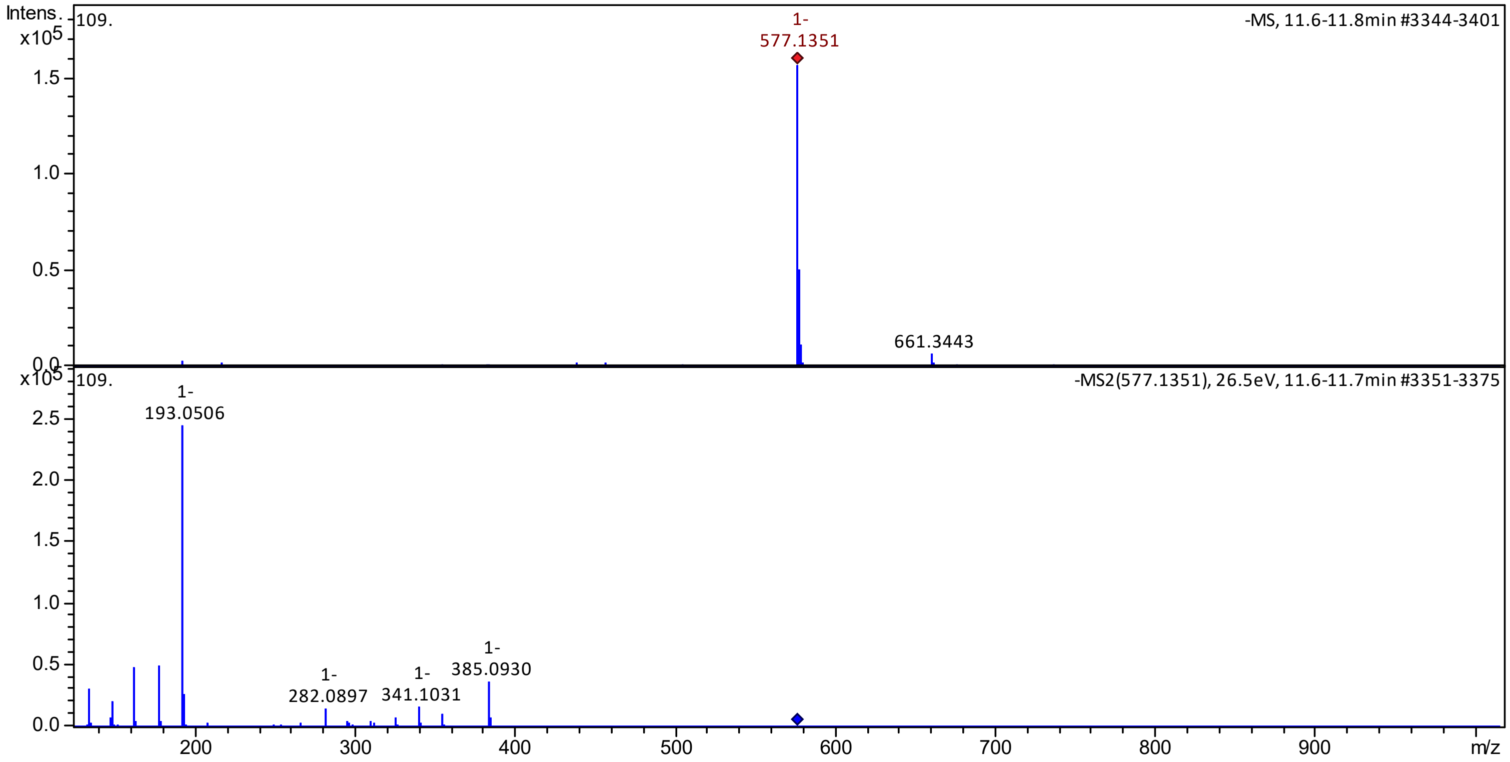

(+) - procyanidin B2 RT=13.3

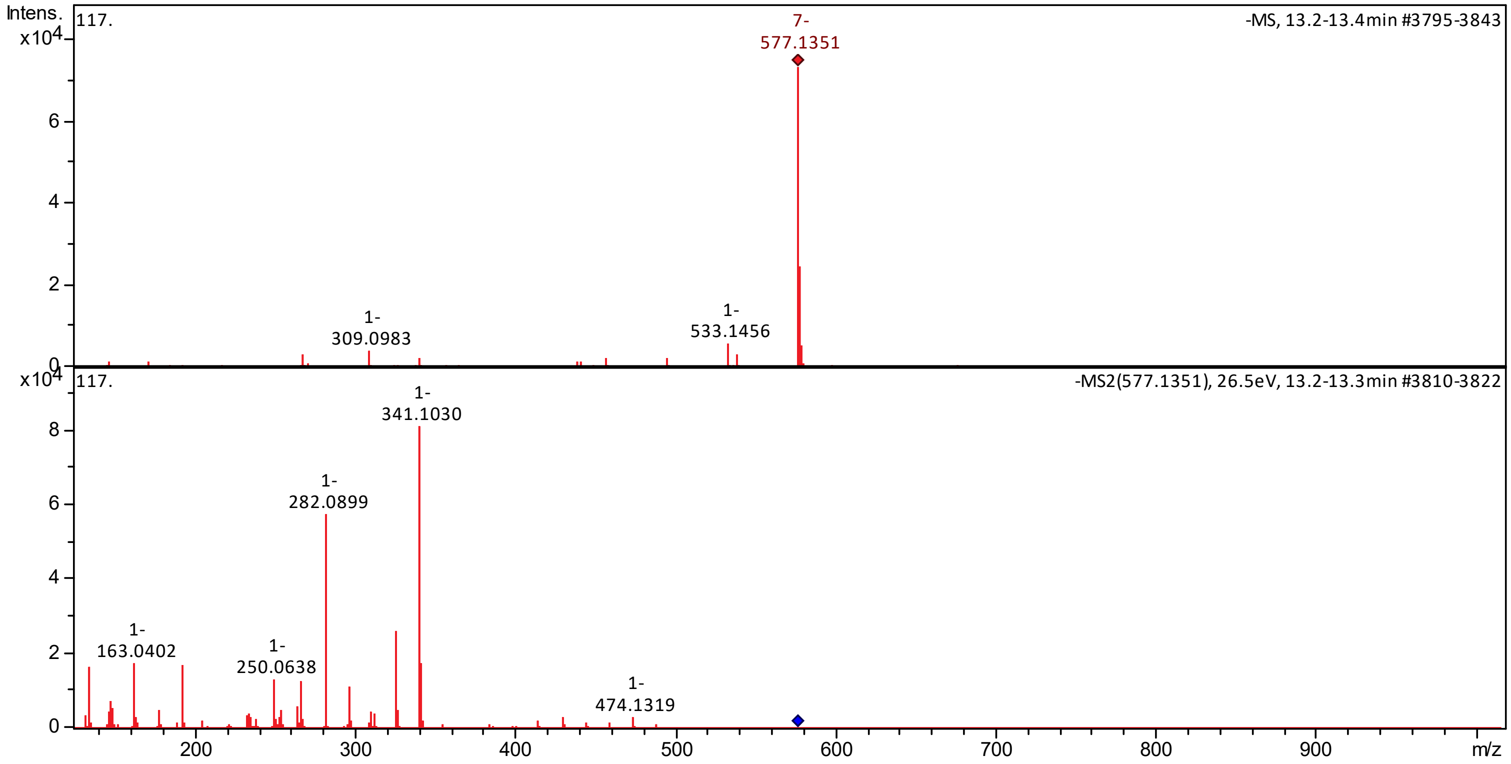

dammarenolic acid – type triterpenoid RT=13.7

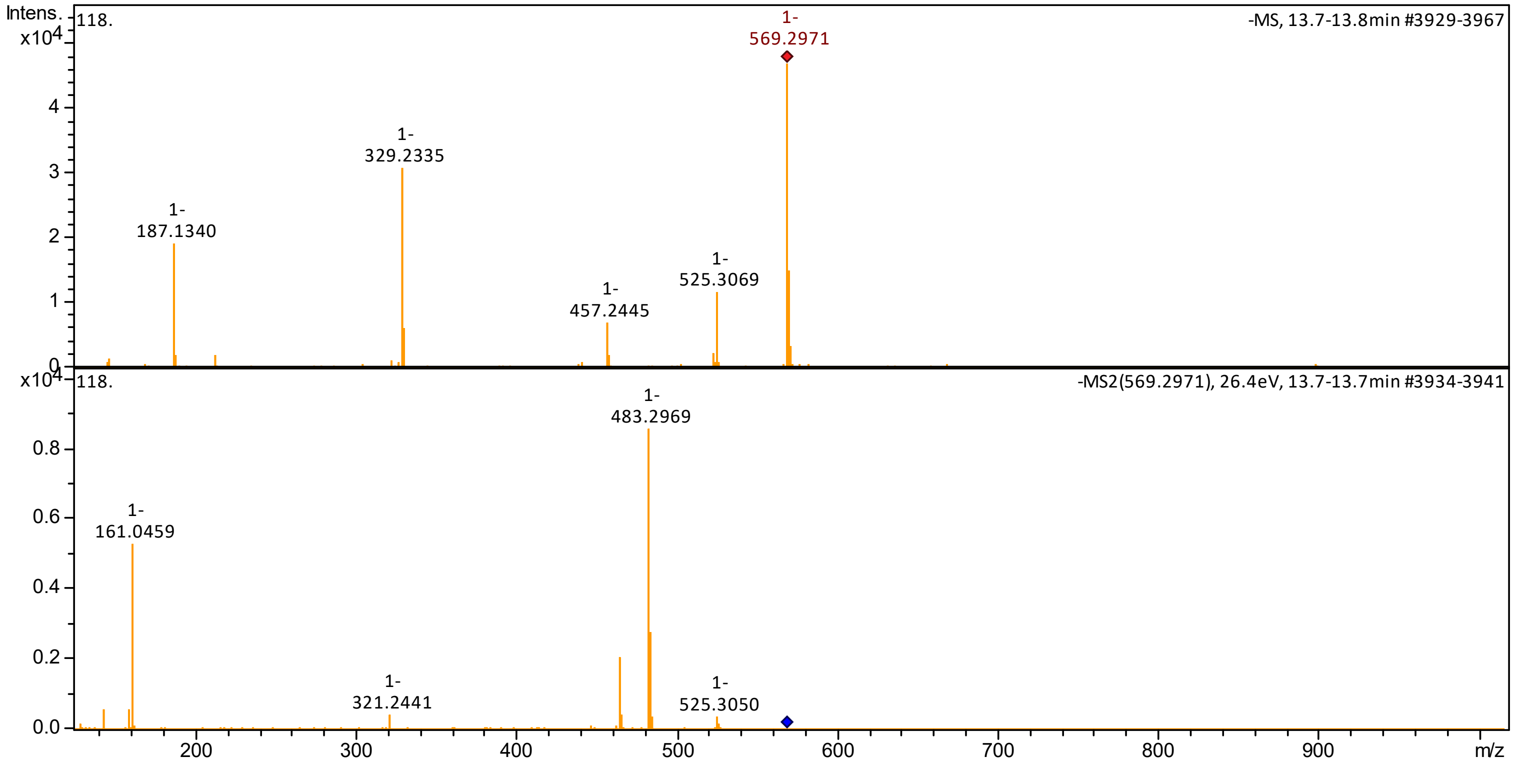

oleanane-type triterpenoid RT=14.1

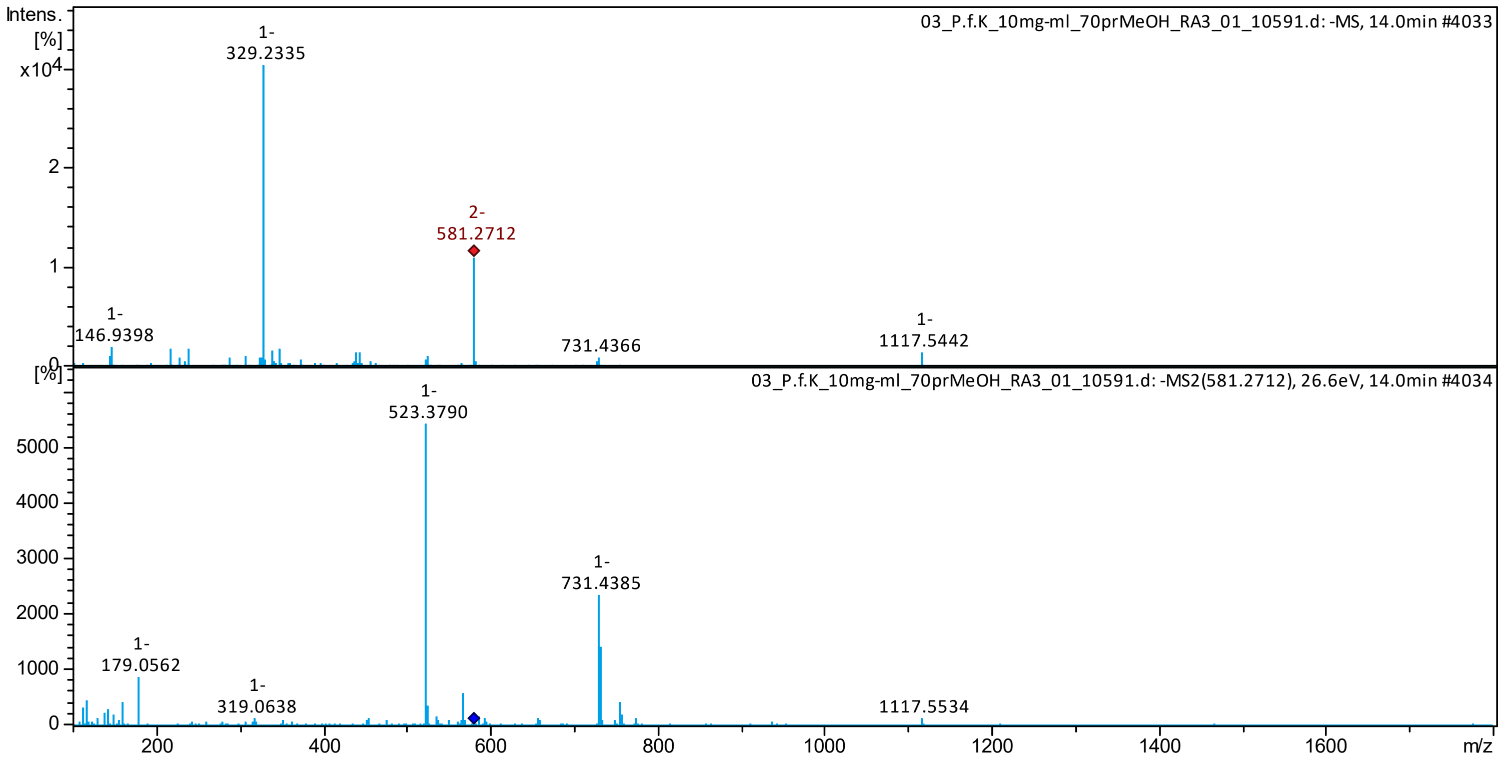

medicagenic acid- type triterpenoid RT=15.3

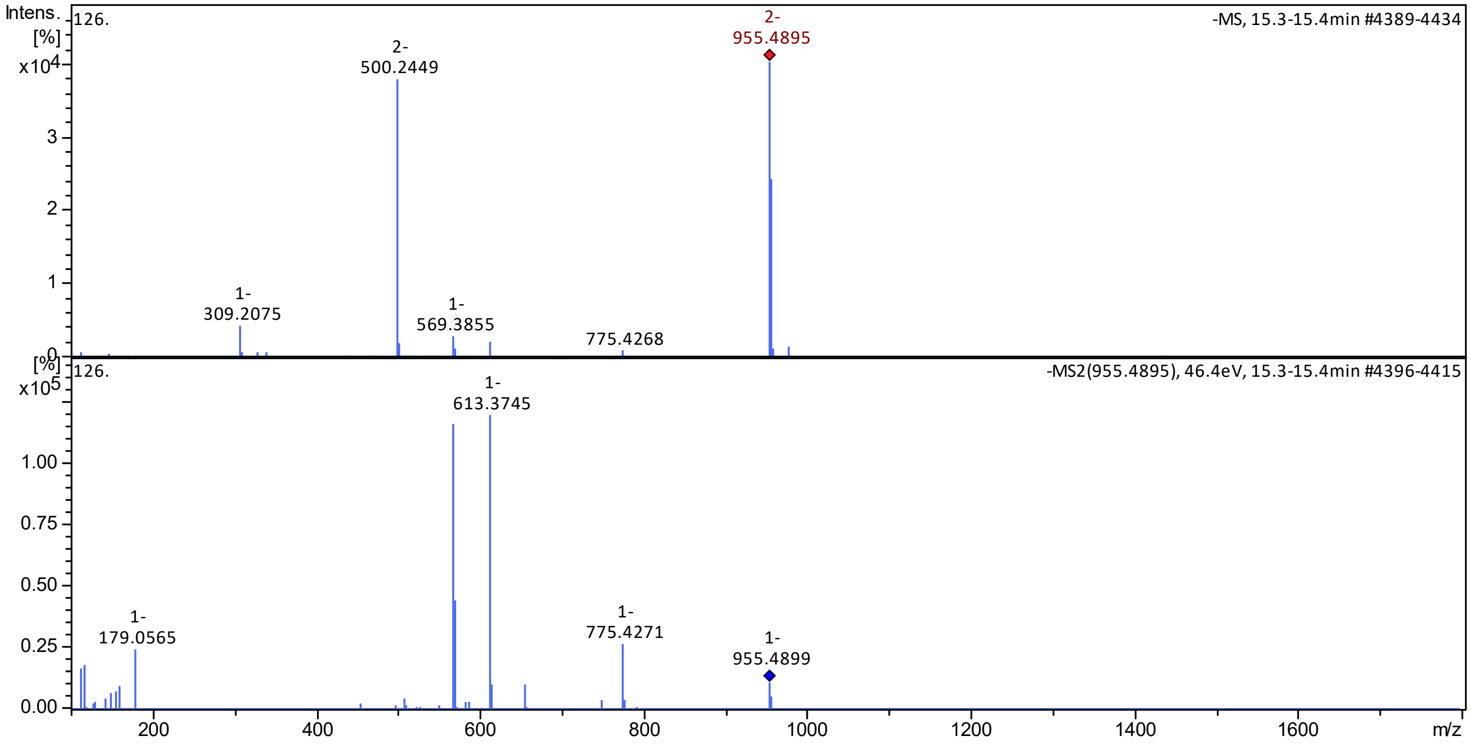

unidentified RT=17.1

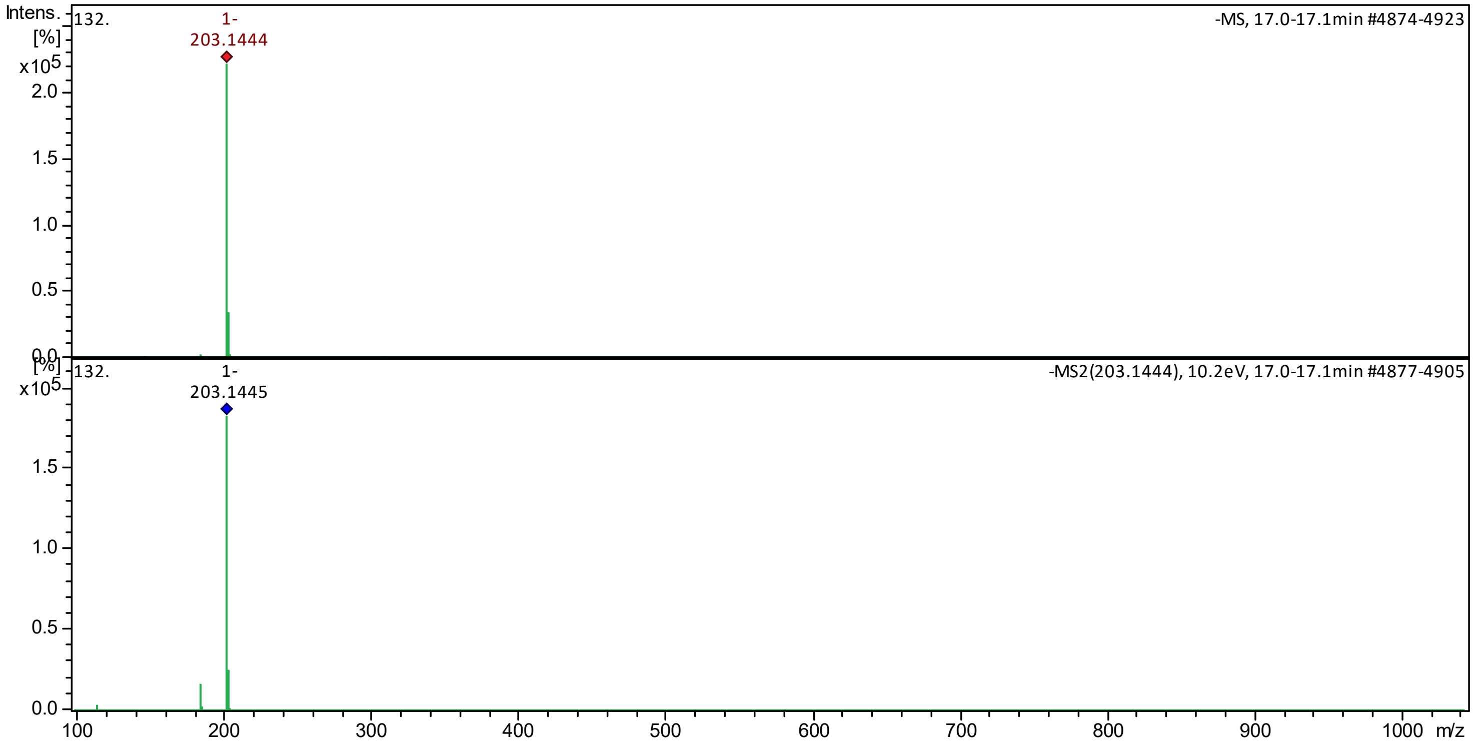

spinasaponin A/ zingibroside R1 (oleanane-type triterpenoid) RT=19.8

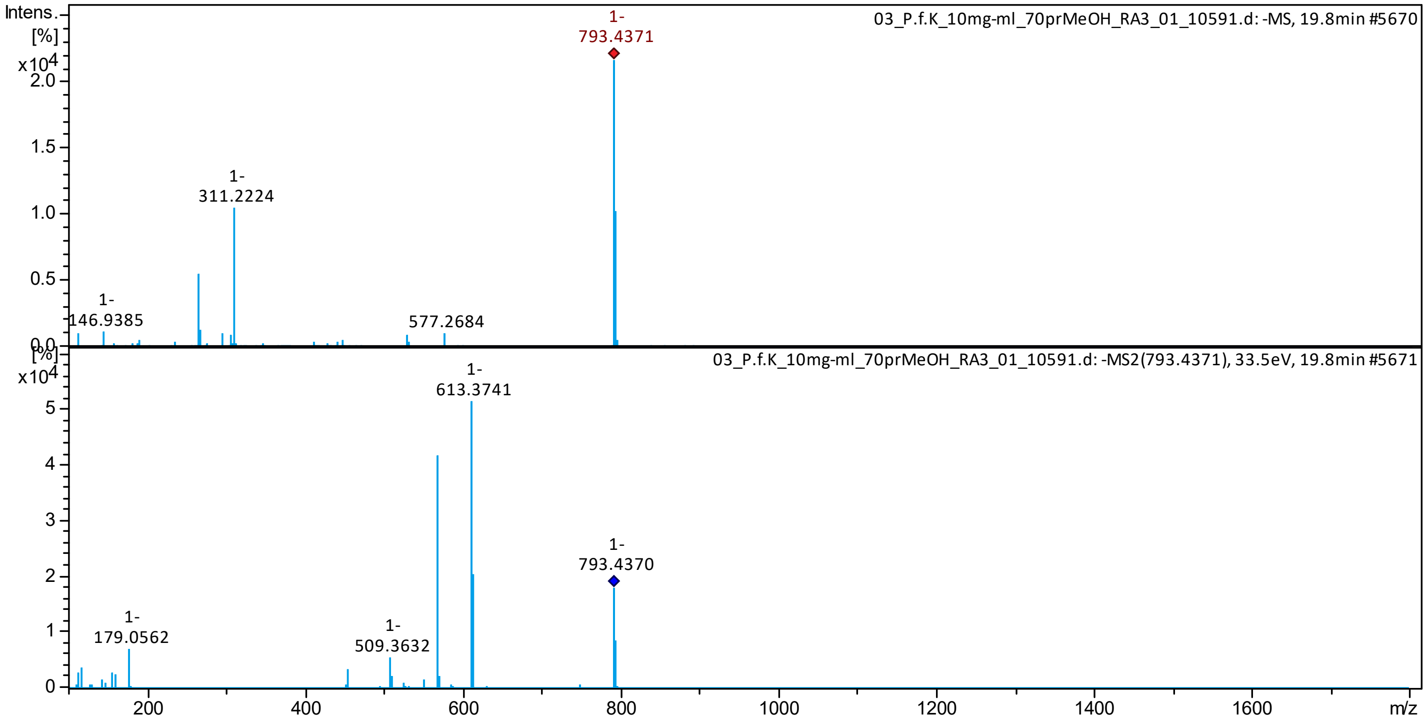

13-Hydroxy-6,9,11-octadecatrienoic acid   RT=20.8

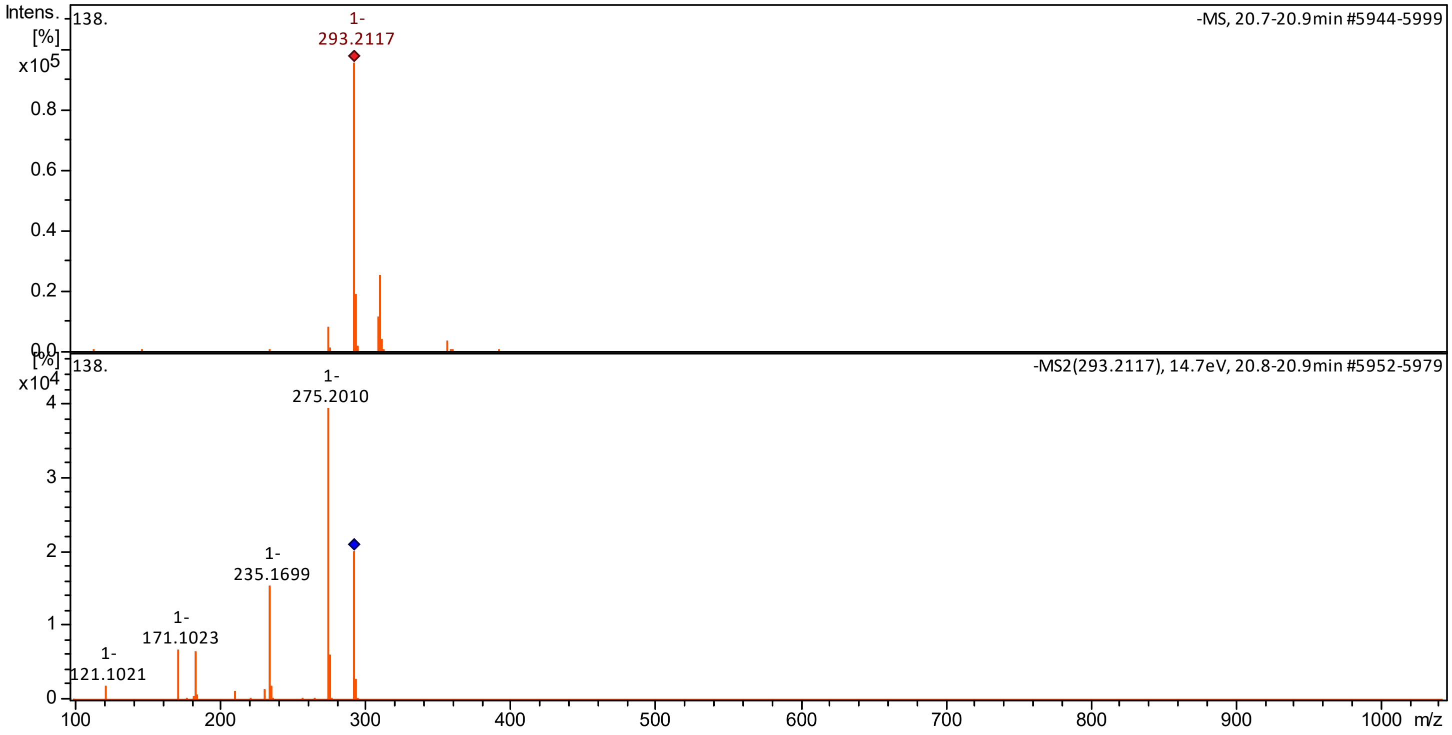

cis-12,13-Epoxy-cis-9-octadecenoic acid RT=22.2

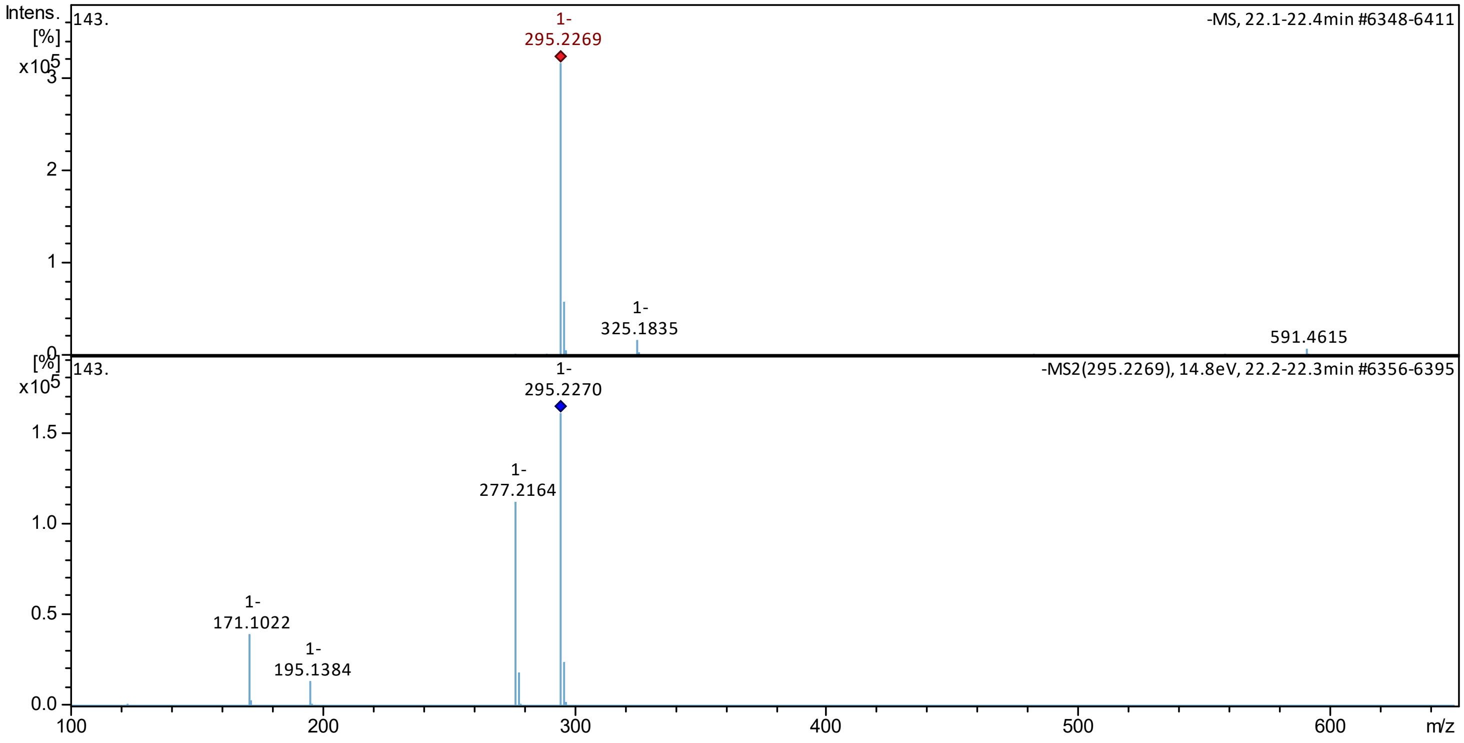

linoleic acid RT=26.9

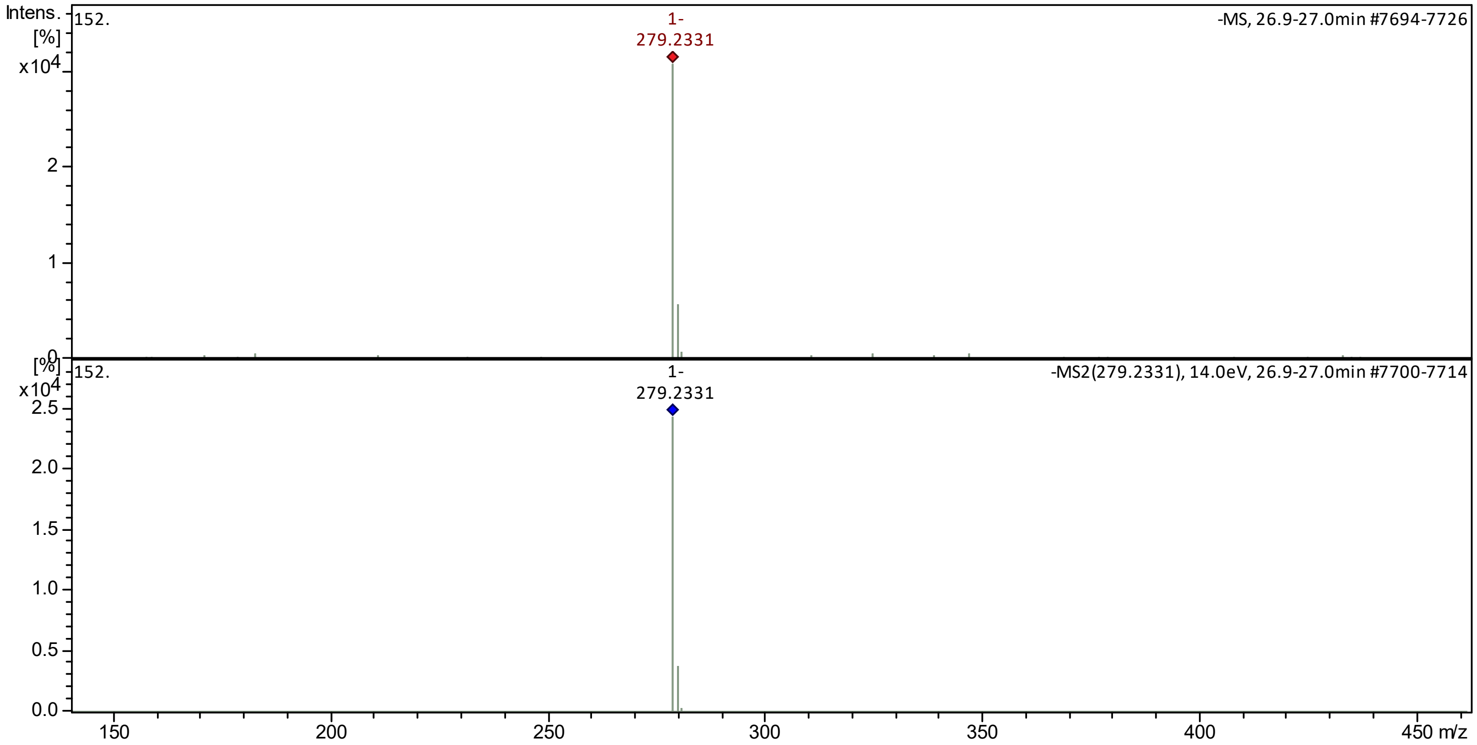

b)

L-malic acid RT=0.7

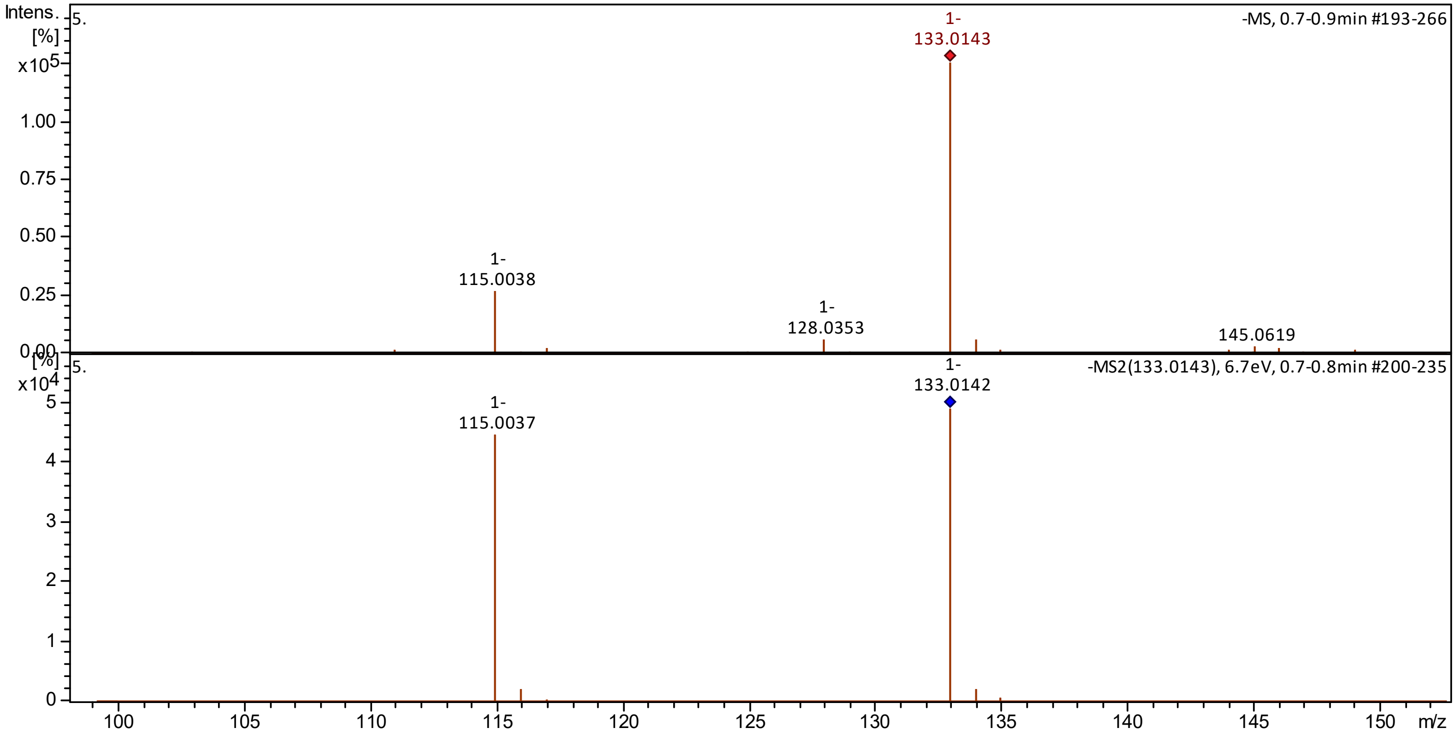

4-oxoproline RT=0.9

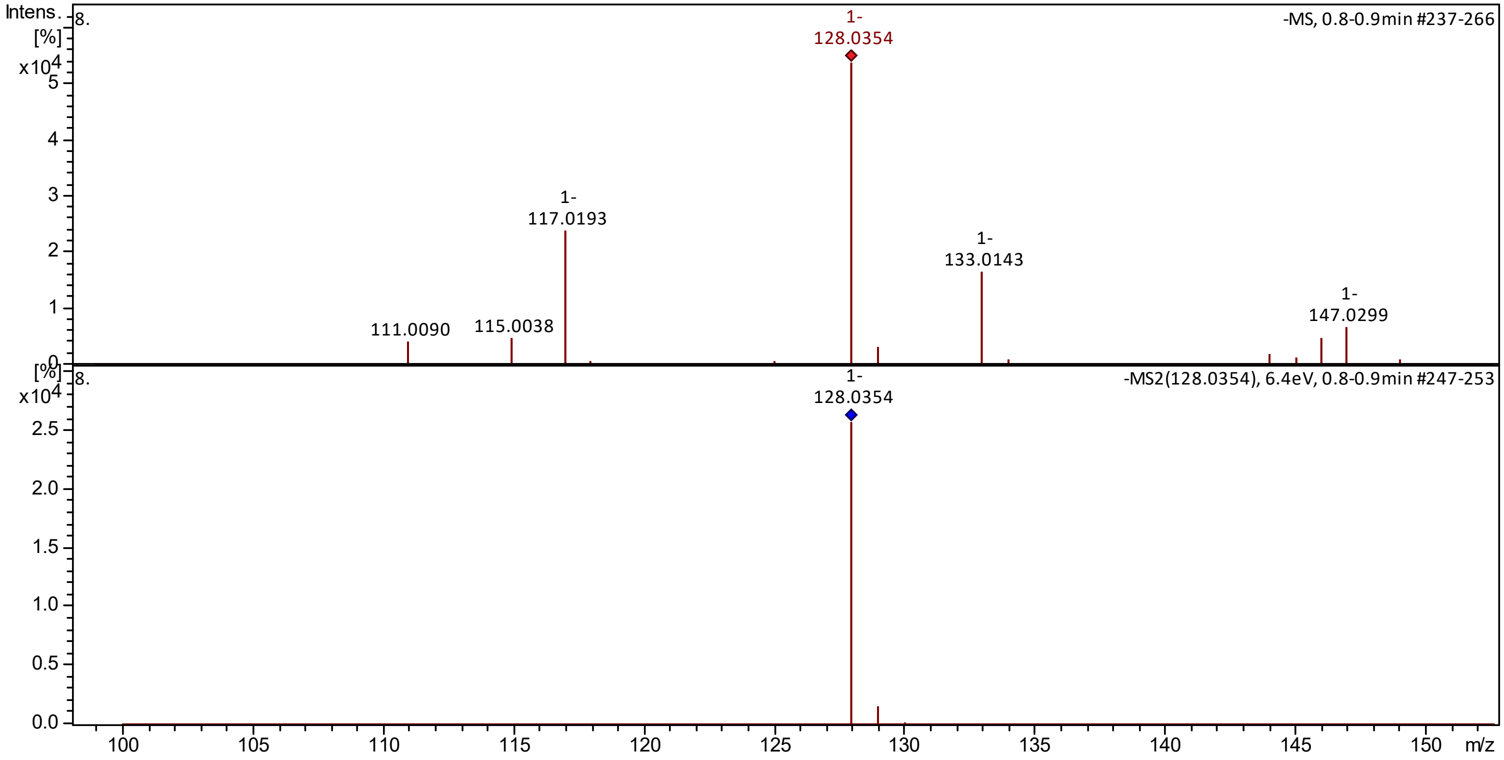

L-tryptophan RT=1.9

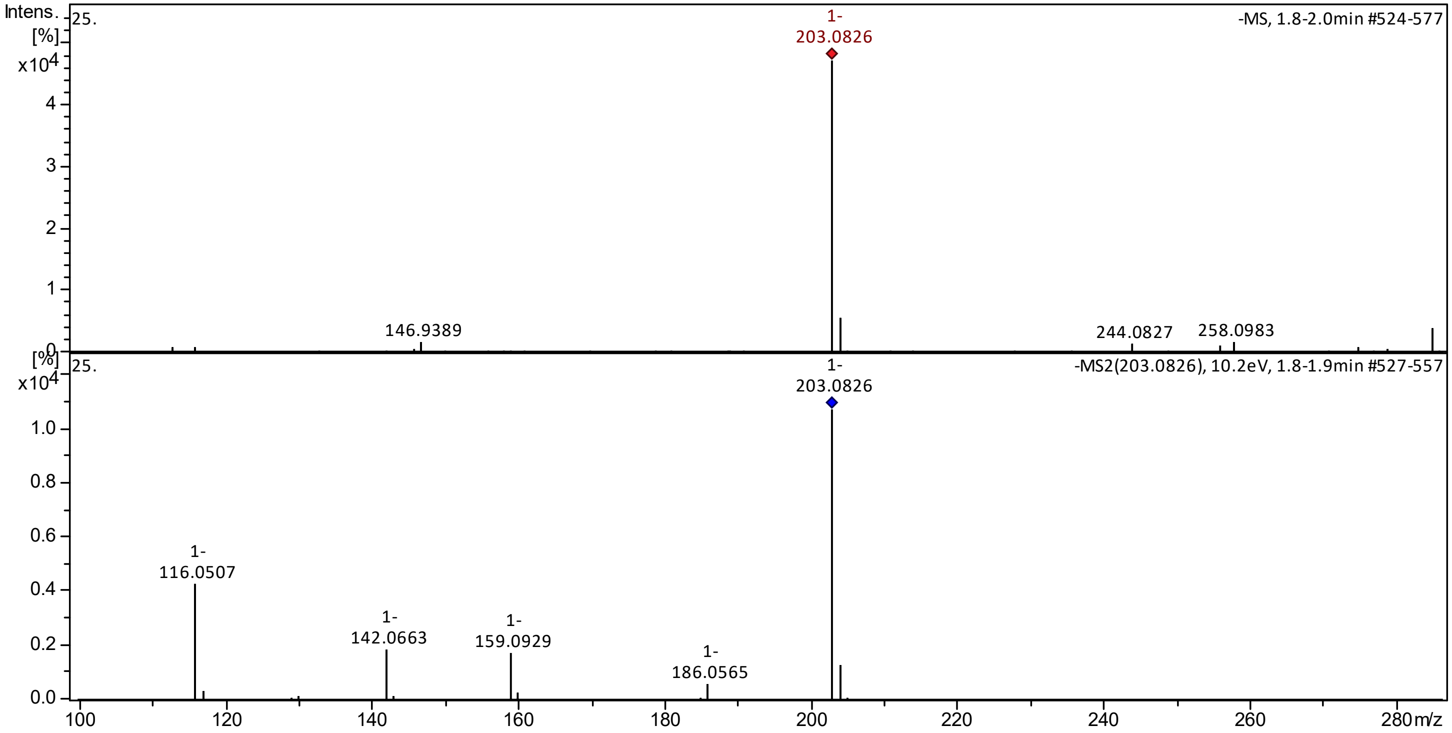

1/3/5-O-caffeoylguinic acid RT=2.7

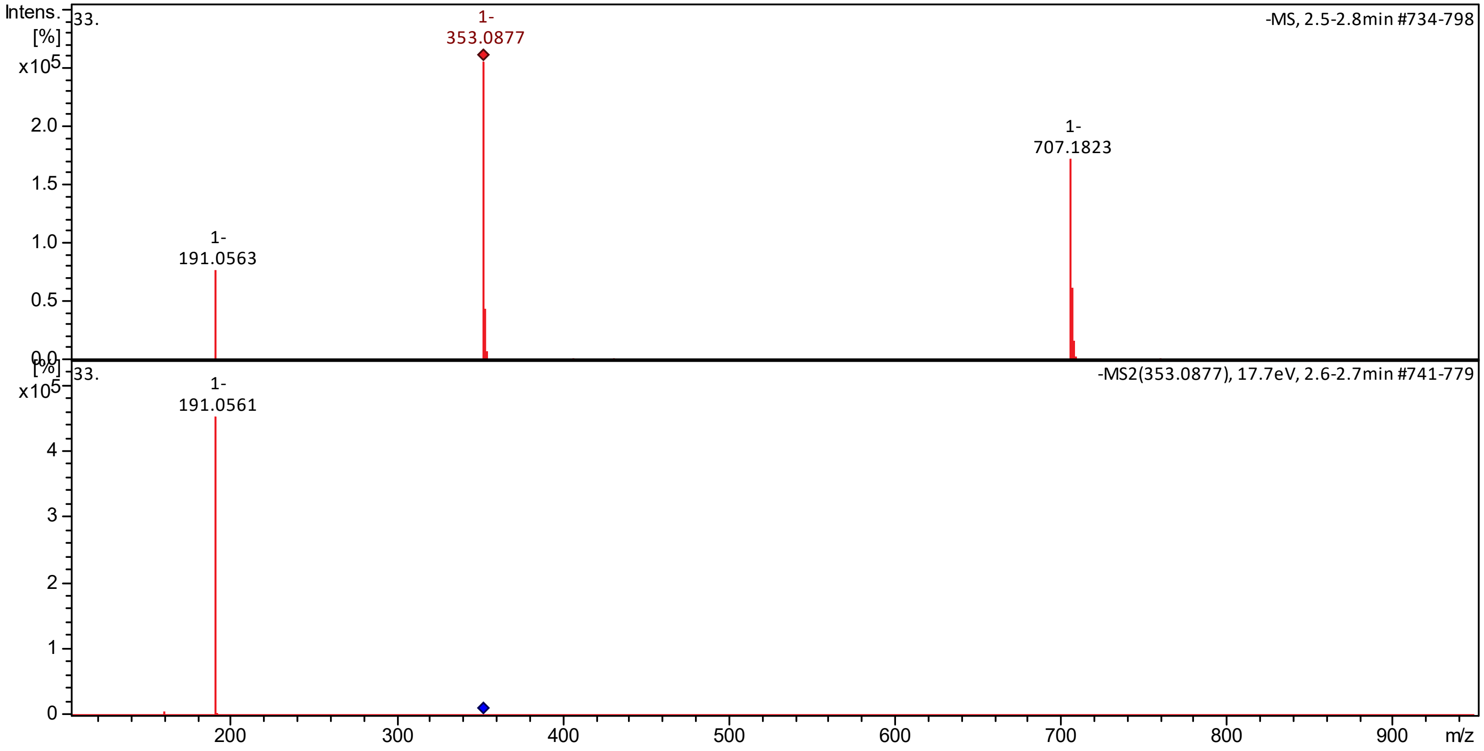

1/3/5-O-caffeoylguinic acid RT=2.8

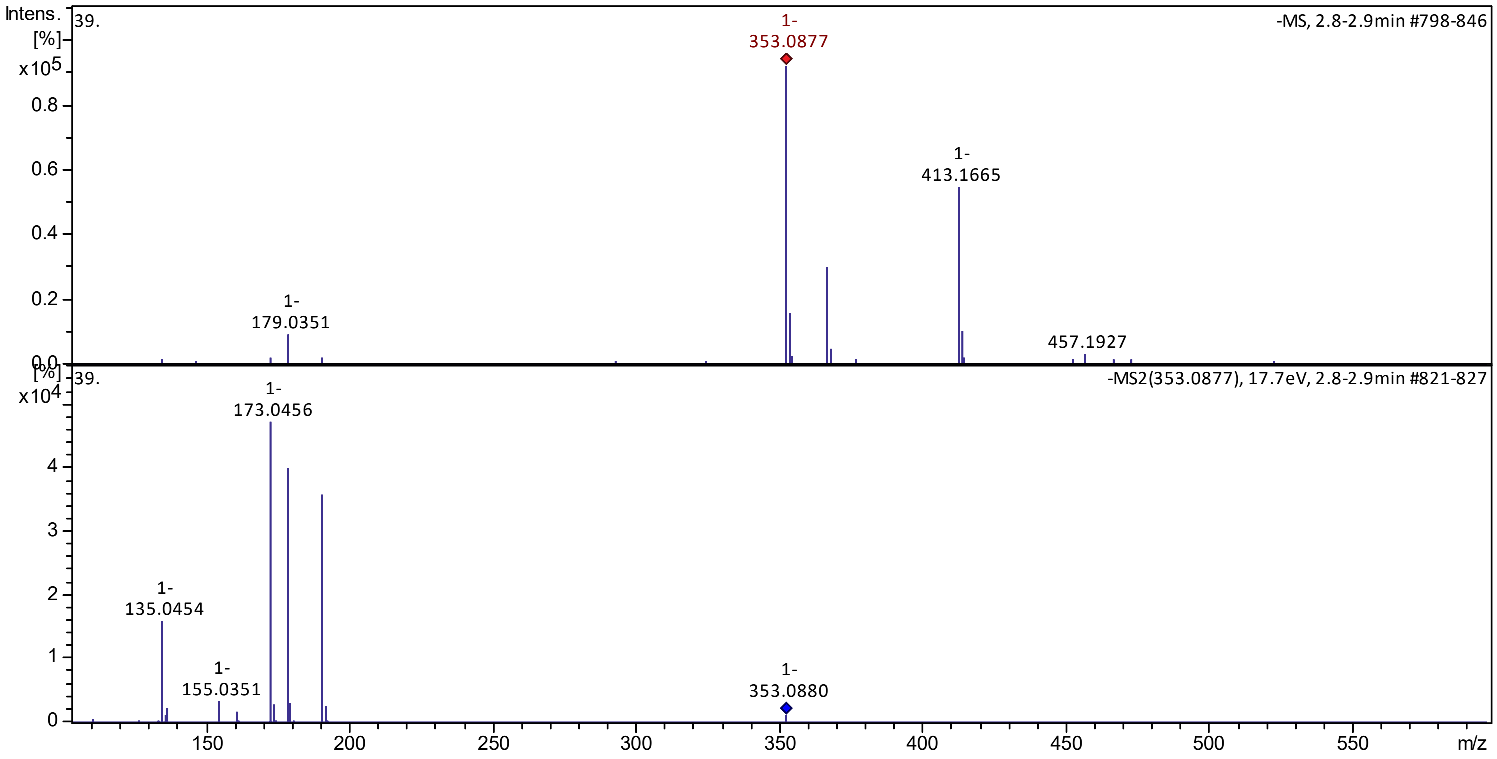

Caffeoyl-deoxyquinic acid (izomer)   RT=4.5

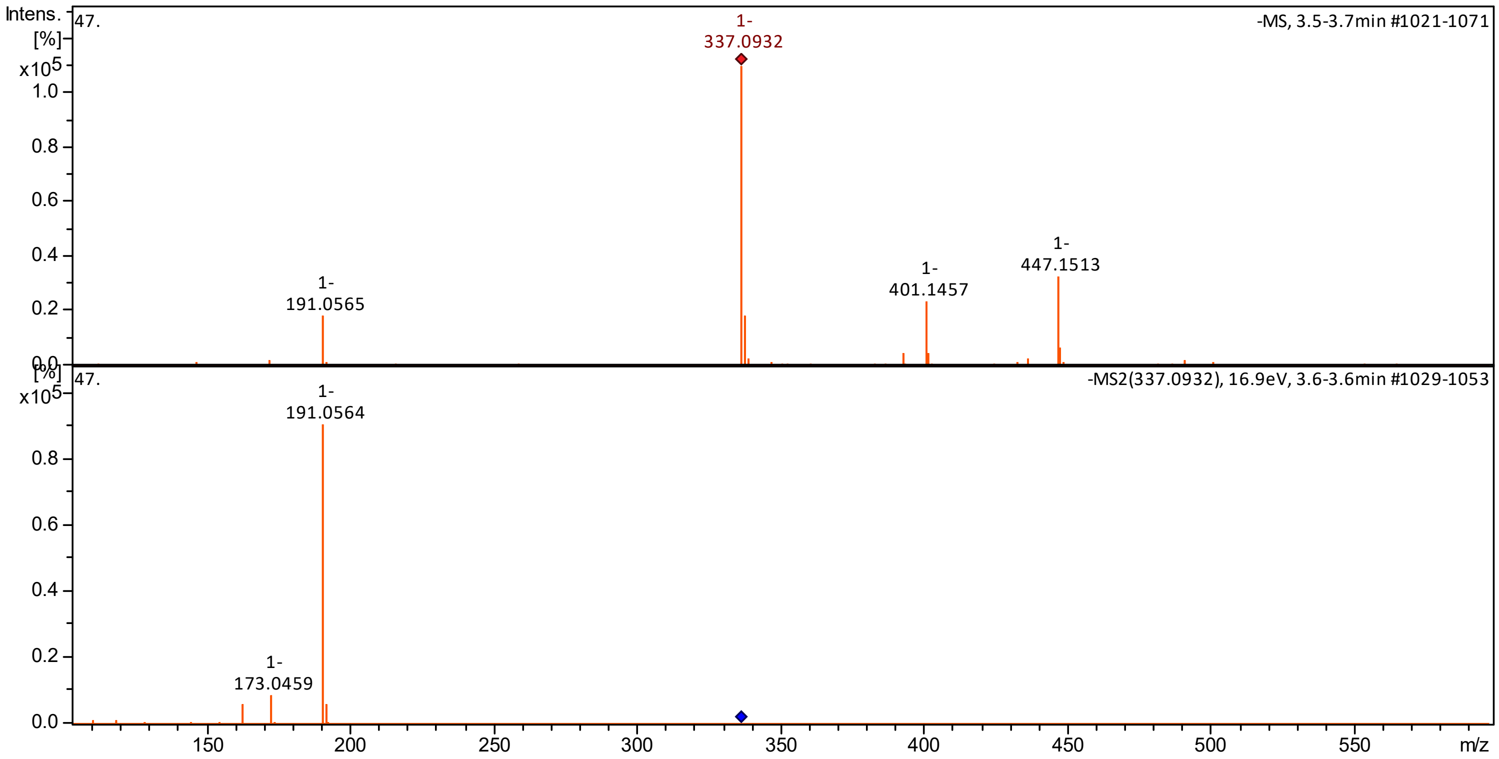

quinic acid derivative RT=5.1

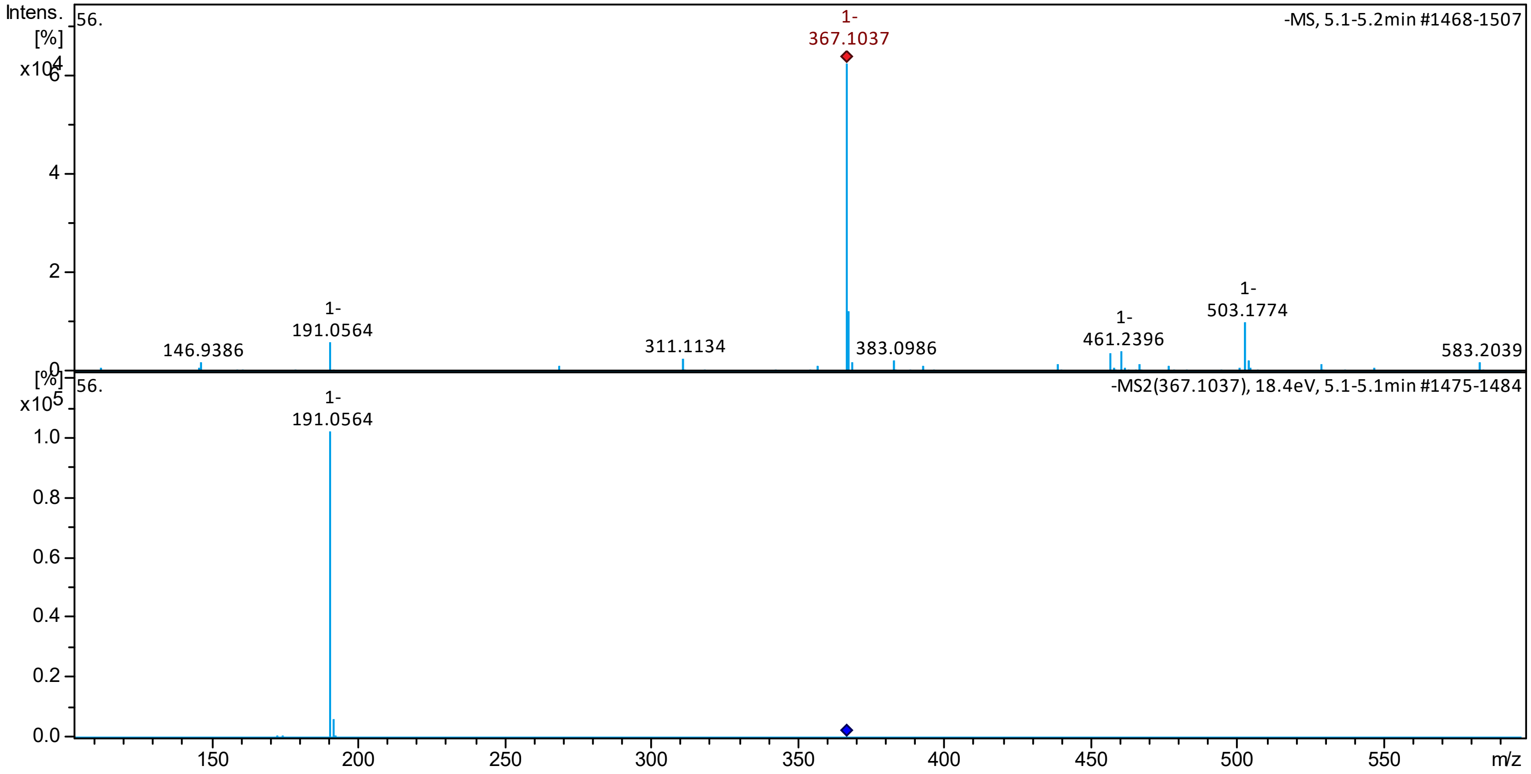

quercetin 3-O-rhamnoglucoside (rutin) RT=5.8

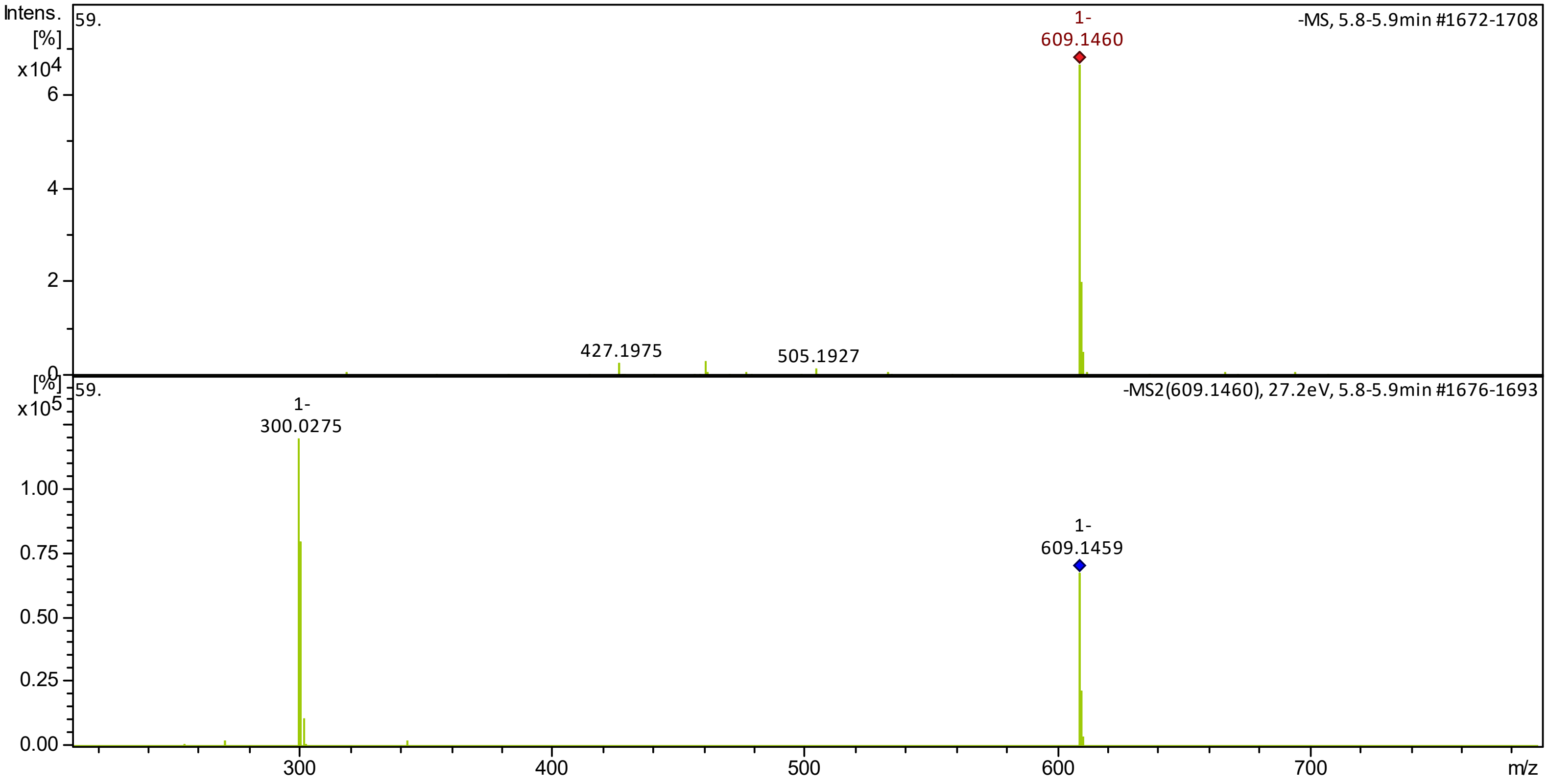

caffeic acid derivative RT=6.3

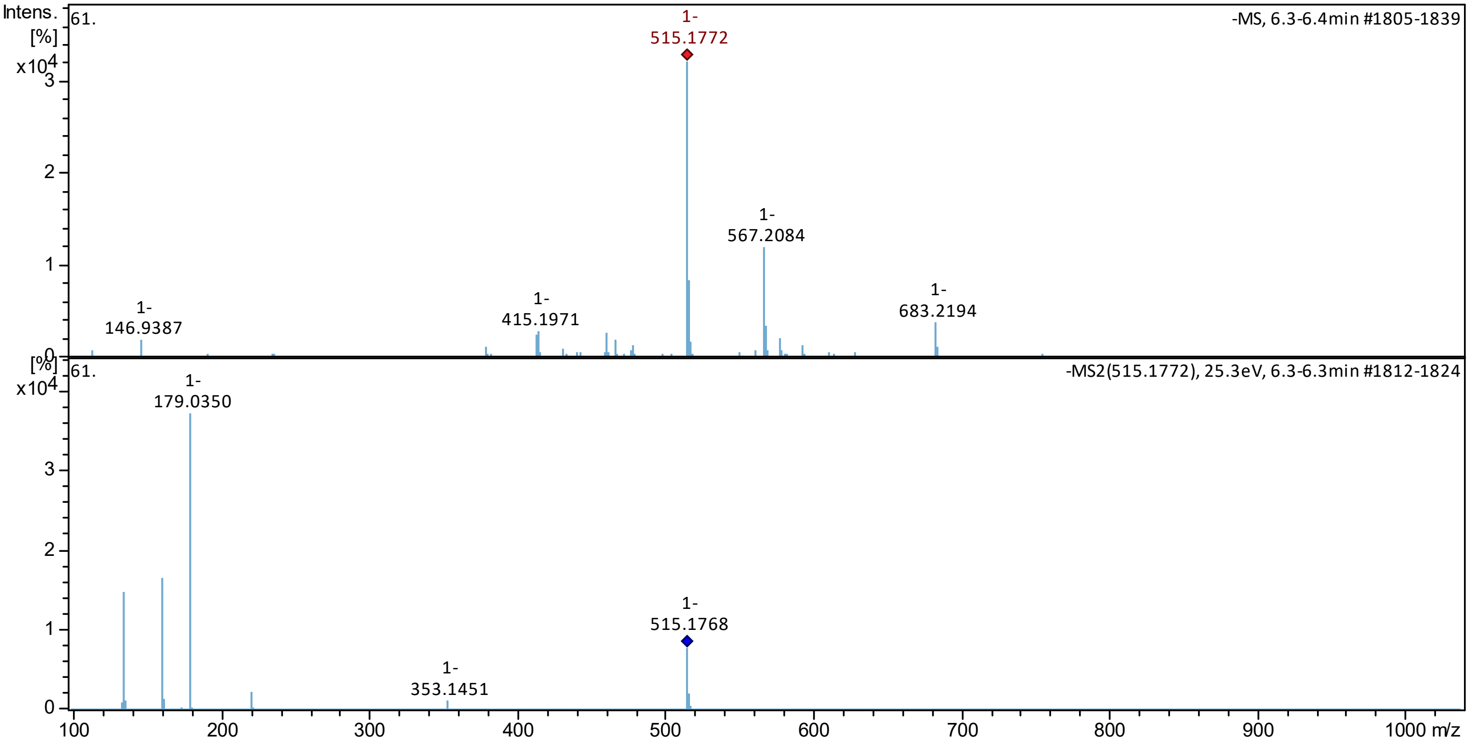

3,5-O- dicaffeoylguinic acid RT=6.8

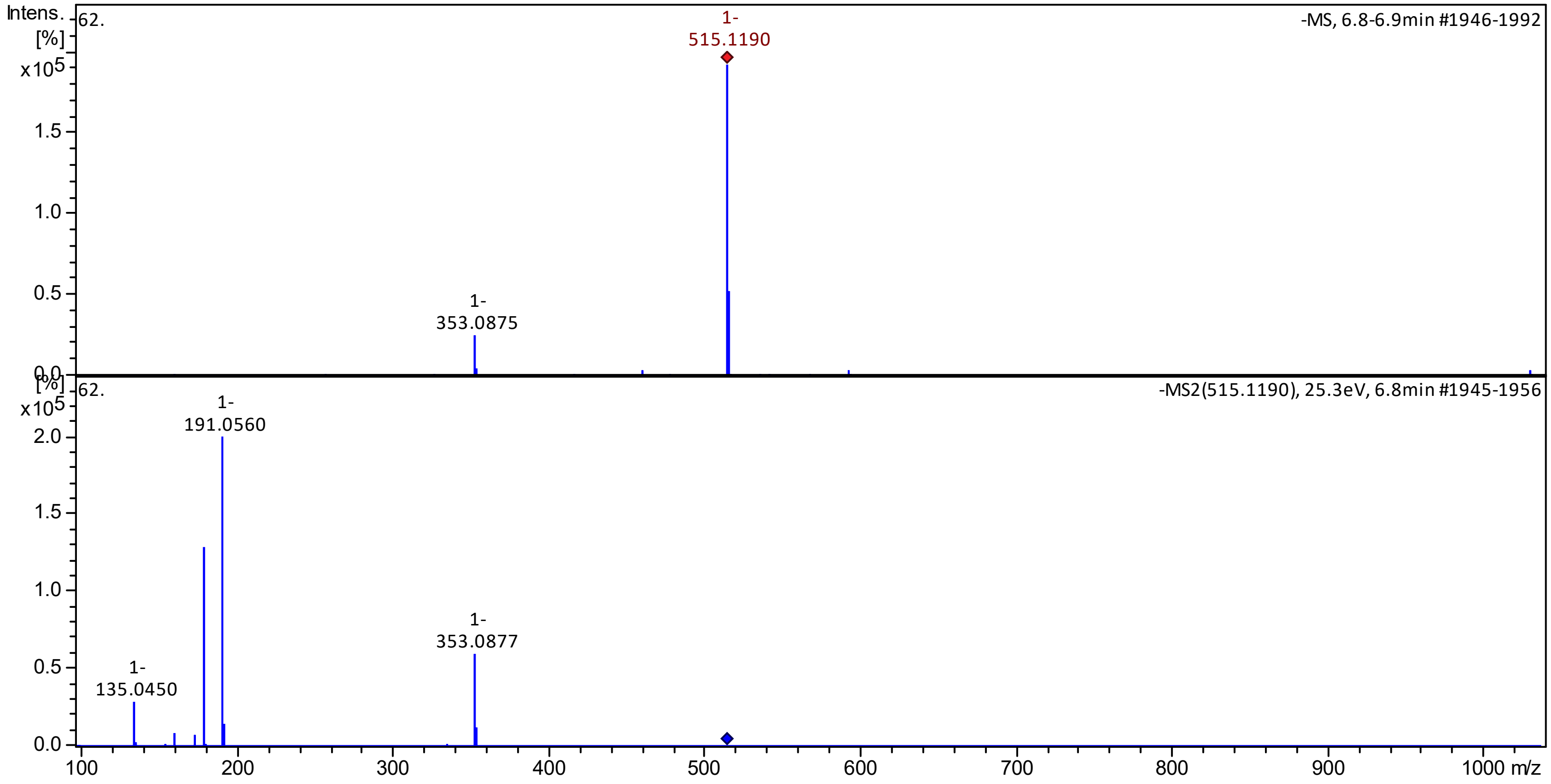

feruloylquinic acid derivative RT=7.3

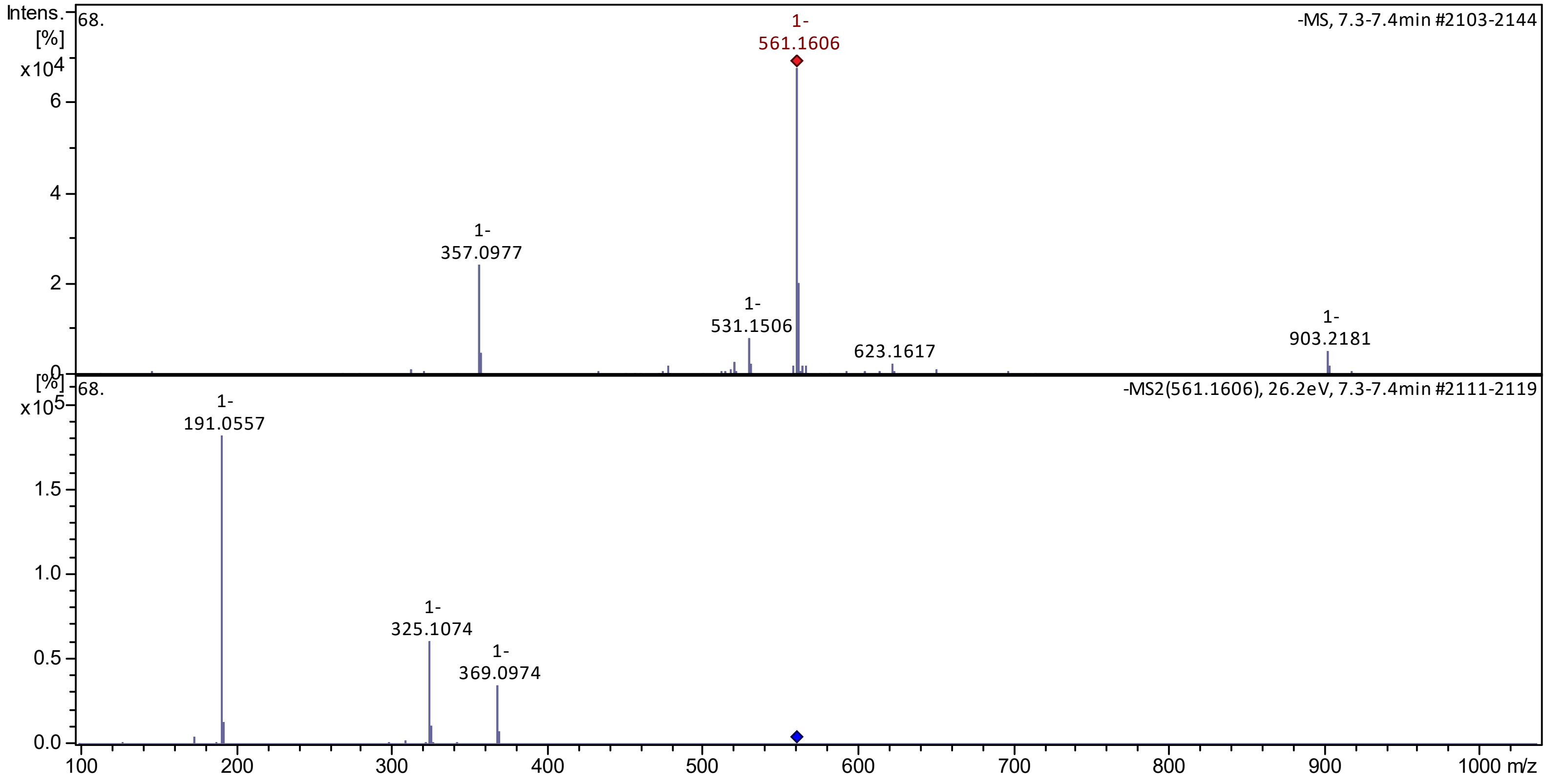

catechin derivative RT=11.7

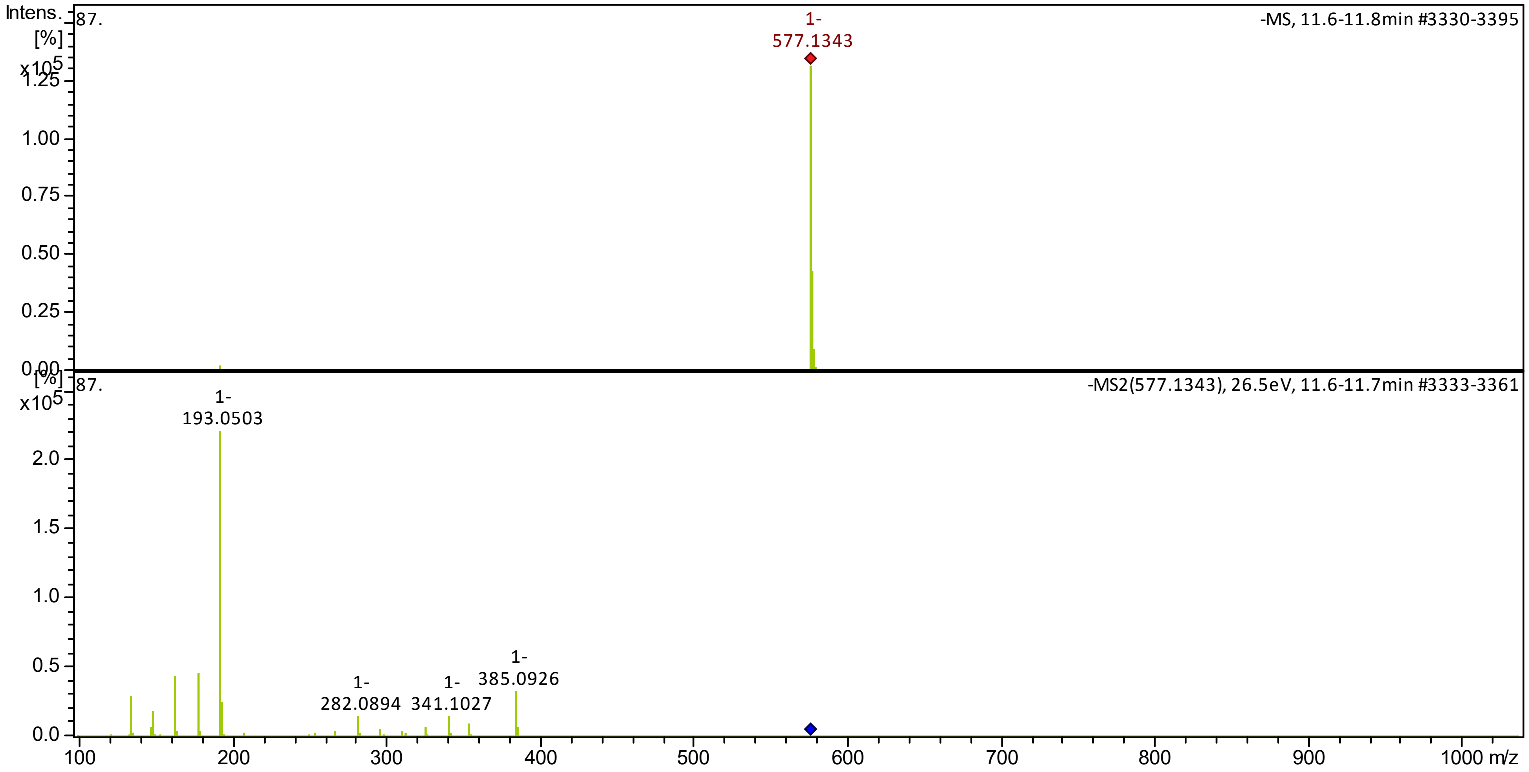

(+) - procyanidin B2 RT=13.2

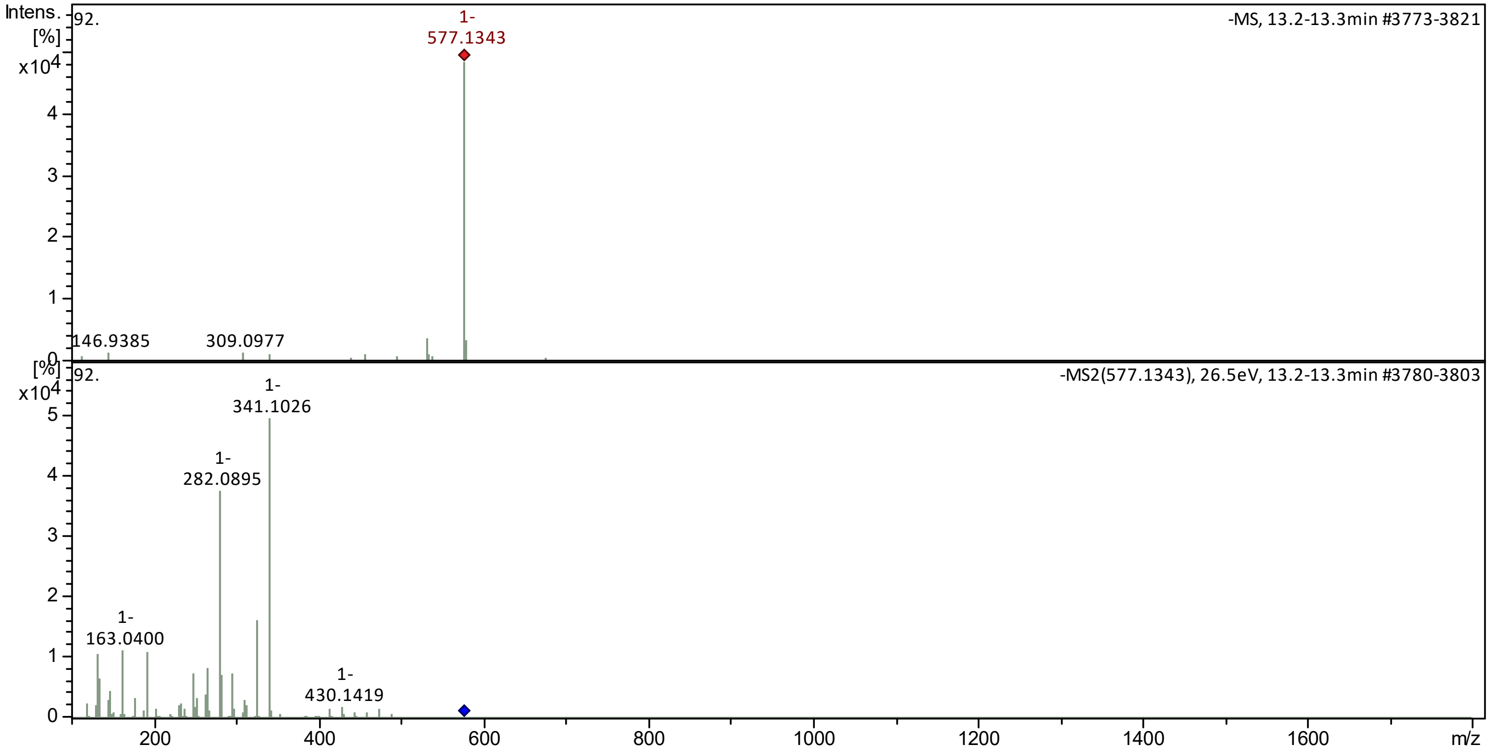

dammarenolic acid-type triterpenoid RT=13.9

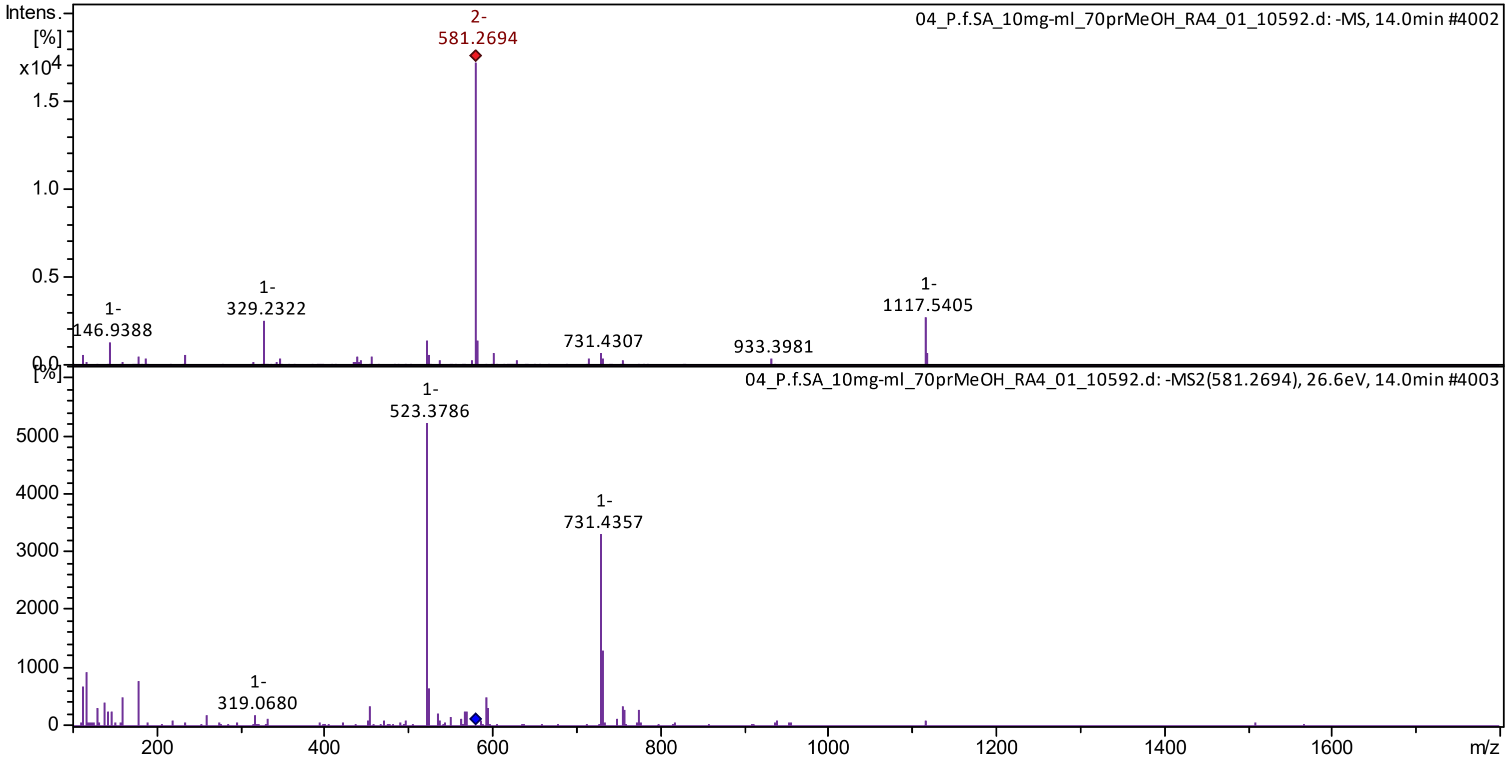

medicagenic acid- type triterpenoid RT=15.3

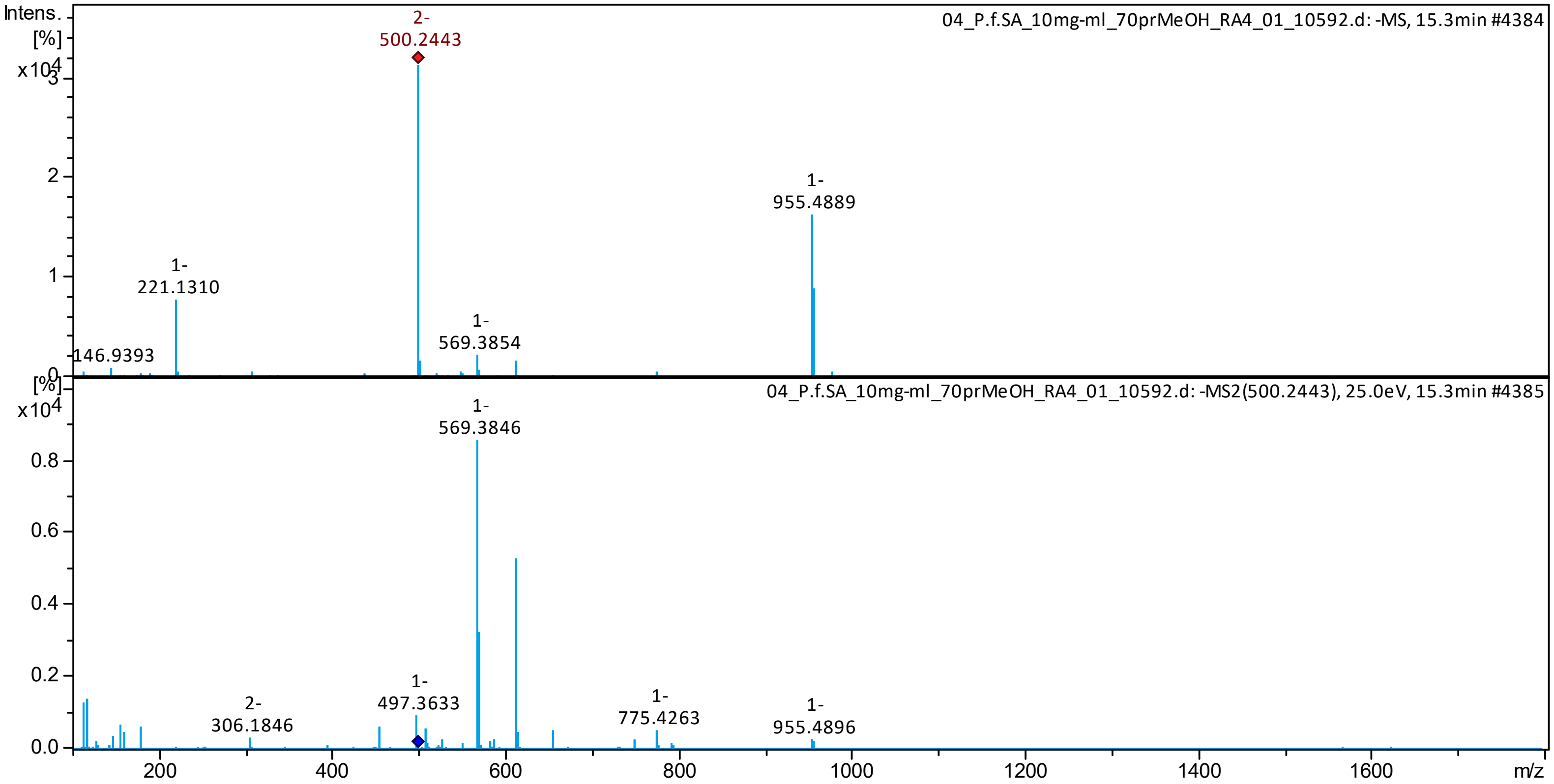

spinasaponin A/ zingibroside R1 (oleanane-type triterpenoid) RT=19.8

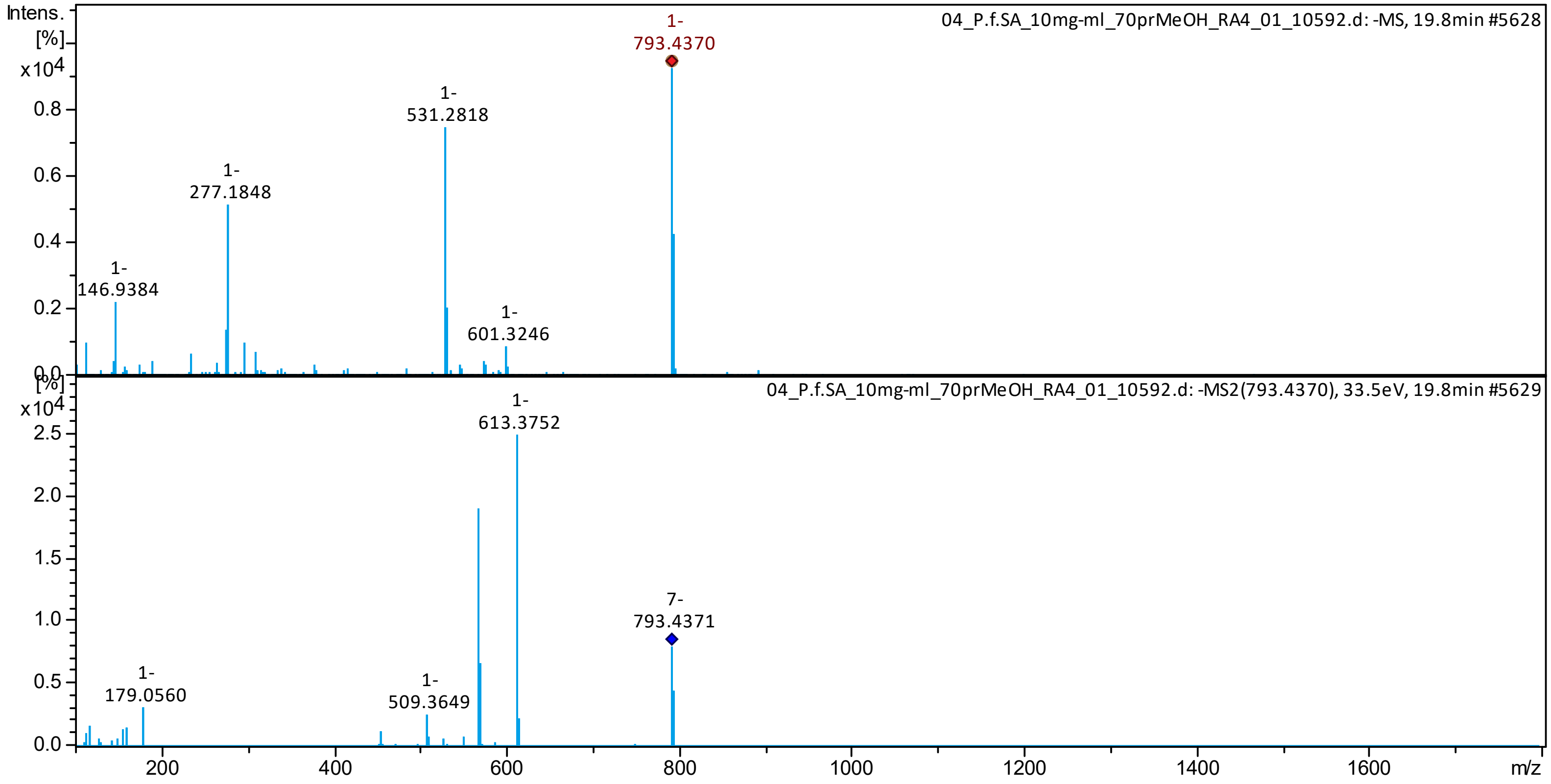

palmitate acid derivative RT=26.7

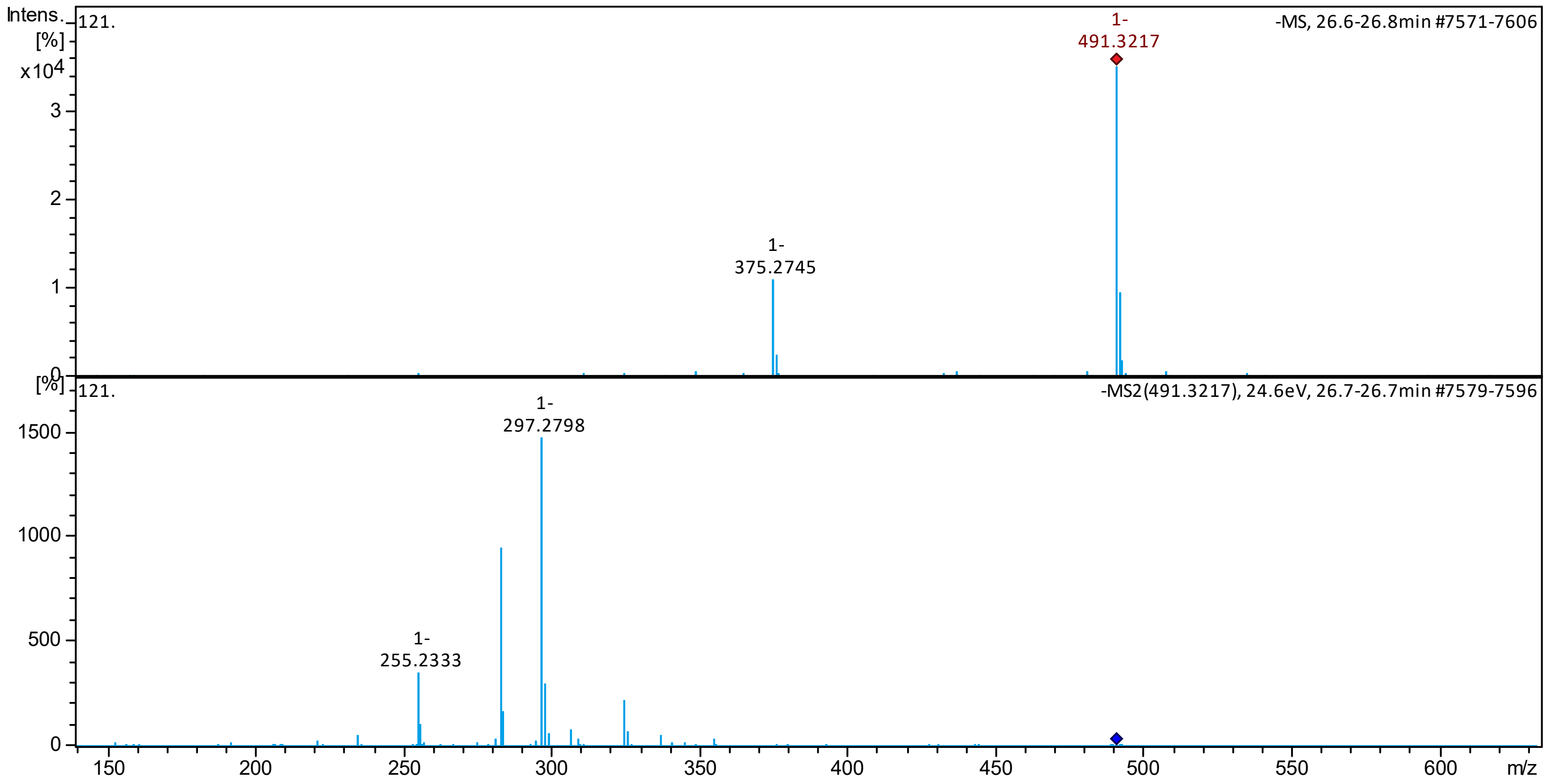

linoleic acid RT=26.9

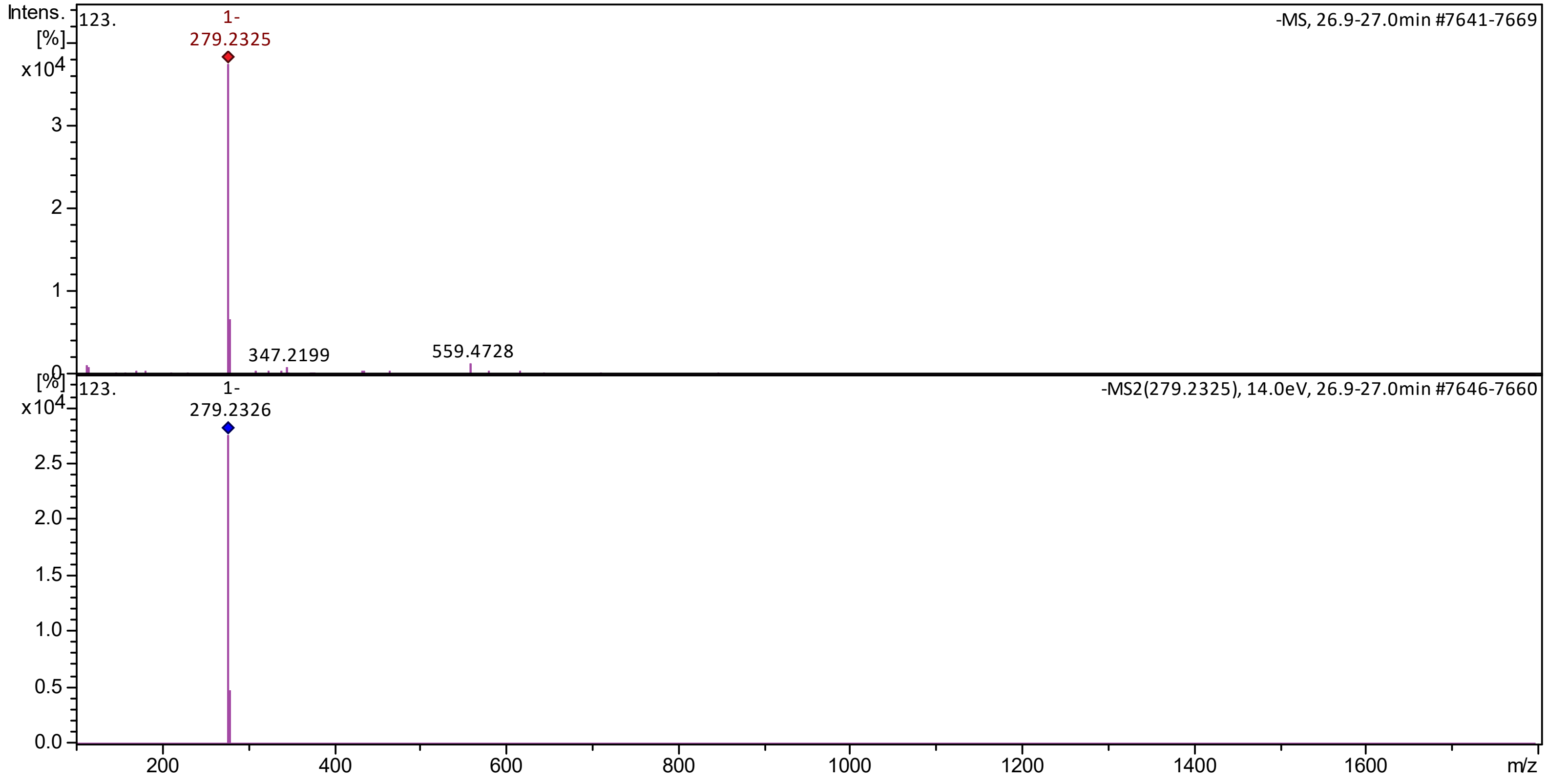

c)

L-malic acid RT=0.7

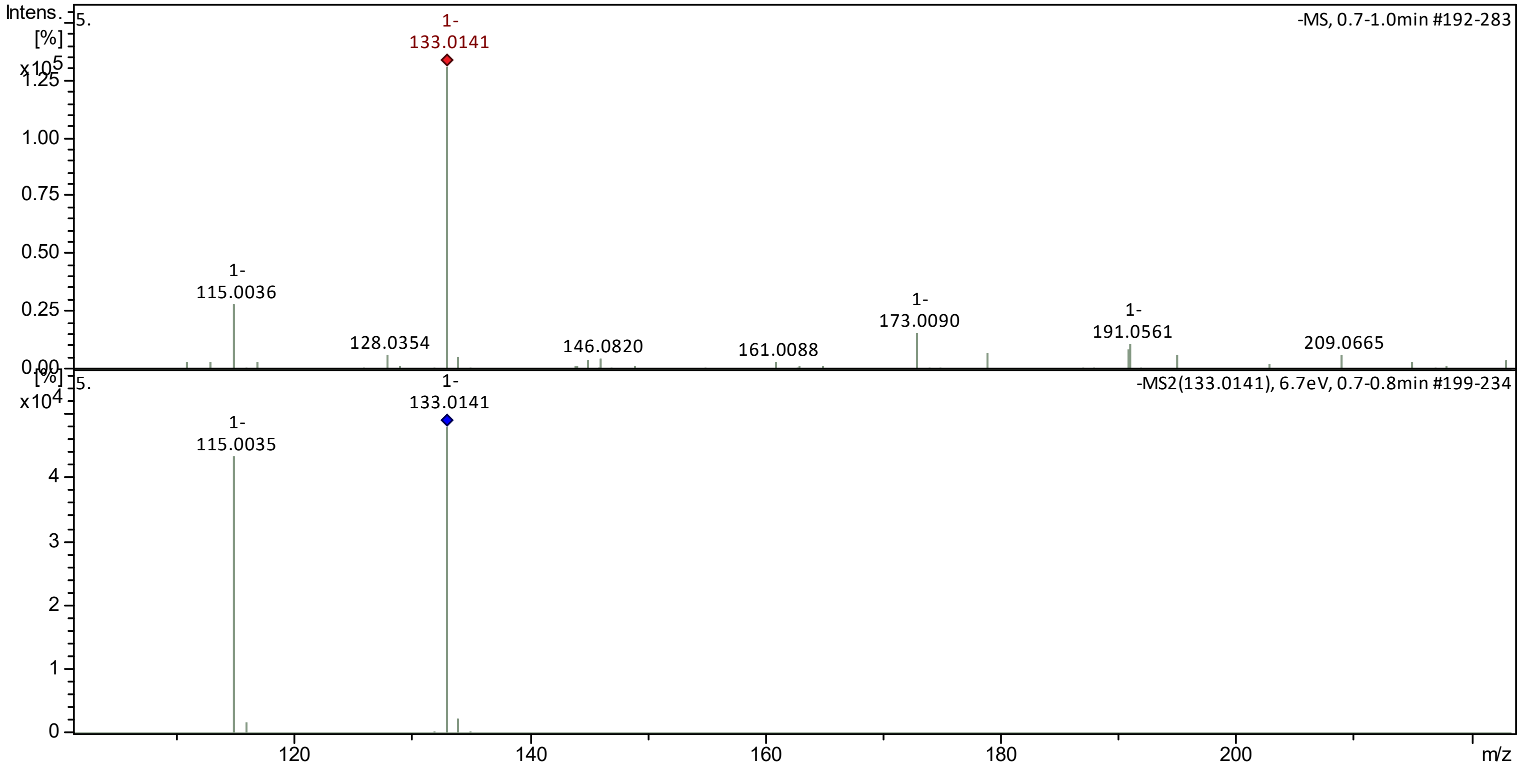

4-oxoproline RT=0.9

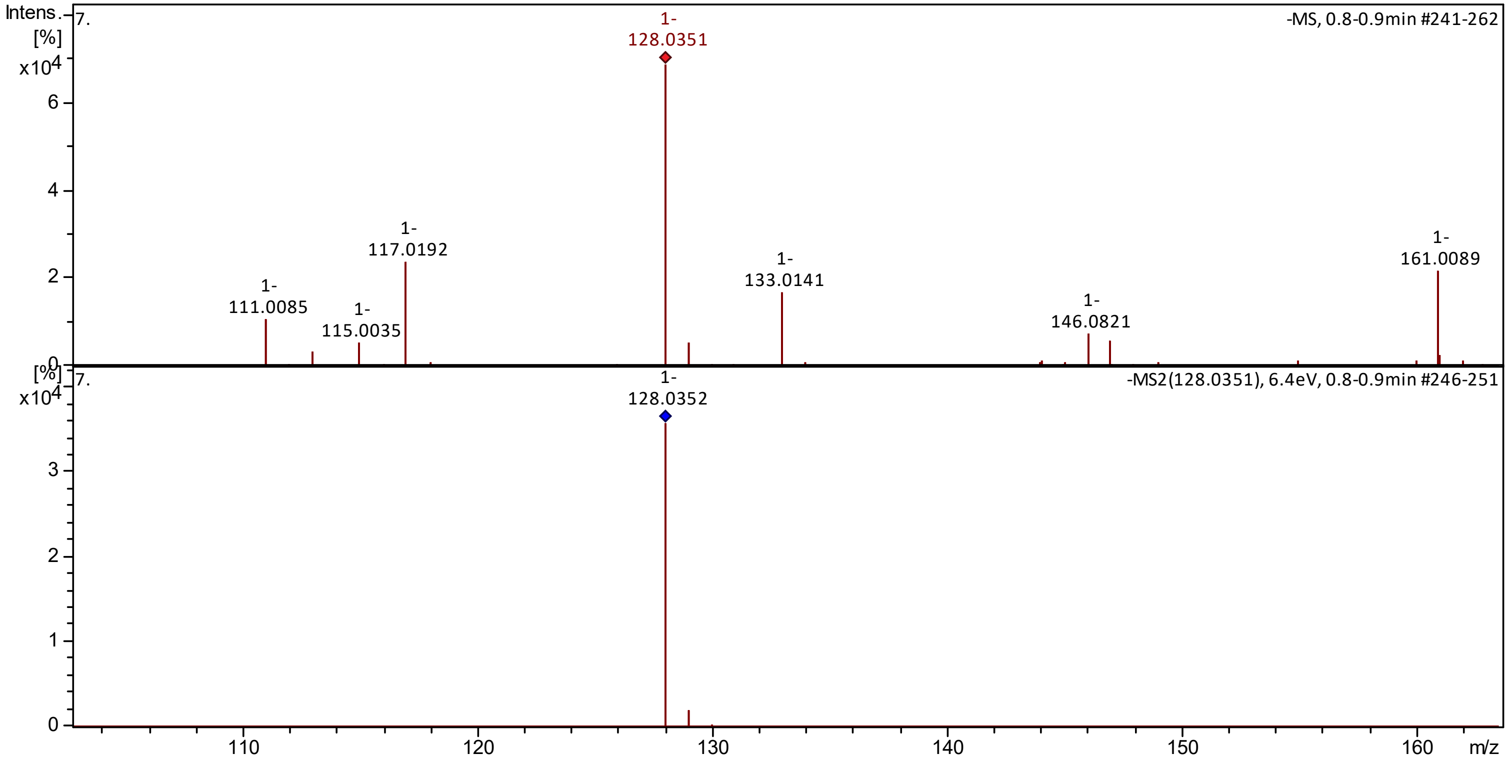

L-tryptophan RT=1.9

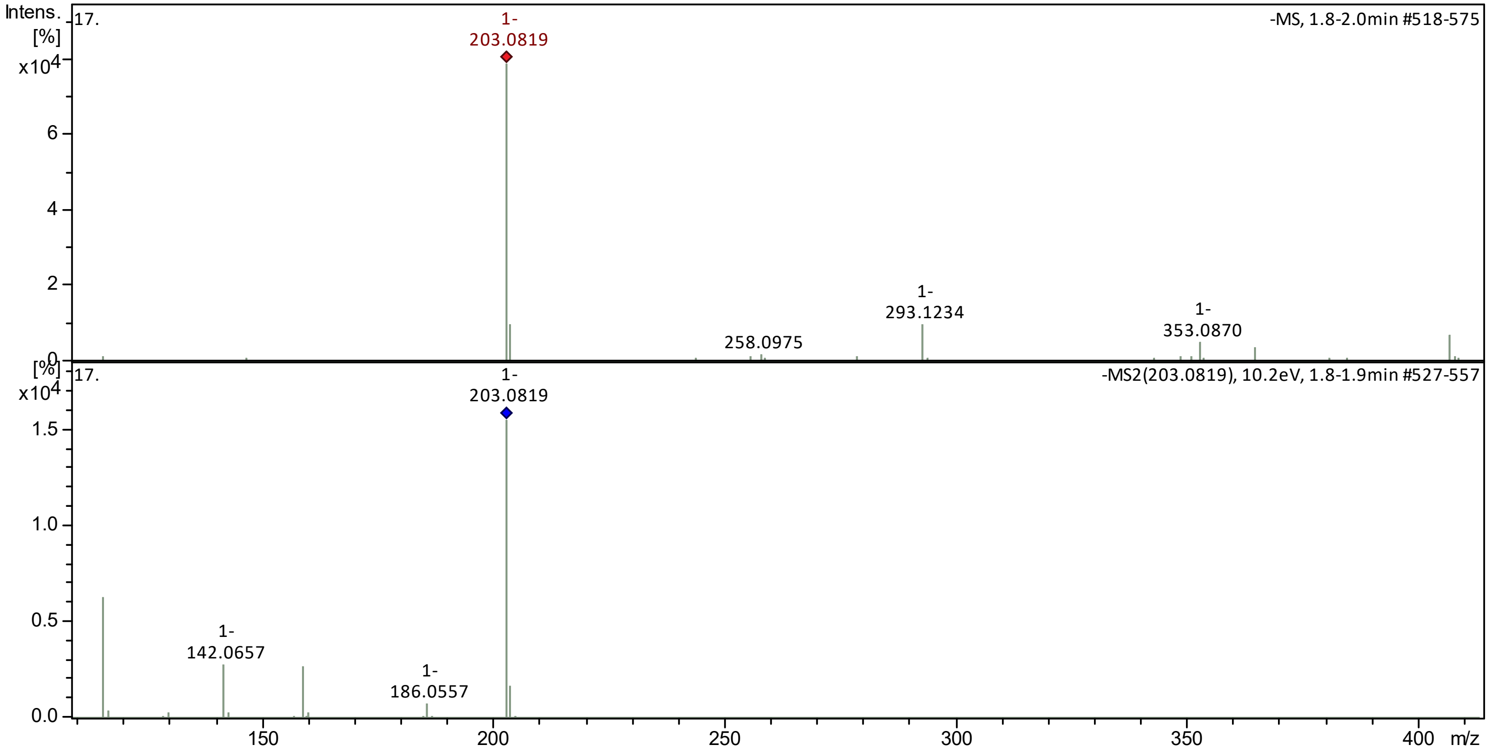

1/3/5-O-caffeoylguinic acid RT=2.7

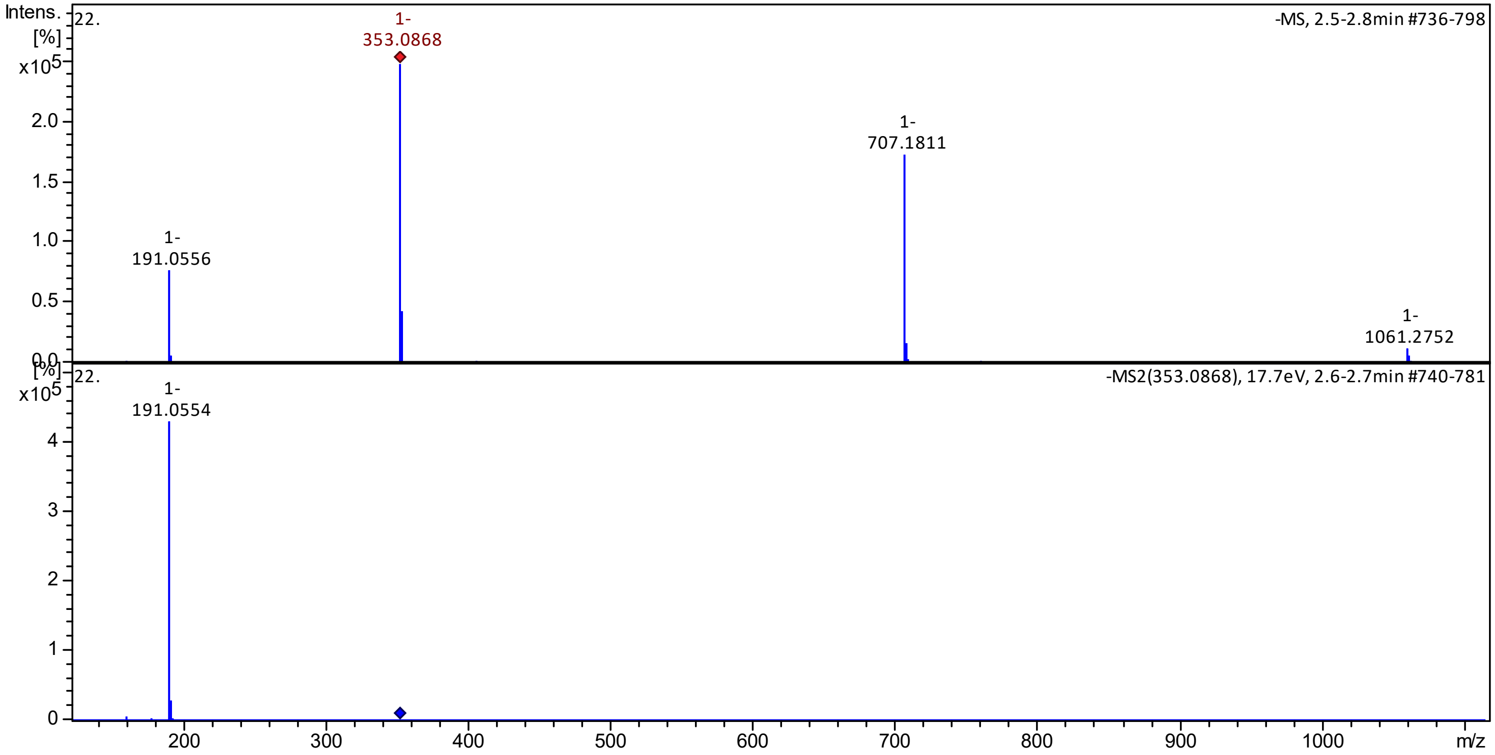

quinic acid derivative RT=5.1

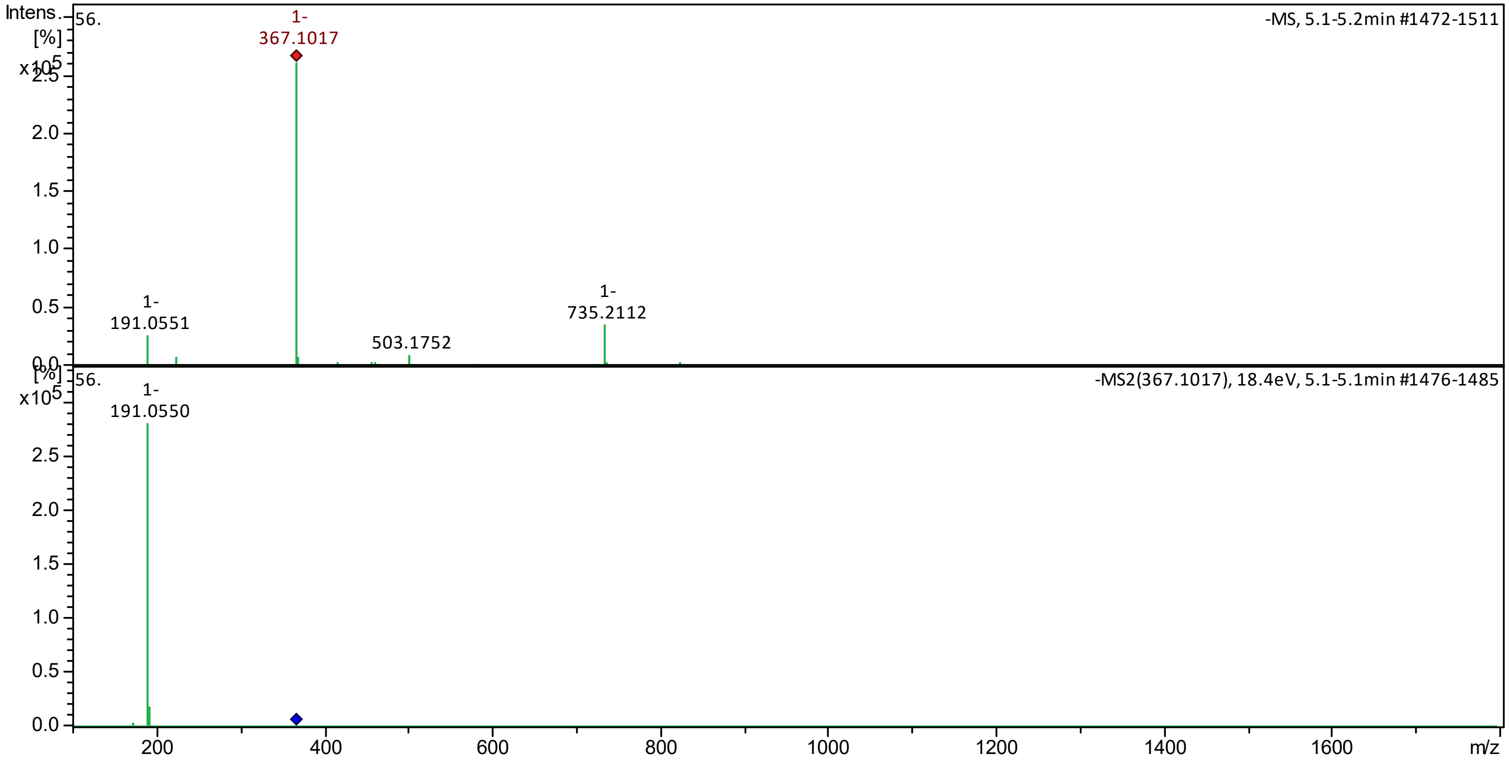

quercetin 3-O-rhamnoglucoside (rutin)   RT=5.8

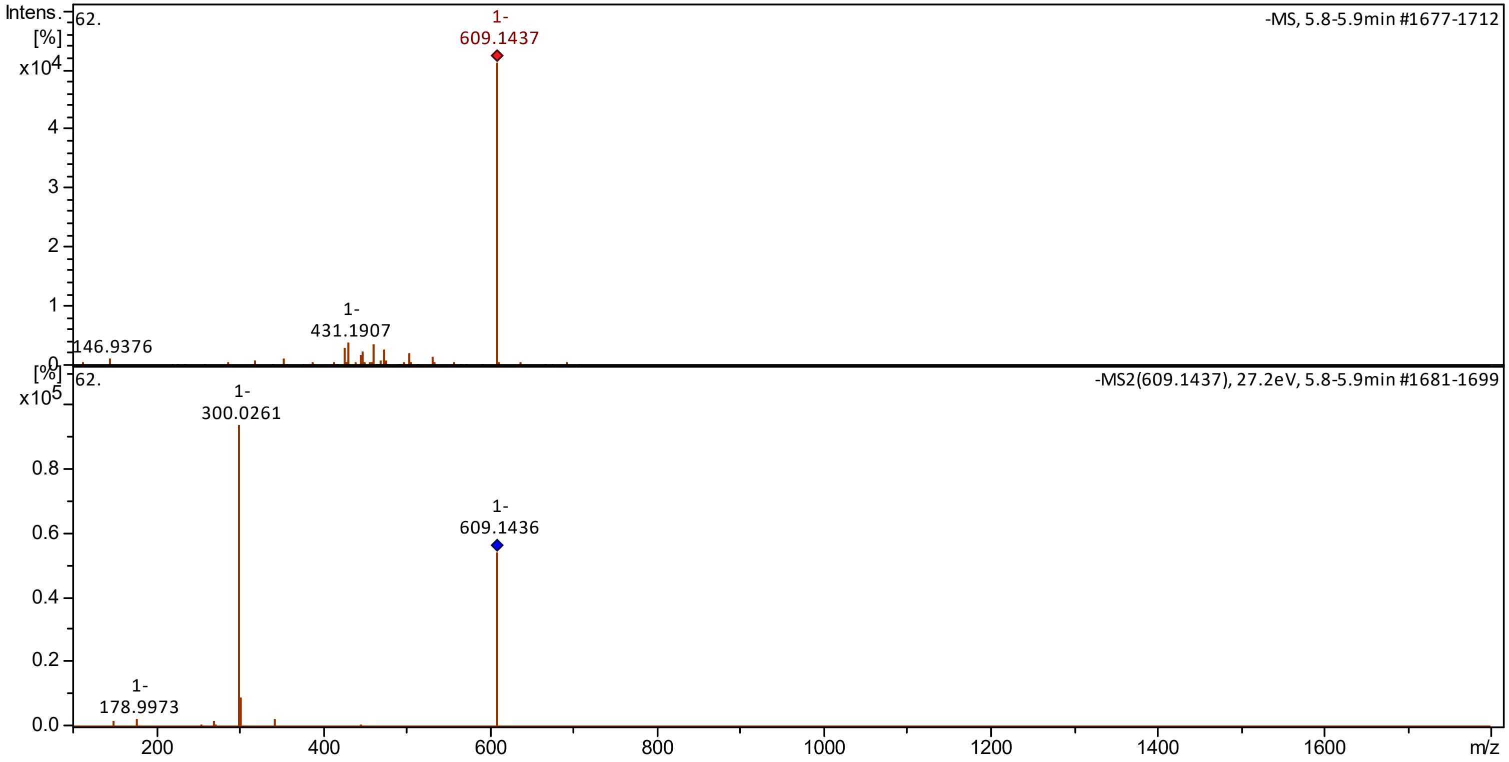

caffeic acid derivative RT=6.3

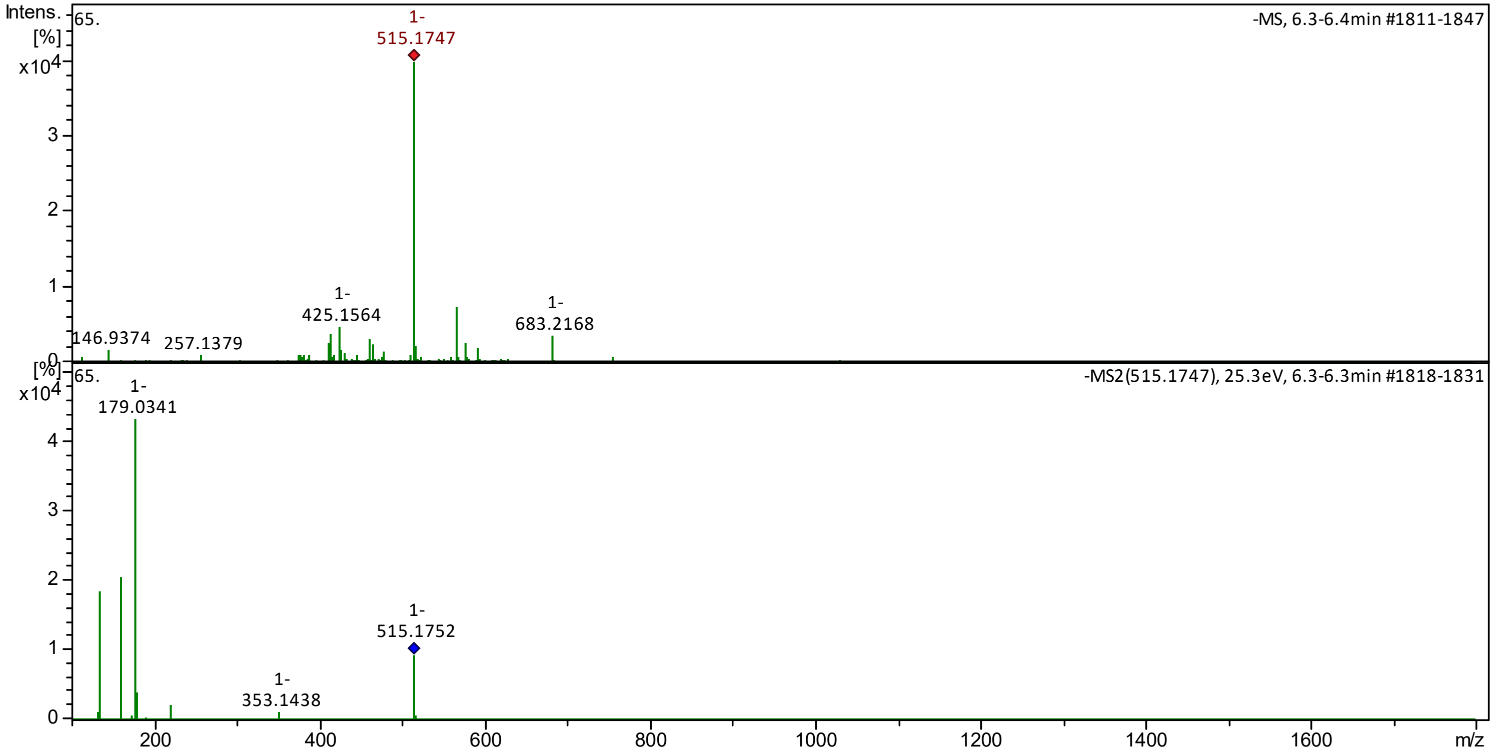

feruloylquinic acid derivative RT=7.4

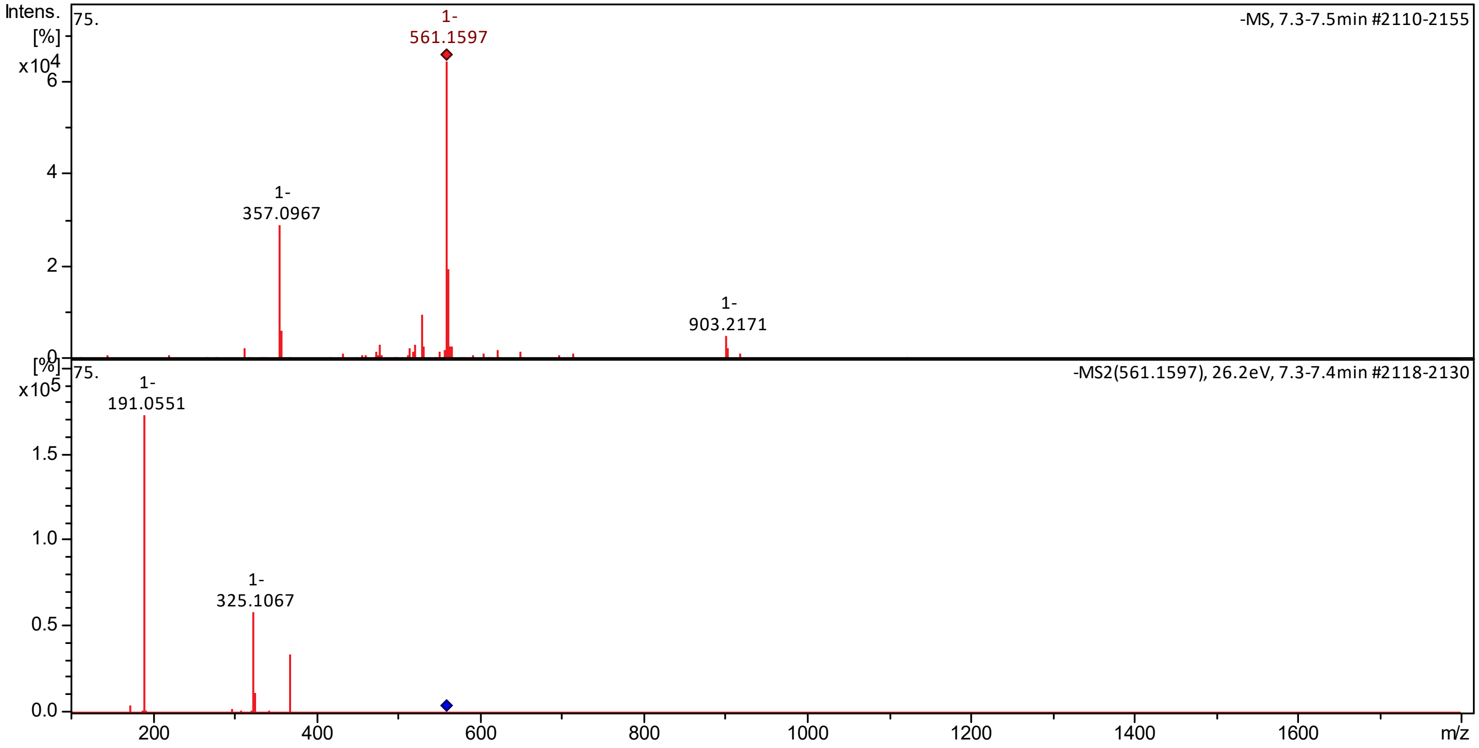

catechin derivative RT=11.7

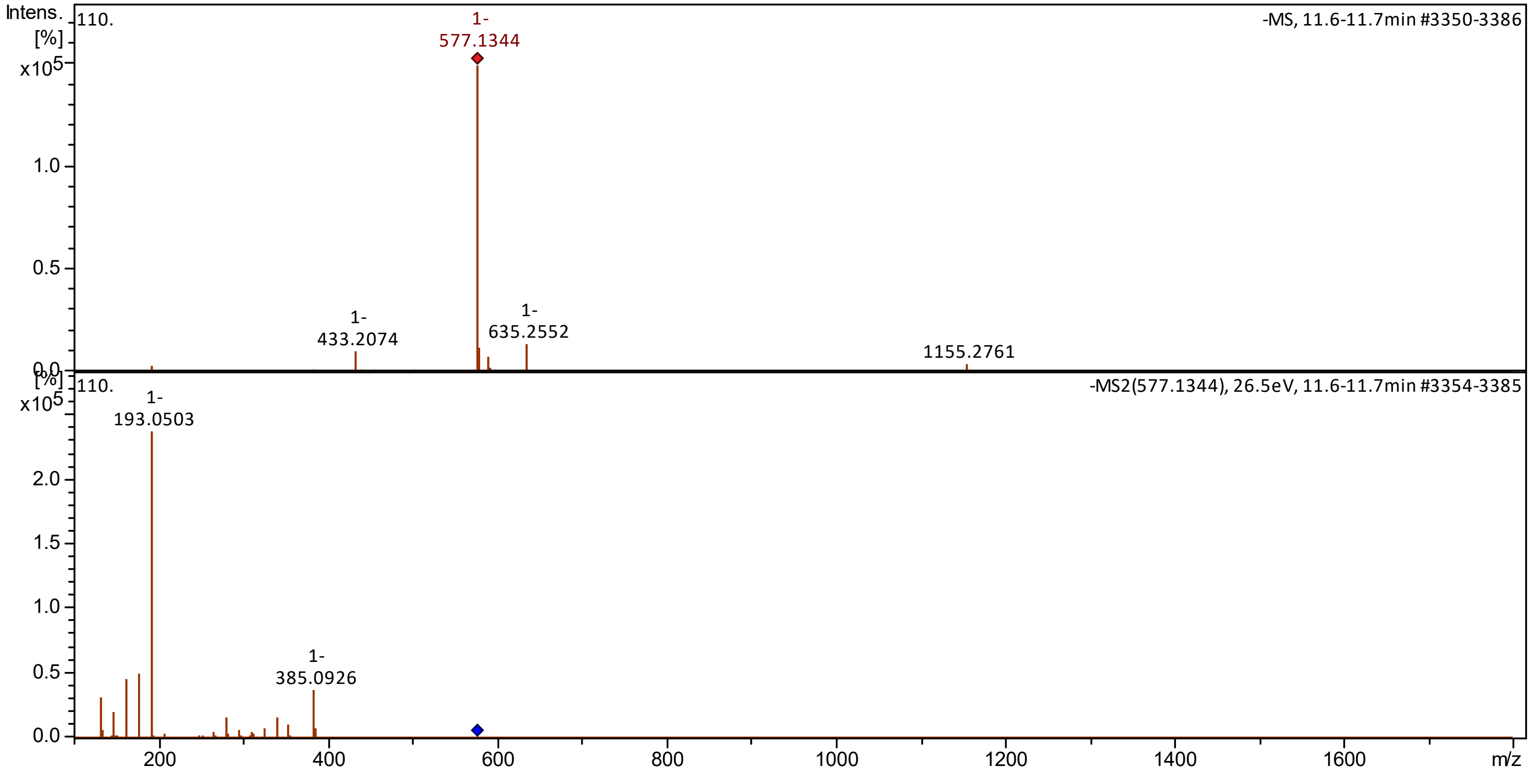

(+) - procyanidin B2 RT=13.0

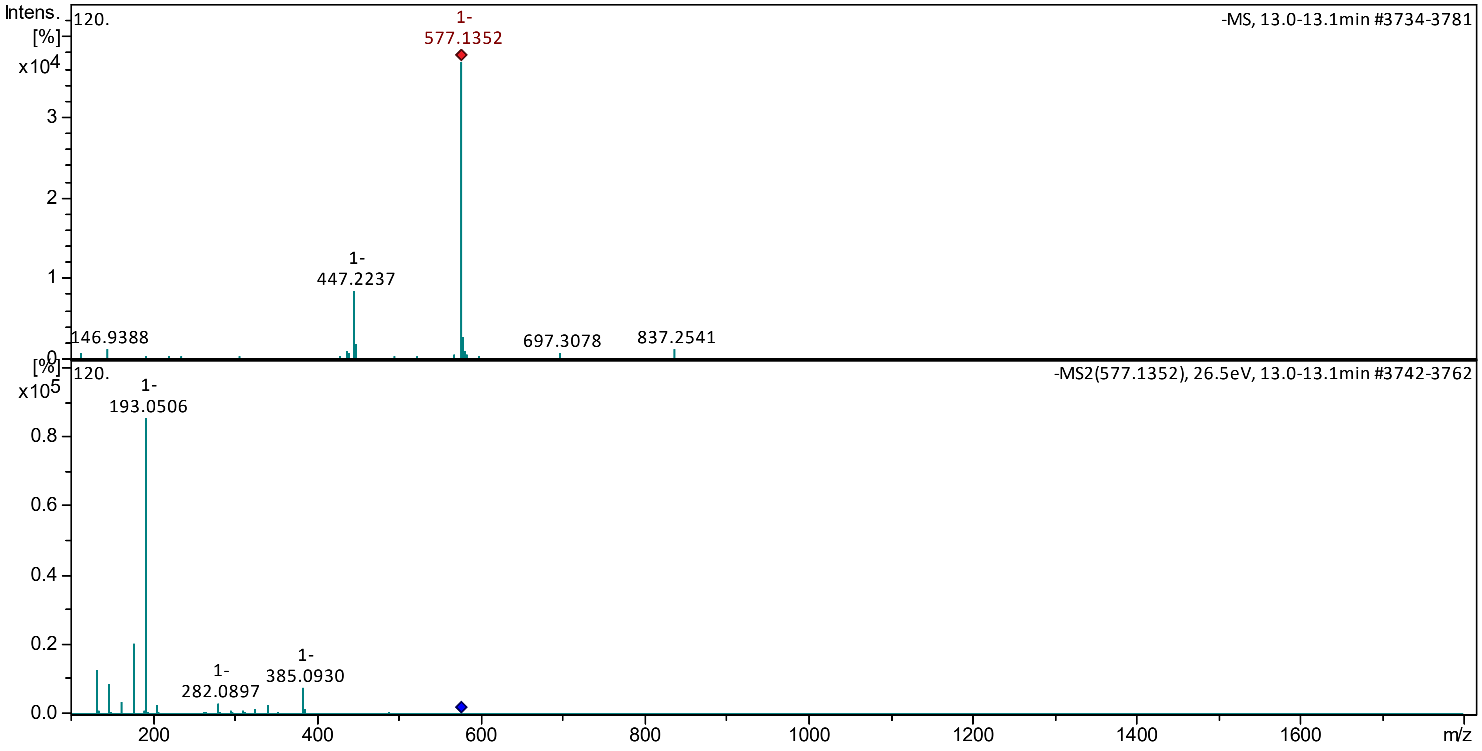

oleanane-type triterpenoid RT=14.0

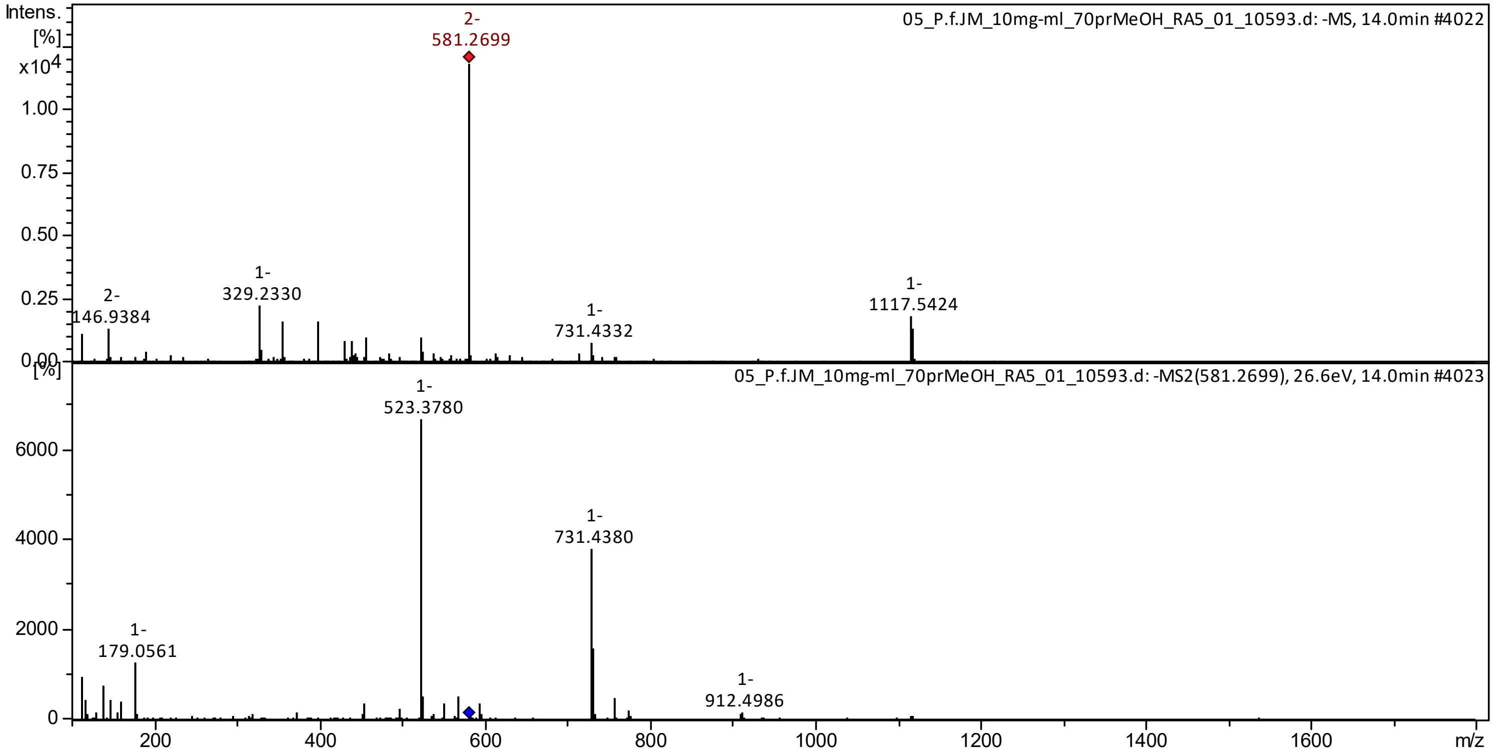

medicagenic acid- type triterpenoid RT=15.3

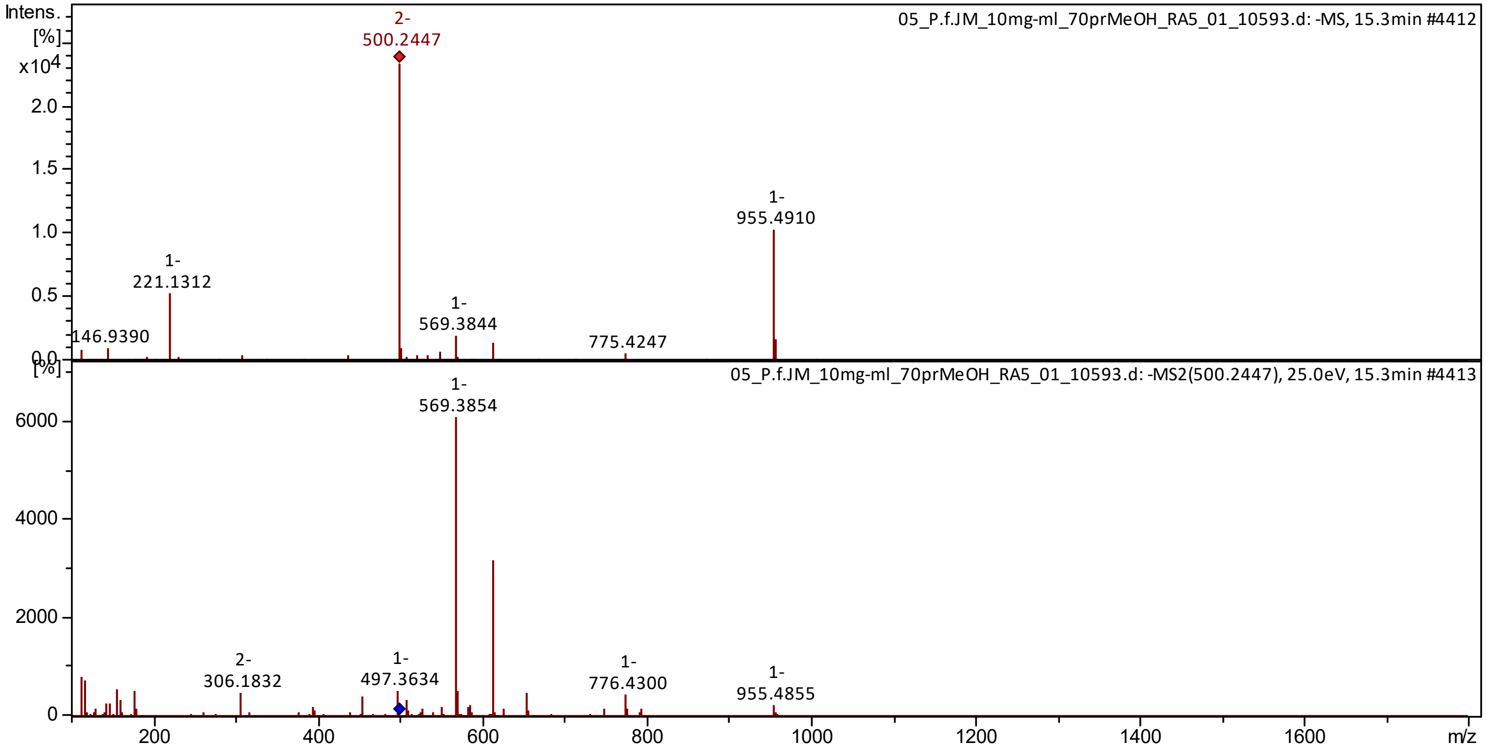

spinasaponin A/ zingibroside R1 (oleanane-type triterpenoid) RT=19.8

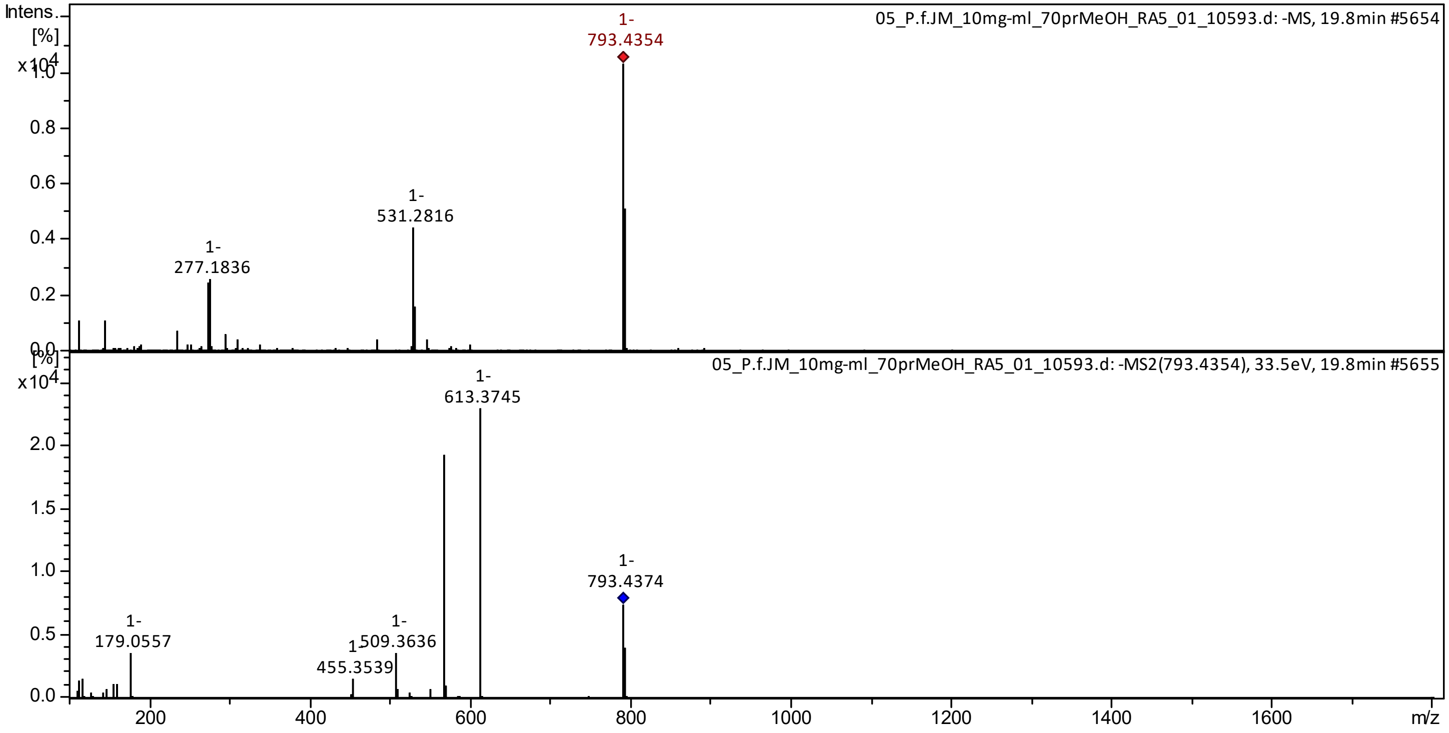

octadecanoic acid derivative RT=26.7

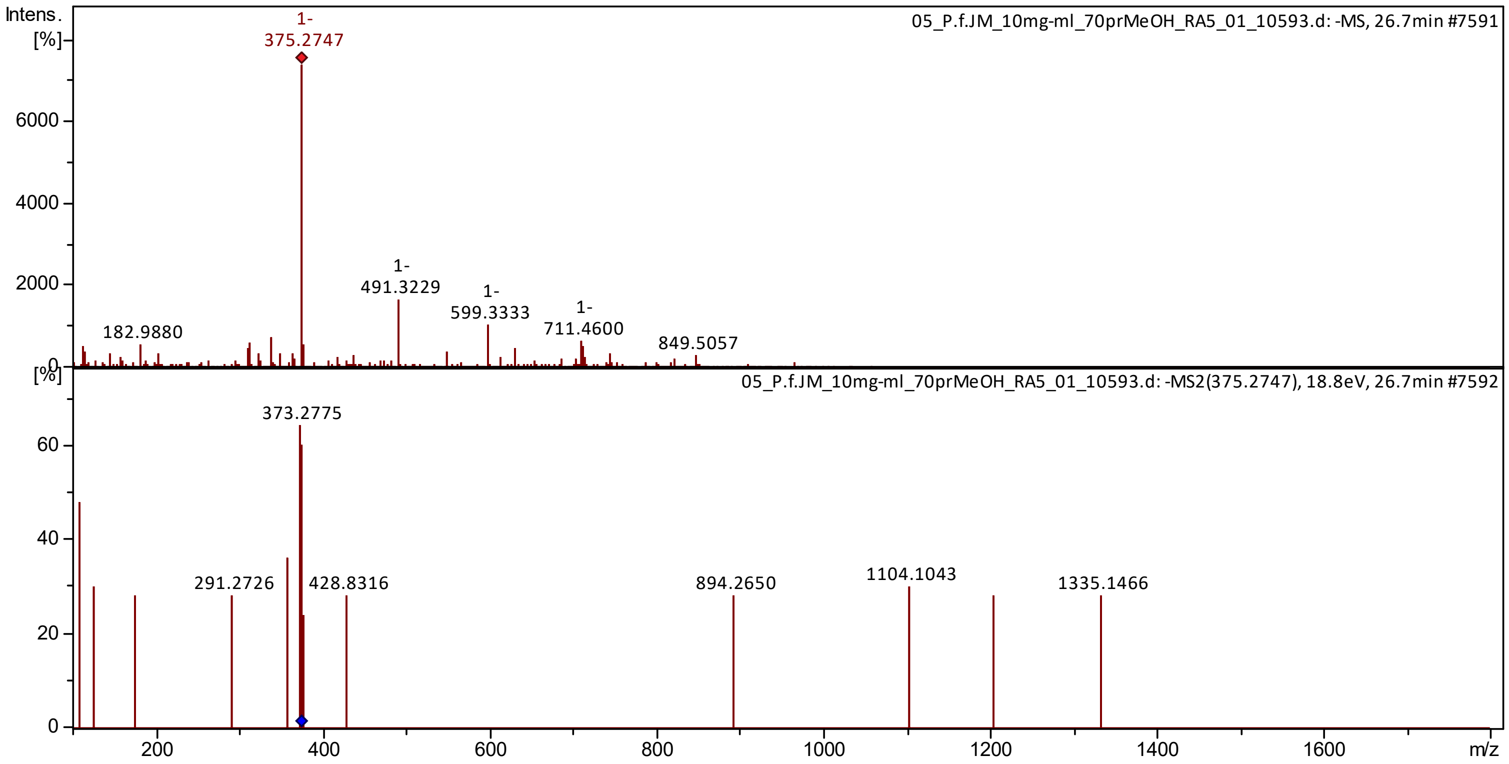

d)

L-malic acid RT=0.7

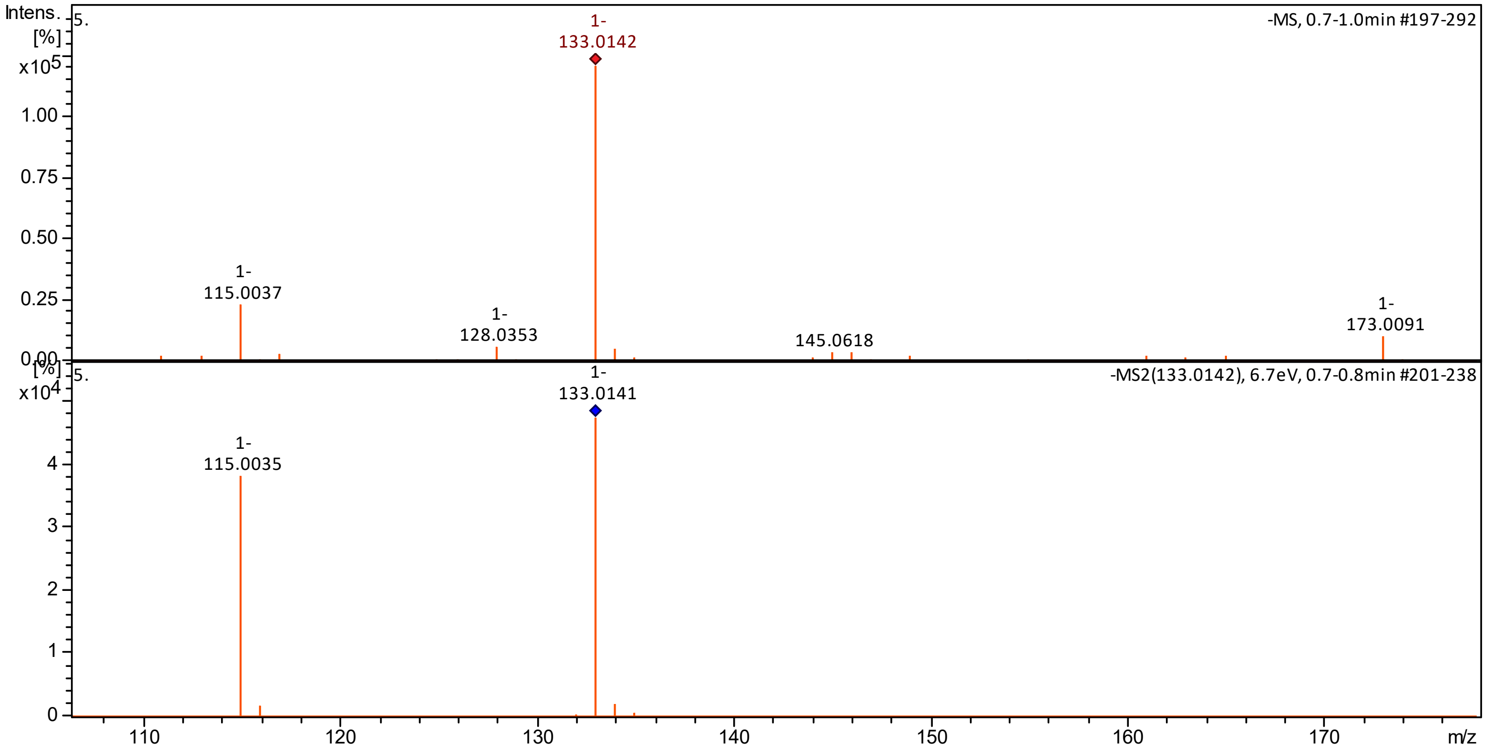

4-oxoproline RT=0.9

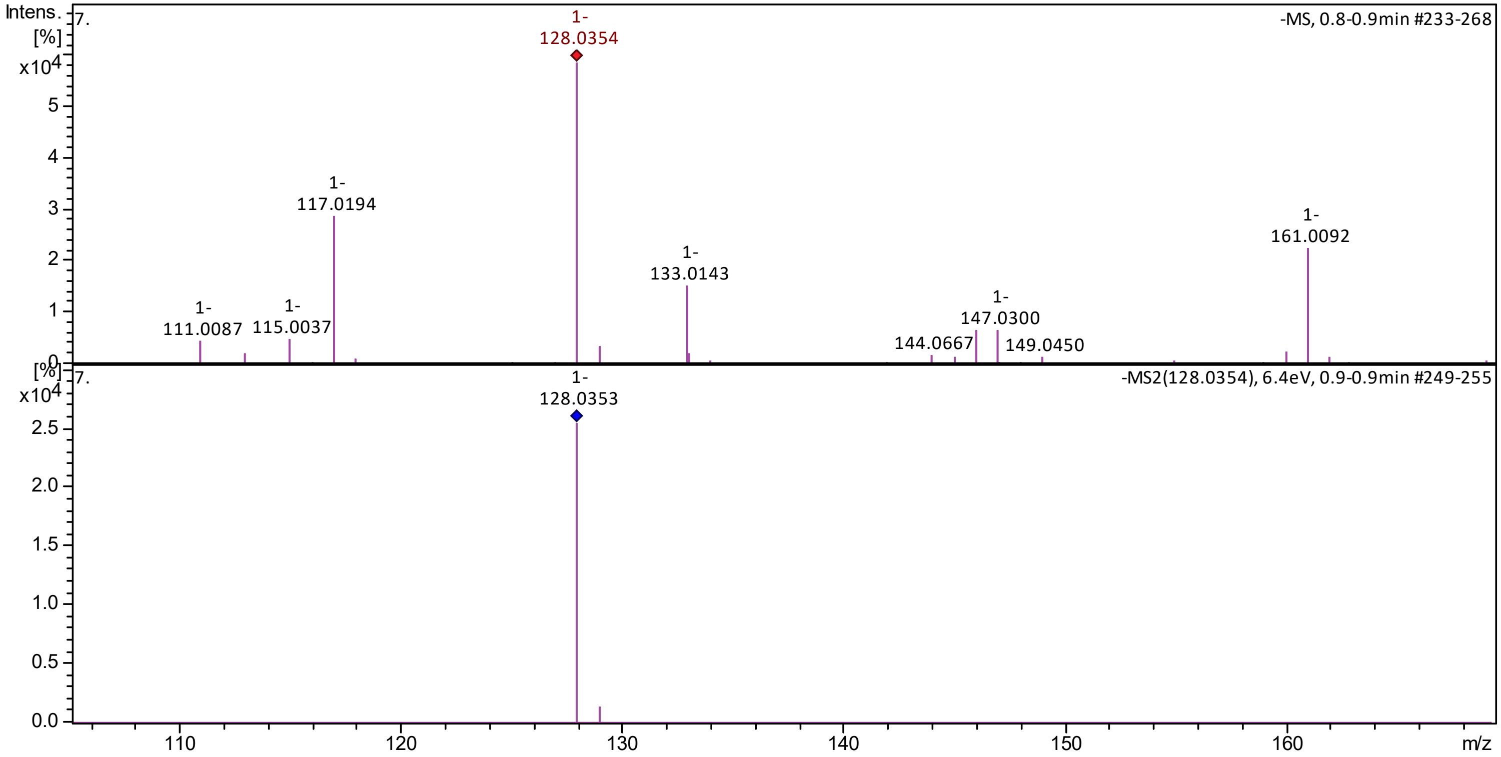

1/3/5-O-caffeoylguinic acid RT=2.7

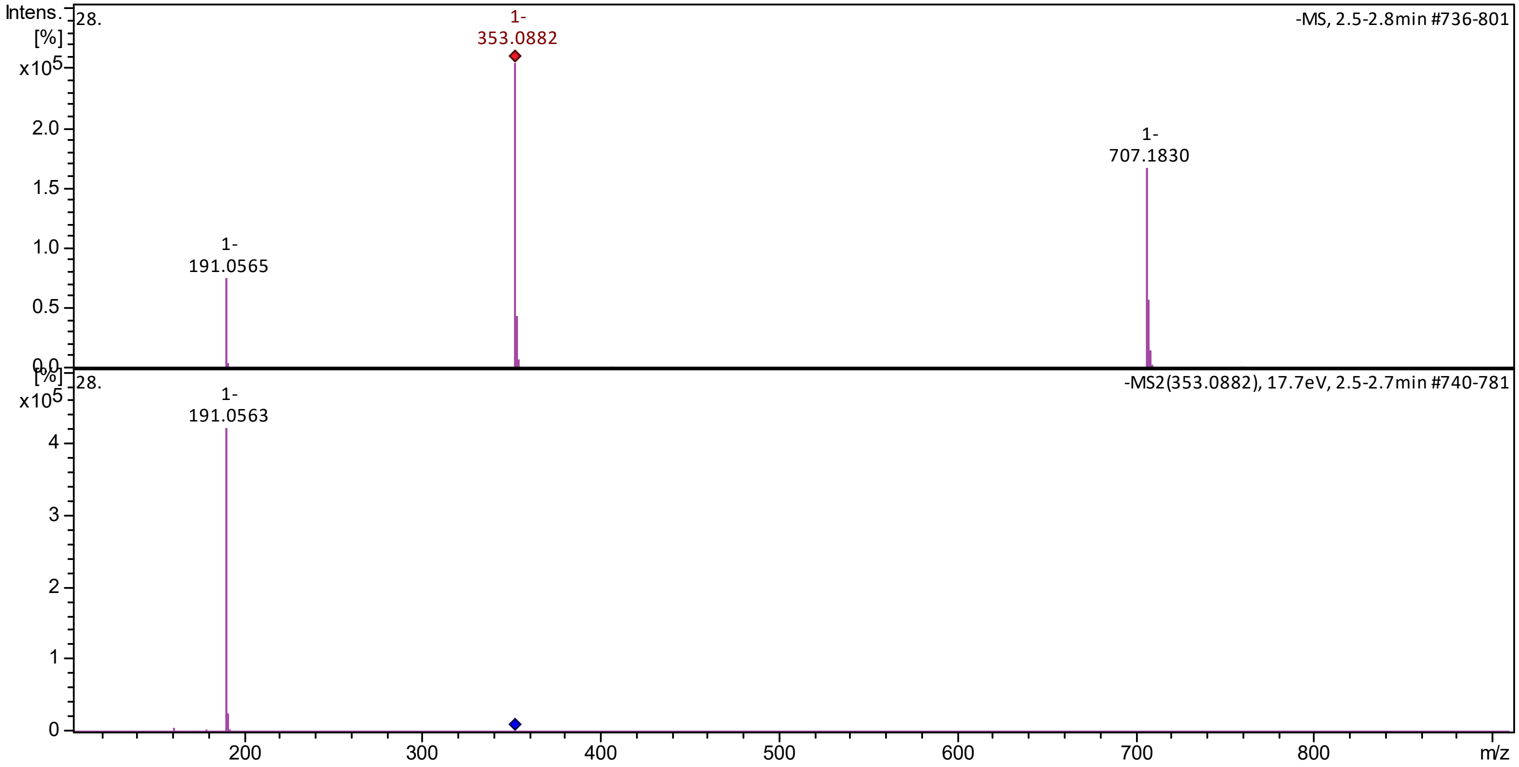

5-O-feruloylquinic acid RT=4.3

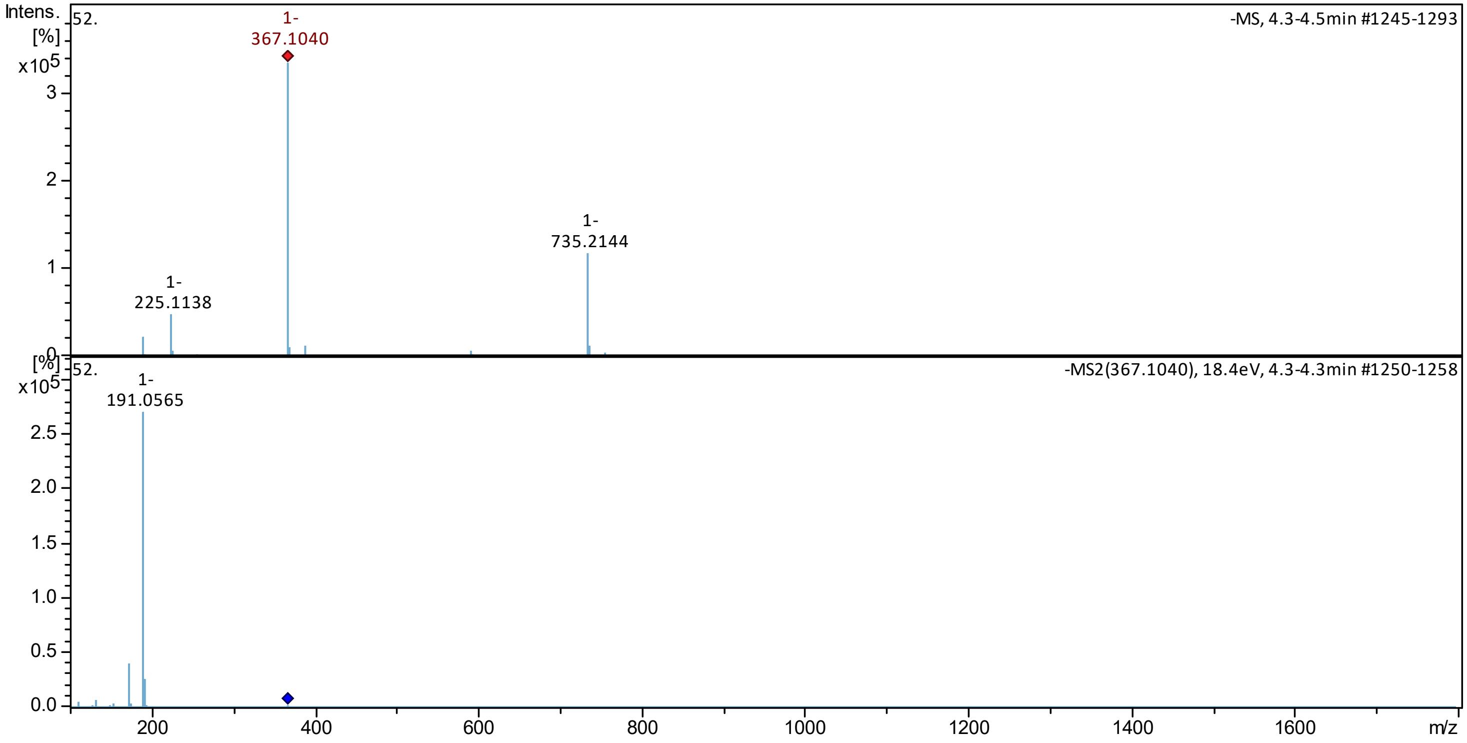

quercetin 3-O-rhamnoglucoside (rutin) RT=5.8

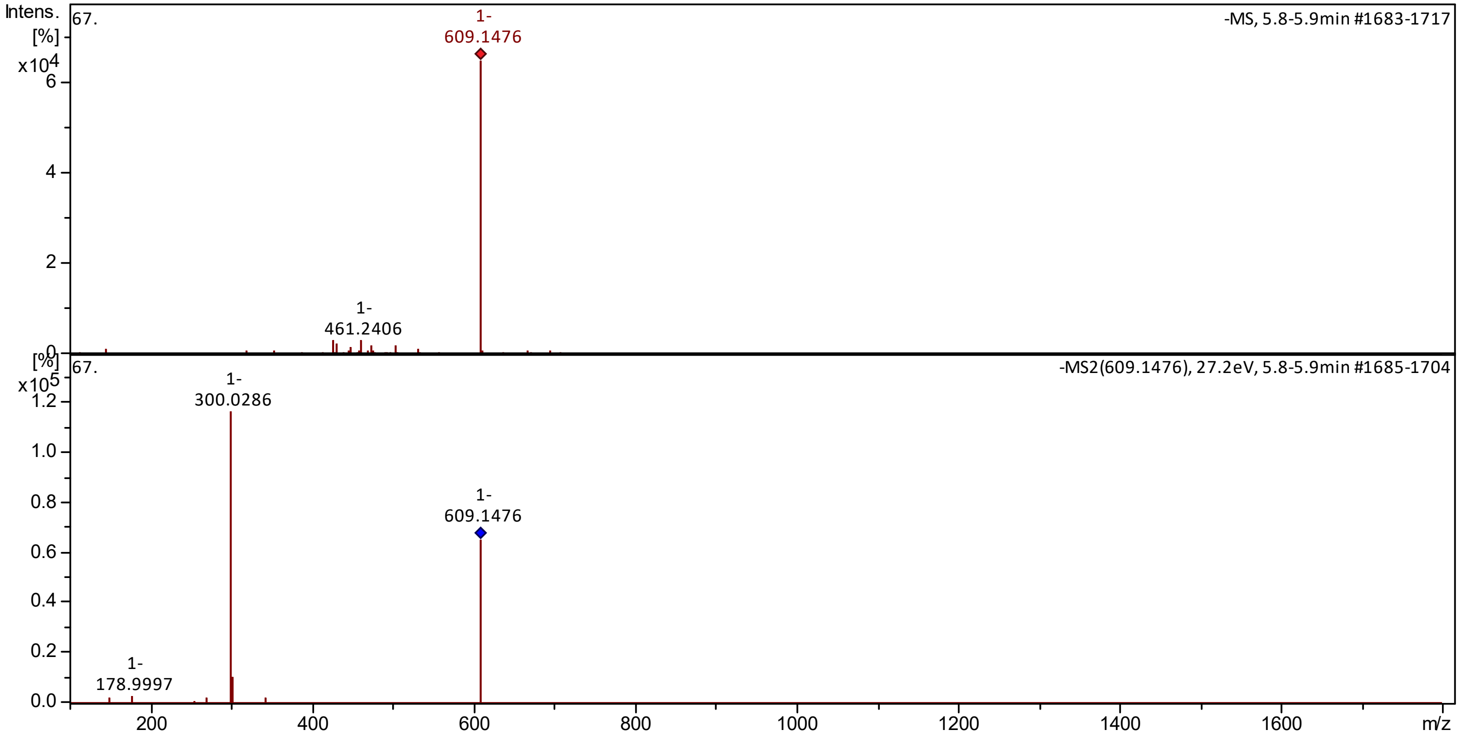

3,5-O- dicaffeoylguinic acid RT=6.8

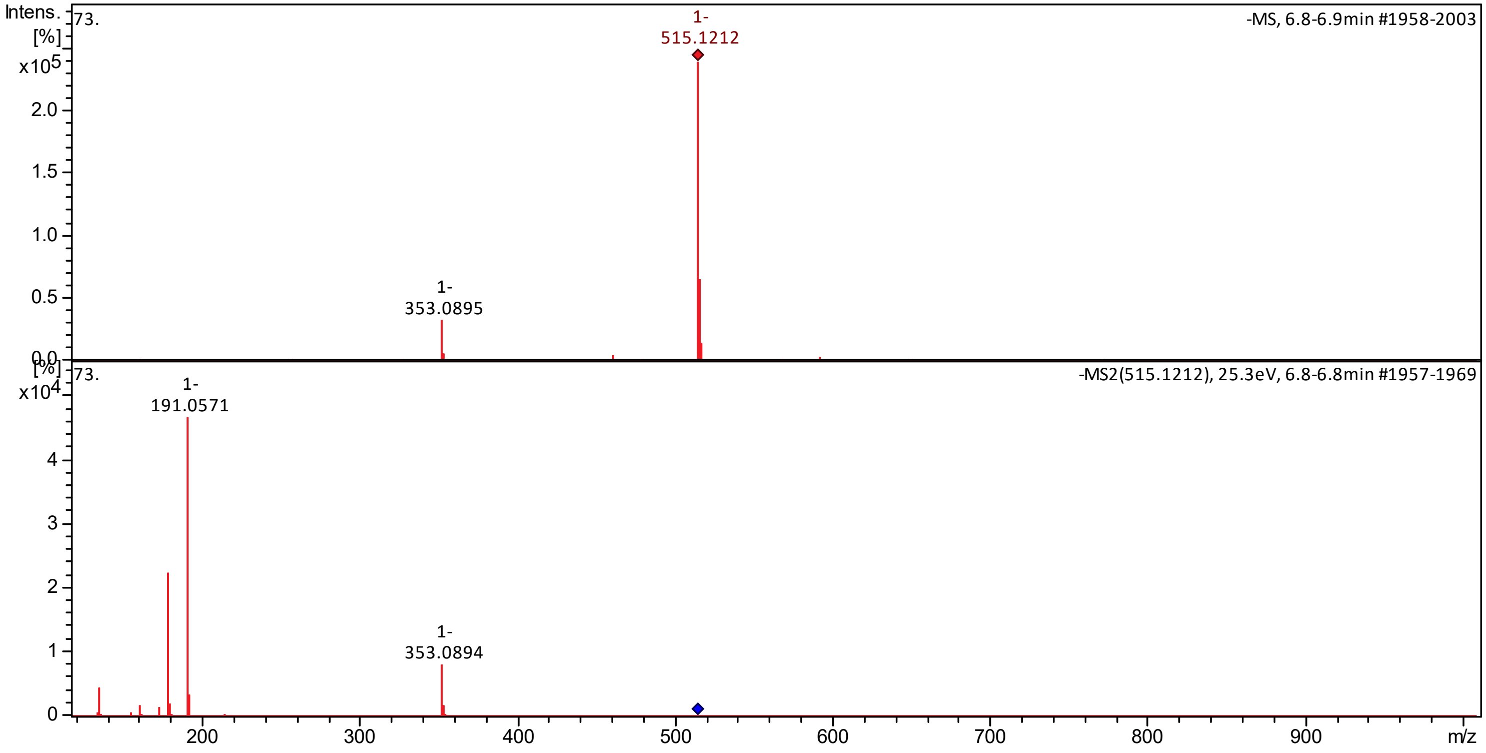

feruloylquinic acid derivative RT=7.3

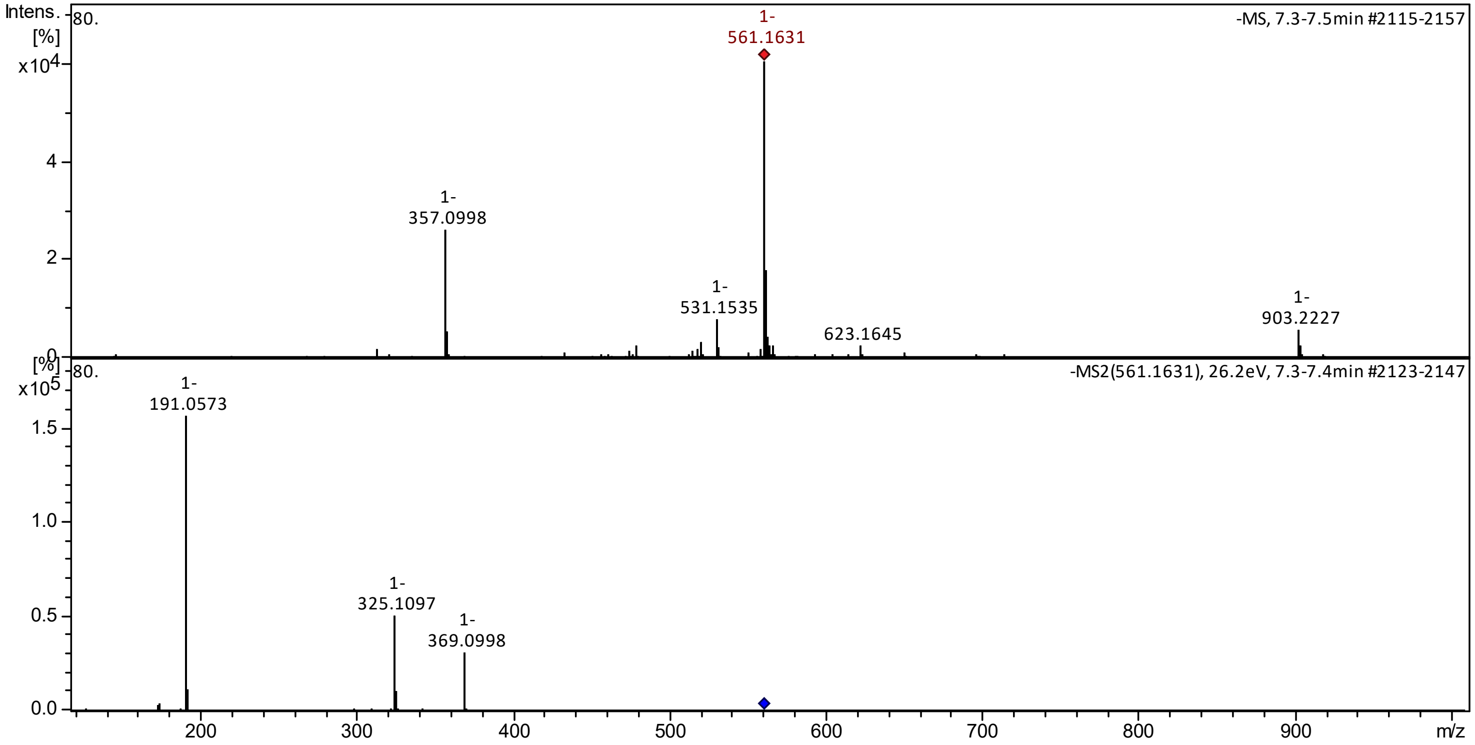

catechin derivative RT=11.7

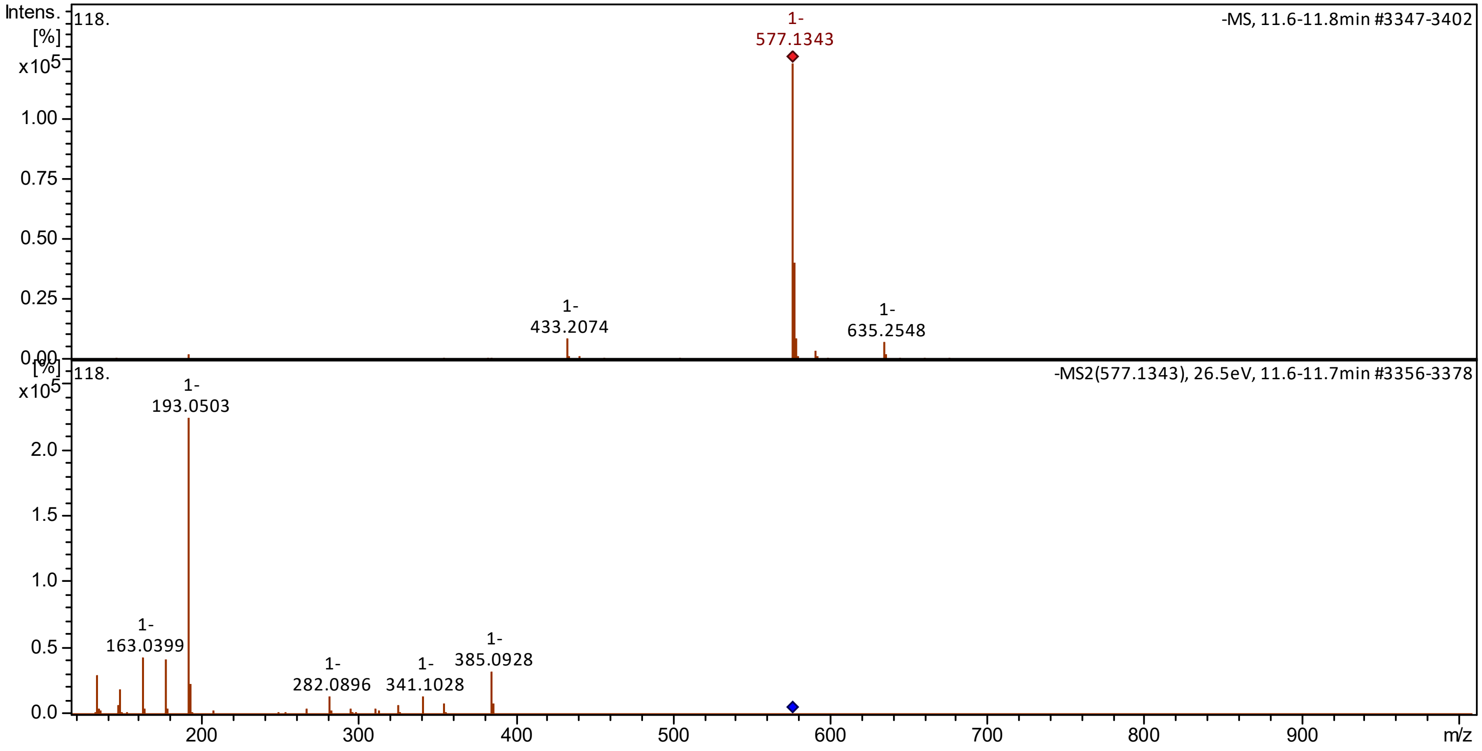

(+) - procyanidin B2 RT=13.0

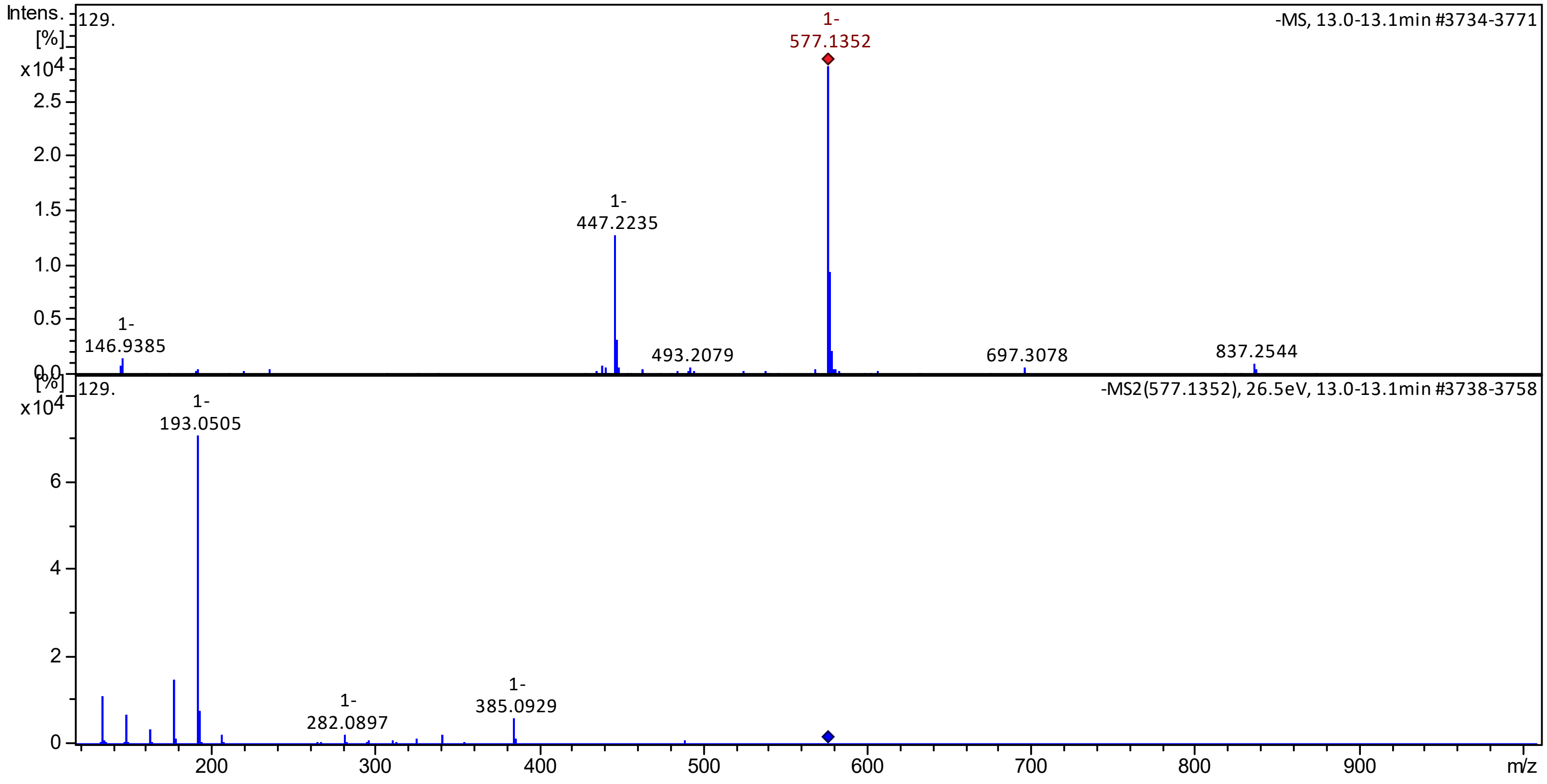

oleanane-type triterpenoid RT=14.0

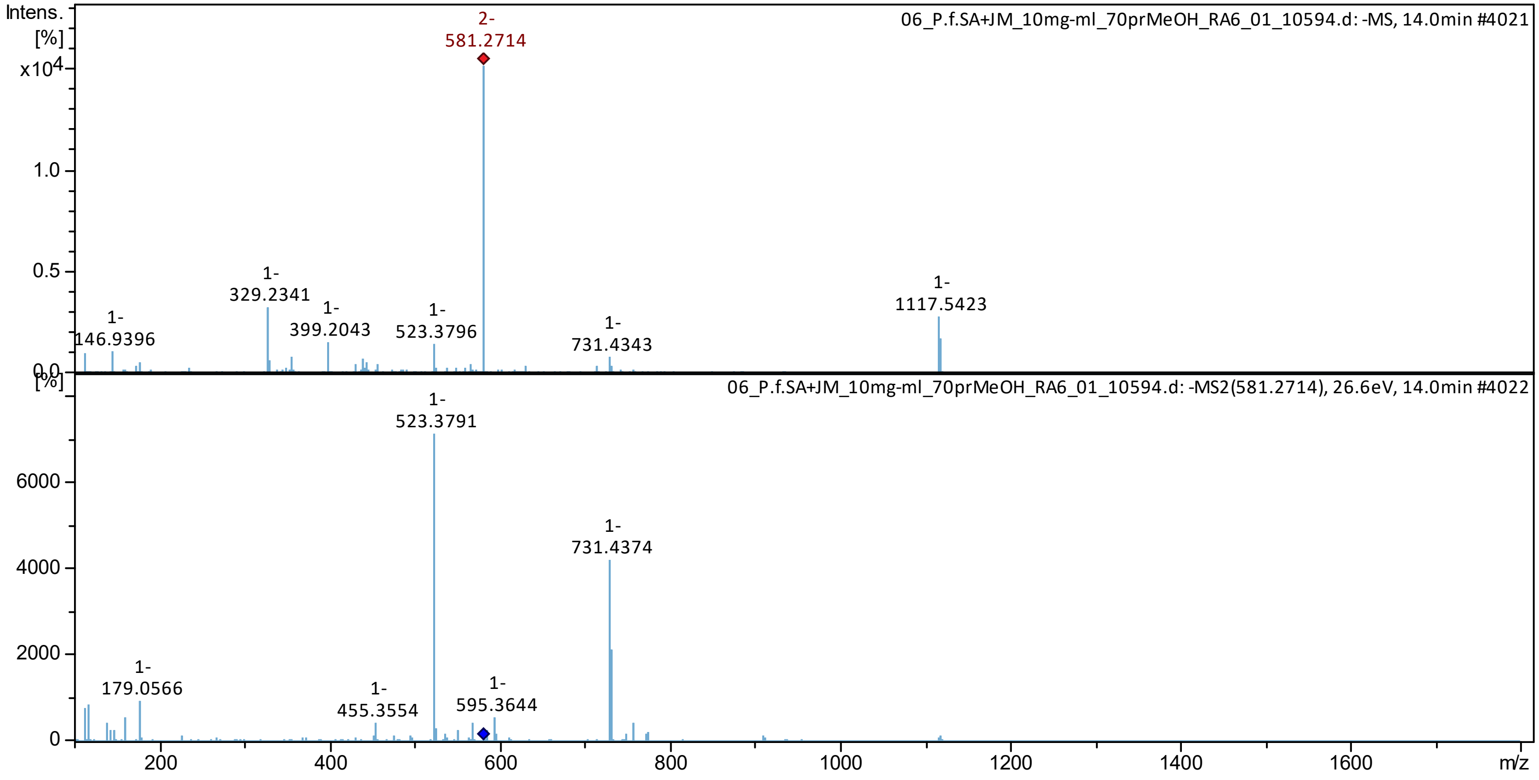

medicagenic acid- type triterpenoid RT=15.3

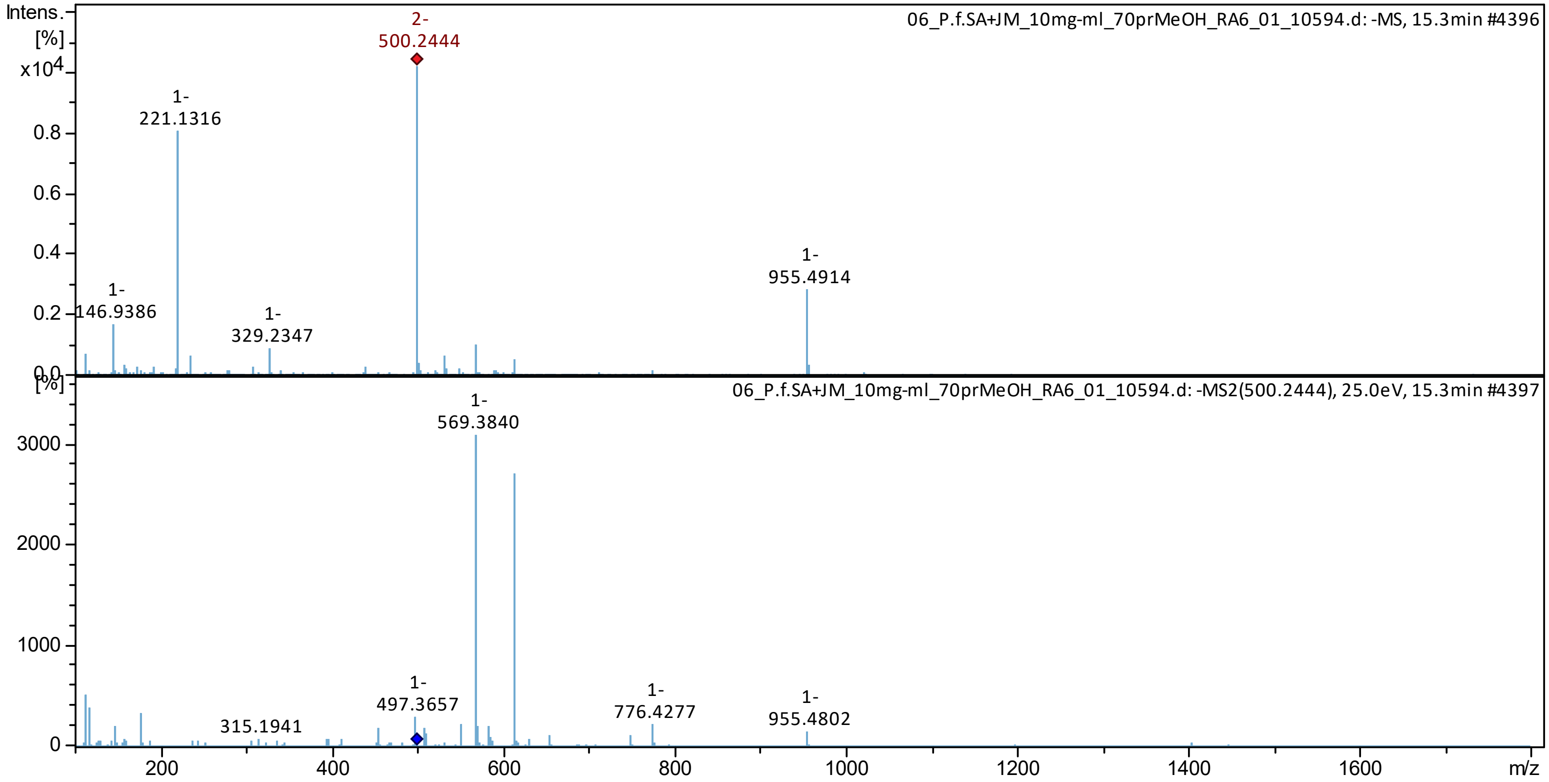

spinasaponin A/ zingibroside R1 (oleanane-type triterpenoid) RT=19.8

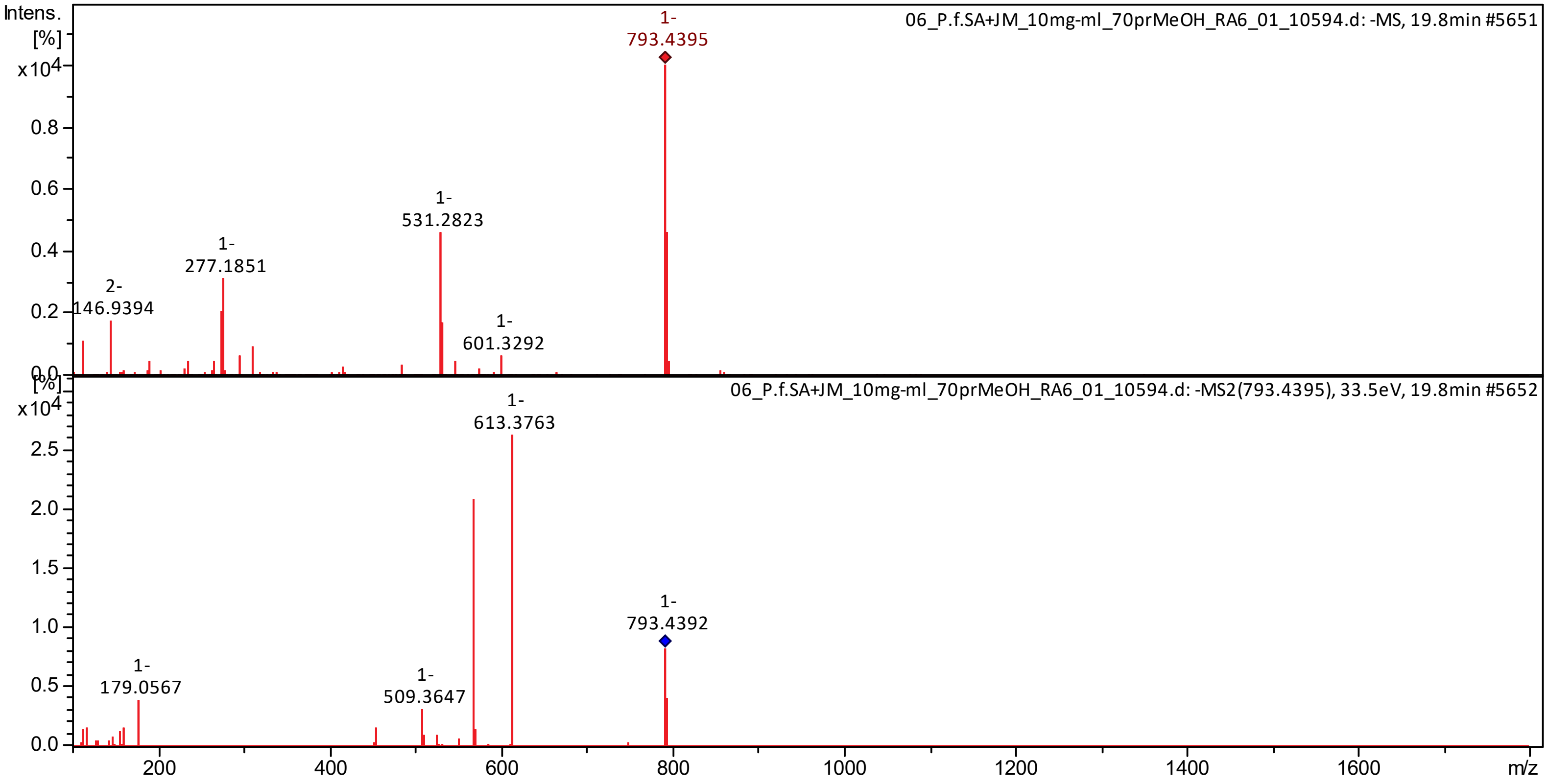

octadecanoic acid derivative RT=26.0

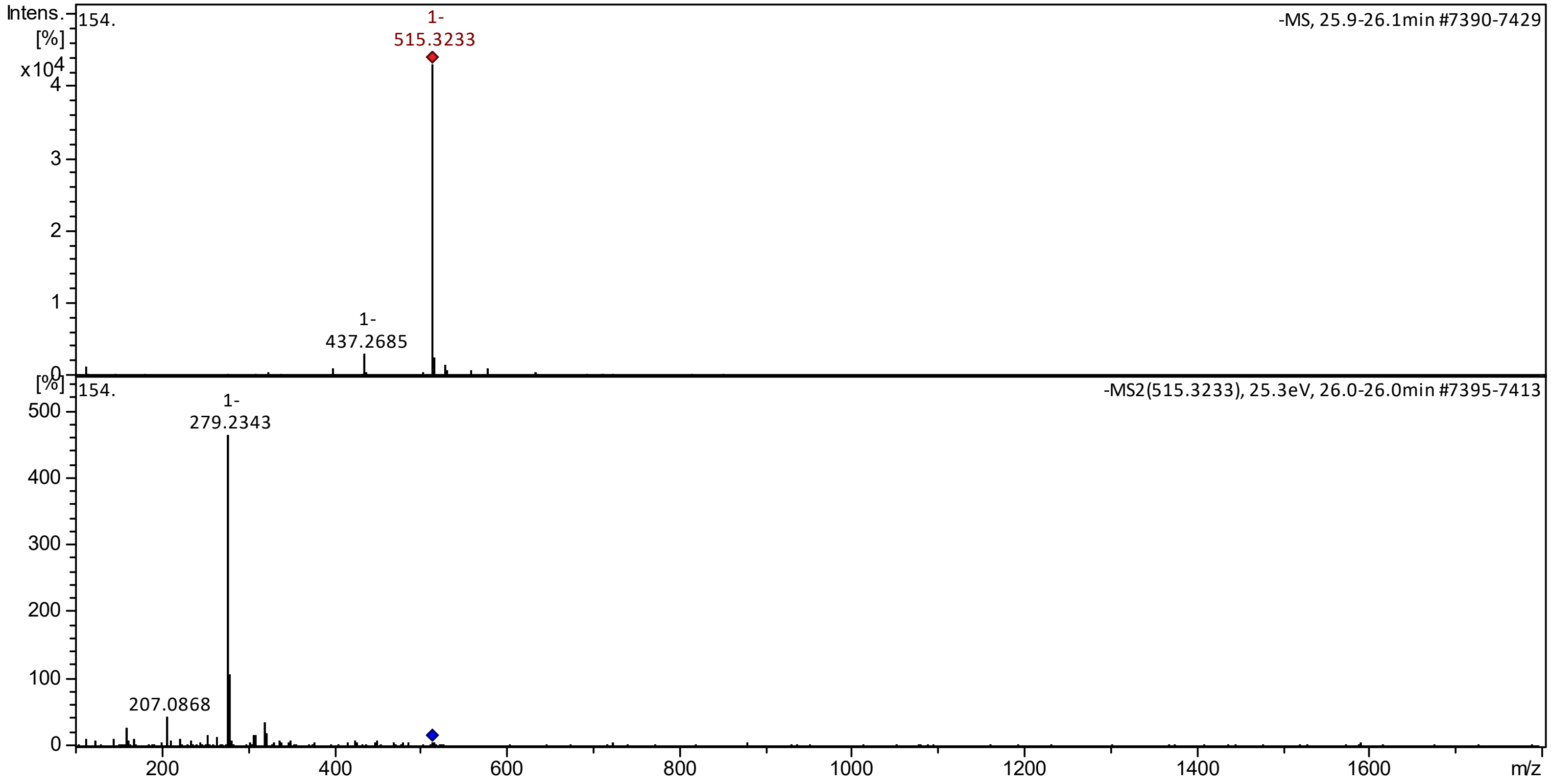

palmitate acid derivative RT=26.7

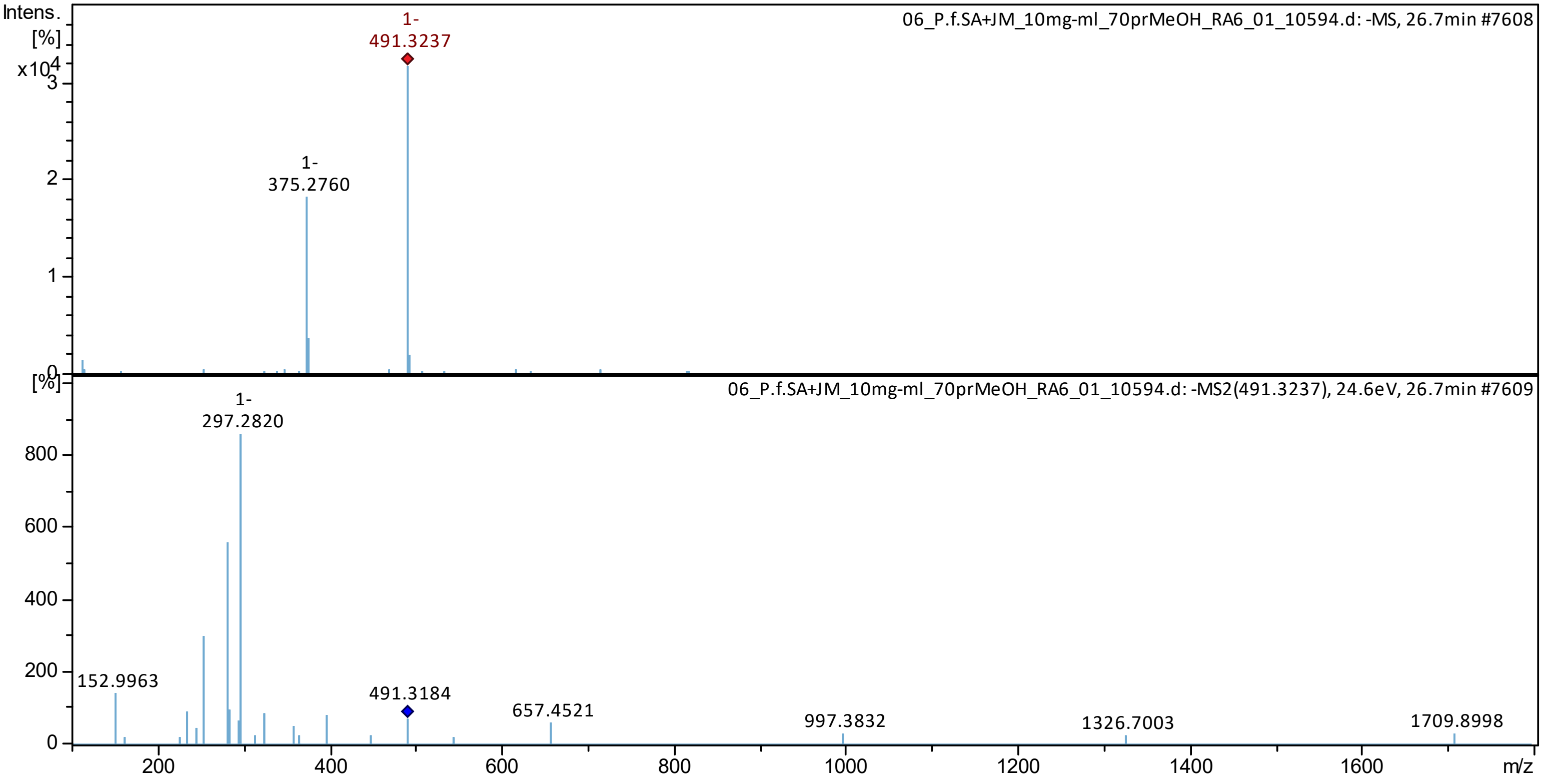

Figure 3S. MS spectra of the identified major compounds of plant extracts subjected to the investigation of antigenotoxic, anti-photogenotoxic and antioxidant properties a) methanolic extract of shoots cultivated under control conditions (EXT0); b)

methanolic extract of shoot elicited with salicylic acid 50 μM (EXT(SA); c) methanolic extract of shoots elicited with methyl jasmonate 200 μM (EXT(MeJA); d) methanolic extract of shoot elicited with salicylic acid 50 μM and methyl jasmonate 200 μM (EXT(MeJA+SA)).
